# Transcriptomic analysis of α-linolenic acid content and biosynthesis in *Paeonia ostii* fruits and seeds

**DOI:** 10.1186/s12864-021-07594-2

**Published:** 2021-04-23

**Authors:** Shui-Yan Yu, Xiao Zhang, Liang-Bo Huang, Yu-Ping Lyu, Ying Zhang, Zu-Jie Yao, Xiao-Xiao Zhang, Jun-Hui Yuan, Yong-Hong Hu

**Affiliations:** 1 Shanghai Key Laboratory of Plant Functional Genomics and Resources, Shanghai Chenshan Plant Science Research Center, Chinese Academy of Sciences, Shanghai Chenshan Botanical Garden, Shanghai, 201602 China; 2 Qingdao Agricultural University, Qingdao, 266109 Shandong China; 3 BGI-Shenzhen, Shenzhen, 518083 China

**Keywords:** Tree peony, Oil accumulation, Seed kernel, Seed testa, Fruit pericarp, Transcriptome sequencing, Gene expression

## Abstract

**Background:**

*Paeonia ostii* is a potentially important oilseed crop because its seed yield is high, and the seeds are rich in α-linolenic acid (ALA). However, the molecular mechanisms underlying ALA biosynthesis during seed kernel, seed testa, and fruit pericarp development in this plant are unclear. We used transcriptome data to address this knowledge gap.

**Results:**

Gas chromatograph-mass spectrometry indicated that ALA content was highest in the kernel, moderate in the testa, and lowest in the pericarp. Therefore, we used RNA-sequencing to compare ALA synthesis among these three tissues. We identified 227,837 unigenes, with an average length of 755 bp. Of these, 1371 unigenes were associated with lipid metabolism. The fatty acid (FA) biosynthesis and metabolism pathways were significantly enriched during the early stages of oil accumulation in the kernel. ALA biosynthesis was significantly enriched in parallel with increasing ALA content in the testa, but these metabolic pathways were not significantly enriched during pericarp development. By comparing unigene transcription profiles with patterns of ALA accumulation, specific unigenes encoding crucial enzymes and transcription factors (TFs) involved in de novo FA biosynthesis and oil accumulation were identified. Specifically, the bell-shaped expression patterns of genes encoding SAD, FAD2, FAD3, PDCT, PDAT, OLE, CLE, and SLE in the kernel were similar to the patterns of ALA accumulation in this tissue. Genes encoding BCCP, BC, KAS I– III, and FATA were also upregulated during the early stages of oil accumulation in the kernel. In the testa, the upregulation of the genes encoding SAD, FAD2, and FAD3 was followed by a sharp increase in the concentrations of ALA. In contrast, these genes were minimally expressed (and ALA content was low) throughout pericarp development.

**Conclusions:**

We used three tissues with high, moderate, and low ALA concentrations as an exemplar system in which to investigate tissue-specific ALA accumulation mechanisms in *P. ostii*. The genes and TFs identified herein might be useful targets for future studies of ALA accumulation in the tree peony. This study also provides a framework for future studies of FA biosynthesis in other oilseed plants.

**Supplementary Information:**

The online version contains supplementary material available at 10.1186/s12864-021-07594-2.

## Background

Edible oils for human consumption are mainly derived from plant seeds, which contain several types of fatty acids (FAs). Among the FAs, linoleic acid (LA; C18:2^Δ9,12^, a ω-6 FA) and α-linolenic acid (ALA; C18:3^Δ9,12,15^) are essential dietary nutrients for humans; these FAs cannot be synthesized independently by humans [1]. ω-6 FA and ALA not only exert a hypocholesterolemic effect against coronary heart disease and hypertension when used as human dietary supplements, but also are critical for fetal development and breastfeeding support [1, 2]. It has been suggested that a ratio of ω-6 to ω-3 FAs < 5 is optimal for human beings [3], and several sources have testaulated that ancient human diets had ω-6 to ω-3 FA ratios of ~ 1 [4]. Indeed, the high ratio of ω-6 to ω-3 FAs (~ 15:1) in the typical modern human diet is thought to be a major factor contributing to the high rates of cardiovascular disease in modern human societies [5]. One explanation for this imbalance is that the bulk of the oil seed crops consumed by modern humans, including soybean (*Glycine max*), peanut (*Arachis hypogaea*), maize (*Zea mays*), sunflower (*Helianthus annuus*), and rape (*Brassica napus*), have relatively low levels of ω-3 FAs, such as ALA [5]. However, tree peony (family Paeoniaceae, genus *Paeonia,* section *Moutan* DC.) seed oil, which is rich in ALA, has a ω-6 to ω-3 FA ratio of < 1.0 [6]. For this reason, tree peony seed oil has been recognized as a high-quality edible oil and was identified as a new food resource by the Chinese Ministry of Health in 2011 [7].

The tree peony, which was historically known as “The King of Flowers”, is a perennial shrub that is widely distributed in China [8]. There are nine wild species in section Moutan DC, which differ with respect to preferred habitat, but all rich in unsaturated FAs (> 90%) and ALA (26.7–50%) [6, 9]. To date, transcriptomic, proteomic, and microRNA sequencing have been used to investigate the mechanisms underlying ALA synthesis in tree peony seeds [10–14]. Previous studies have focused on enzymes that are key to ALA biosynthesis in various tree peony species, including FAD3 in *P. rockii*, *P*. *potaninii* and *P. lutea* seed [15]; FAD2 and FAD8 in *P. ostii* [10]; and ACCase*,* FATA*,* LPCAT*,* FADs*,* and DGAT in the developing endosperm of *P. ostii* var. lishizhenii [11]. Wang et al. [14] identified 115 genes and 24 proteins associated with the ALA metabolism in *P. ostii* seeds. However, despite these studies of FA metabolism in the tree peony, additional functional genes require further exploration.

In oil seed plants, stored lipids accumulate primarily as a result of FA synthesis in the plastids and triacylglycerol (TAG) assembly in the endoplasmic reticulum (ER). Acetyl-coenzyme A (CoA) is the unique building block used for FA production in plants [16]. In the plastid, de novo FA synthesis begins when acetyl-CoA is converted into malonyl-CoA by the acetyl-CoA carboxylase (ACC); malonyl-CoA is then either elongated to C16 and C18 by a series of enzymes or desaturated to C18:1-ACP by delta-9-stearoyl-ACP desaturase (SAD) [17, 18]. In most oilseed plants, more than 95% of the newly-synthesized FAs (primarily C18:1) are exported from plastids to the ER as CoA thioesters [19, 20]. The biosynthesis of polyunsaturated FAs (PUFAs) primarily relies on the further desaturation of C18:1 by omega-6 fatty acid desaturase (omega-6 FAD) and omega-3 FAD in the plastid or ER [16]. FAs, both those synthesized de novo or those have been modified through acyl editing, are assembled into glycerol-3-phosphate (G3P) to form TAG [21]. Generally, in plants and eukaryotic algae, TAG is mainly synthesized along the acyl-CoA-dependent Kennedy pathway and the acyl-CoA-independent pathway. Once TAGs have been synthesized, they are surrounded by a layer of phospholipids and amphipathic proteins, and then released to form oil bodies in the cytoplasm.

The FA metabolic pathway is complex, and the details of this pathway differ substantially among plant species and tissue types. In castor bean (*Ricinus communis*) and oil palm (*Elaeis guineensis*), transcriptome analyses showed that differences in FAs among seed tissues were associated with certain genes and transcription factors (TFs) [22, 23]. Interestingly, previous studies have showed that FA composition and relative abundance differ among wild *P. ostii* trees [6, 9], as well as among seed kernels and testas [6, 24]. This implies that FA synthesis and TAG assembly in developing tree peony seeds are differentially regulated among species. Zhang et al. [13, 15] compared FAD transcription (i.e., SAD, FAD2, and FAD3) among three species of tree peonies, and found that FAD transcription was greater in *P. rockii* seeds than in *P*. *potaninii* and *P. lutea* seeds. In addition, various transcriptomes of *P. ostii* seeds or endosperms are also available [10, 11, 14]. However, to date, no integrated analysis of the developing pericarp, seed coat, and endosperm in *P. ostii* has been performed.

In this study, we chose *P. ostii* as an exemplar tree peony species primarily because, compared with other tree peonies, *P. ostii* has higher seed yields and ALA content. This species is also suitable for large-scale cultivation south of the Yangtze River, China. In this study, we aimed to use comparative transcriptomics to assess the expression levels of certain genes associated with ALA accumulation during the development of three *P. ostii* tissues: the seed kernel, the seed testa, and the fruit pericarp. The identification of candidate genes that may affect levels of ALA accumulation will provide molecular tools for future genetic modifications, aimed at increasing the diversity and yield of seed oils produced by the tree peony and other oilseed plants.

## Results

### Morphological changes in developing *P. ostii* fruits and seeds

**Fig. 1:**
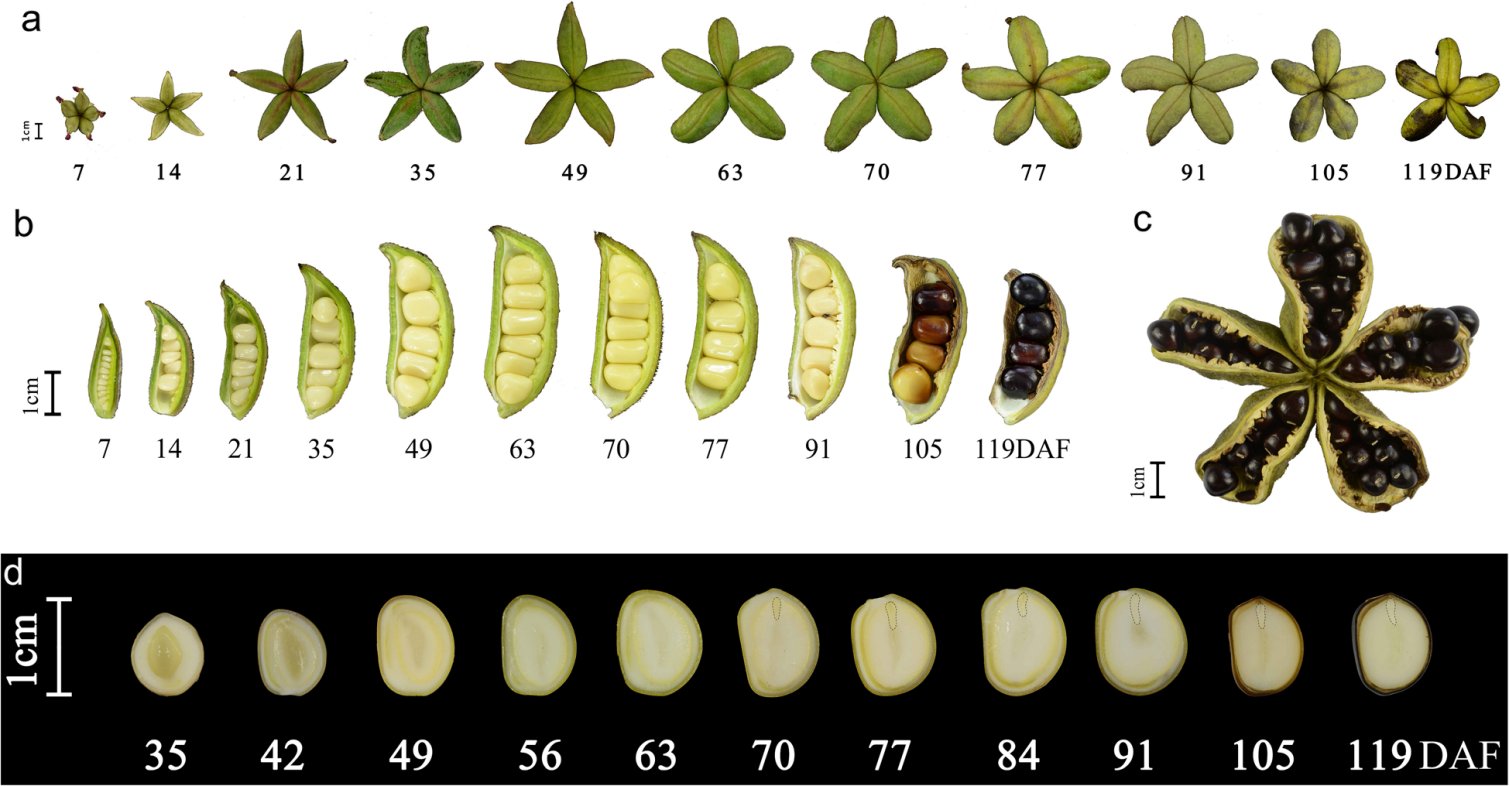
Development of fruits and seeds in *Paeonia ostii*. **a** Fruit development. **b** Seed and pod development. **c** Mature fruit. **d** Longitudinal sections showing seed development and embryogenesis. DAF, days after fertilization

At the study location (south of the Yangtze River), *P. ostii* fruit and seed development lasted about 120 days. *P. ostii* fruits and seeds grew rapidly between 7 and 49 days after fertilization (DAF), and at 49 DAF were almost as large as the mature forms (Fig. 1a–c). The fruit peel was green during the early stages of development but began to turn yellow at about 70 DAF (Fig. 1a). Examination of the seeds in longitudinal section indicated that embryogenesis and seed-filling occurred from 0 to 70 DAF; cotyledon embryos were obvious at 70 DAF. Between 70 and 119 DAF, the seed coat became thinner and browner as compared to the period from 49 to 70 DAF (Fig. 1d). At 119 DAF, the seeds were mature (Fig. 1b, d).

### Dynamic changes in FA composition in the pericarp, kernel, and testa

**Fig. 2:**
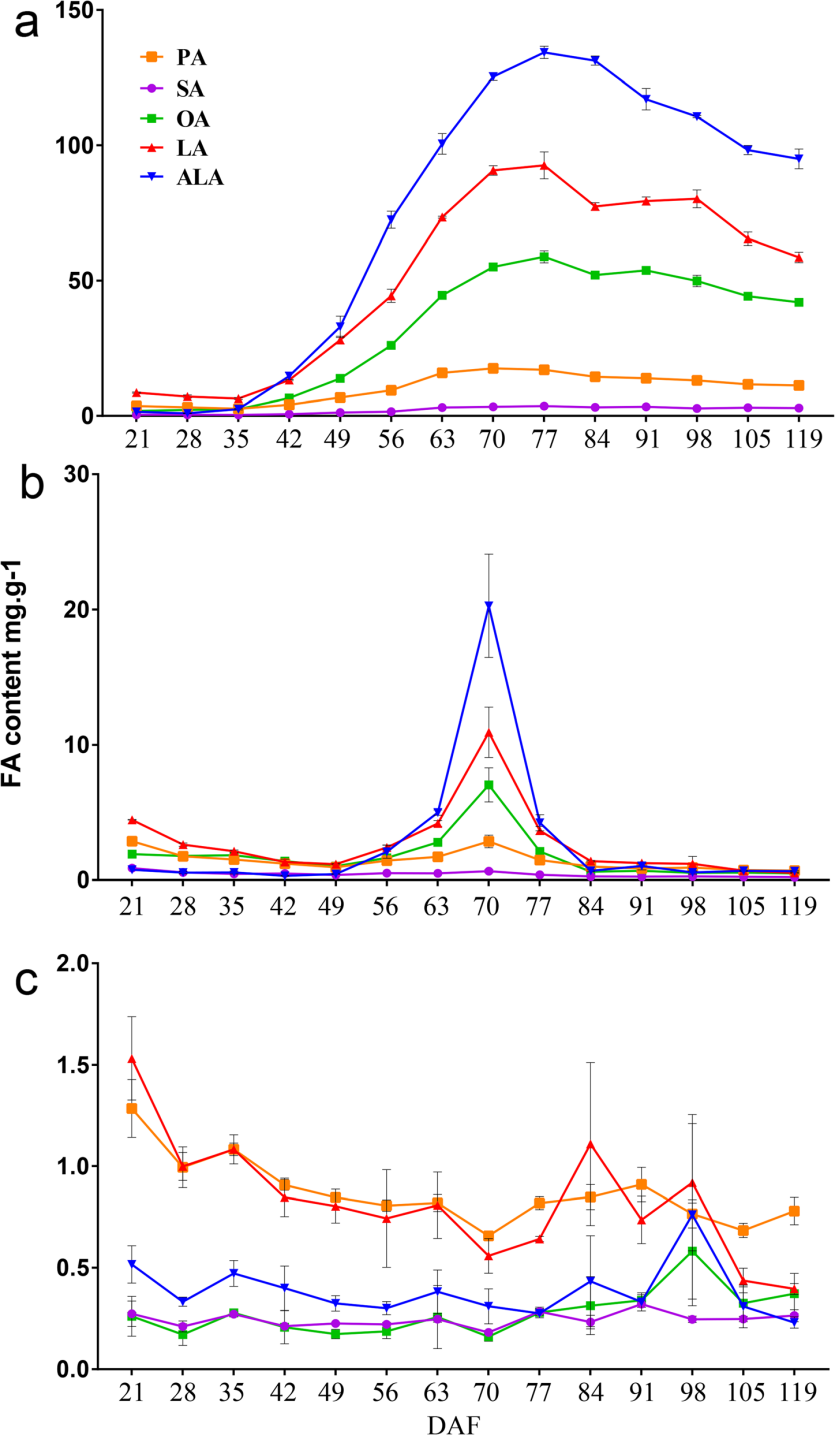
Fatty acid (FA) composition of *Paeonia ostii* fruits and seeds during development. **a** Seed kernel. **b** Seed testa. **c** Fruit pericarp. ALA, α-linolenic acid; LA, linoleic acid; OA, oleic acid; PA, palmic acid; SA, stearic acid

All five major FAs [ALA, LA, oleic acid (OA, C18:1^Δ9^), stearic acid (SA, C18:0) and palmitic acid (PA, C16:0)] were detected in the pericarp, testa, and kernel samples taken between 21 and 119 DAF (Fig. 2). The three most abundant UFAs during kernel development were OA, LA, and ALA. In the kernel, OA, LA, and ALA concentrations were 2.4 ± 0.08 mg/g, 6.35 ± 0.34 mg/g, and 2.5 ± 0.24 mg/g, respectively, at 35 DAF, increasing to 58.79 ± 2.21 mg/g, 92.56 ± 4.98 mg/g, and 134.29 ± 2.23 mg/g, respectively, at 77 DAF (Fig. 2a). After 77 DAF, OA, LA, and ALA concentrations decreased gradually, to 52 ± 0.73 mg/g, 77.36 ± 1.39 mg/g, and 131.26 ± 1.68 mg/g, respectively, at 84 DAF, and to 41.96 ± 1.21 mg/g, 58.56 ± 1.94 mg/g, and 94.96 ± 3.64 mg/g, respectively, at 119 DAF (Fig. 2a). Although the concentrations of all five FAs were similar throughout most of testa development (21–63 DAF and 77–119 DAF; Fig. 2b), ALA, LA, and OA peaked sharply at 70 DAF, with ALA concentration showing the greatest increase (to 20.27 ± 3.08 mg/g). FA concentrations fluctuated but remained low throughout pericarp development; PA and LA were the most abundant FAs (Fig. 2c). In all three tissues, SA concentrations remained consistently low.

### Unigene assembly, analysis, and quantification of gene expression

The transcriptomes of the kernel, testa, and pericarp samples taken at 35, 49, 63, 77, 91, and 119 DAF (T1–T6) from two separate trees (specimens CS0009 and CS0016) were sequenced. Across the 36 samples, we obtained approximately 49.69 million clean reads per sample (Table 1). A total 227,837 unigenes were detected, with an average length of 755 bp (Additional file 1: Table S1). The heatmap of the Pearson correlation coefficients of gene expression levels between pairs of genes showed that the gene expression levels were significantly correlated throughout pericarp development and during rapid oil accumulation in the kernel (Additional file 2: Figure S1). Across the six developmental stages, more genes were co-expressed in all three tissues than were expressed only in one tissue (Additional file 3: Figure S2). In addition, more genes were expressed specifically in the pericarp than in either the kernel or the testa. More genes were specifically expressed in the seed kernel at 49 DAF than at any other stage (Additional file 3: Figure S2).

**Table 1:** Sequencing statistics for the 36 samples taken during the development of *Paeonia ostii* seeds

Sample	Total Raw Reads (Mb)	Total Clean Reads (Mb)	Total Clean Bases (Gb)	Clean Reads Q20 (%)	Clean Reads Q30 (%)	Clean Reads Ratio (%)
CS0009_p_35	65.12	51.42	7.71	97.09	91.51	78.96
CS0009_p_49	53.48	41.89	6.28	97.08	91.53	78.32
CS0009_p_63	79.5	63.54	9.53	97.22	91.84	79.92
CS0009_p_77	85.27	63.95	9.59	96.95	91.22	74.99
CS0009_p_91	75.27	59.6	8.94	97.1	91.58	79.18
CS0009_p_119	79.37	58.11	8.72	97	91.2	73.22
CS0009_t_35	42.71	33.45	5.02	97.35	91.95	78.32
CS0009_t_49	68.7	51.46	7.72	96.62	90.23	74.9
CS0009_t_63	56.31	43.43	6.51	97.09	91.59	77.12
CS0009_t_77	56.61	41.72	6.26	96.93	91.16	73.69
CS0009_t_91	65.53	48.97	7.35	96.94	91.21	74.73
CS0009_t_119	49.76	41.65	6.25	98.13	94.01	83.71
CS0009_k_35	51.22	40.63	6.1	97.12	91.56	79.34
CS0009_k_49	55.96	44.77	6.72	97.22	91.84	80.01
CS0009_k_63	80.62	61.78	9.27	96.94	91.19	76.63
CS0009_k_77	56.87	42.97	6.45	96.99	91.24	75.55
CS0009_k_91	66.07	48.77	7.32	96.86	91.06	73.82
CS0009_k_119	58.84	43.22	6.48	96.76	90.8	73.45
CS0016_p_35	64.68	49.97	7.49	96.95	91.25	77.25
CS0016_p_49	70.79	55.9	8.38	97.05	91.41	78.95
CS0016_p_63	59.62	46.02	6.9	96.92	91.18	77.19
CS0016_p_77	72.86	56.46	8.47	96.99	91.3	77.48
CS0016_p_91	81.99	64.04	9.61	96.95	91.24	78.1
CS0016_p_119	42.01	31.77	4.77	96.89	90.75	75.62
CS0016_t_35	104.07	83.87	12.58	97.81	93.25	80.59
CS0016_t_49	66.34	50.45	7.57	97.06	91.51	76.04
CS0016_t_63	61.83	48.98	7.35	97.06	91.39	79.22
CS0016_t_77	73.54	57.14	8.57	97.15	91.65	77.71
CS0016_t_91	61.93	47.81	7.17	97.19	91.81	77.2
CS0016_t_119	109.95	81.97	12.3	97.34	92.47	74.56
CS0016_k_35	52.81	41.03	6.15	97	91.31	77.69
CS0016_k_49	61.03	48.59	7.29	97.2	91.78	79.61
CS0016_k_63	52.57	39.83	5.97	96.97	91.28	75.76
CS0016_k_77	57.55	48.62	7.29	98.16	94.06	84.47
CS0016_k_91	50.84	37.42	5.61	96.84	90.95	73.6
CS0016_k_119	47.16	34.85	5.23	96.75	90.75	73.9

### Ontology and KEGG pathway enrichment of the differentially expressed genes

**Fig. 3:**
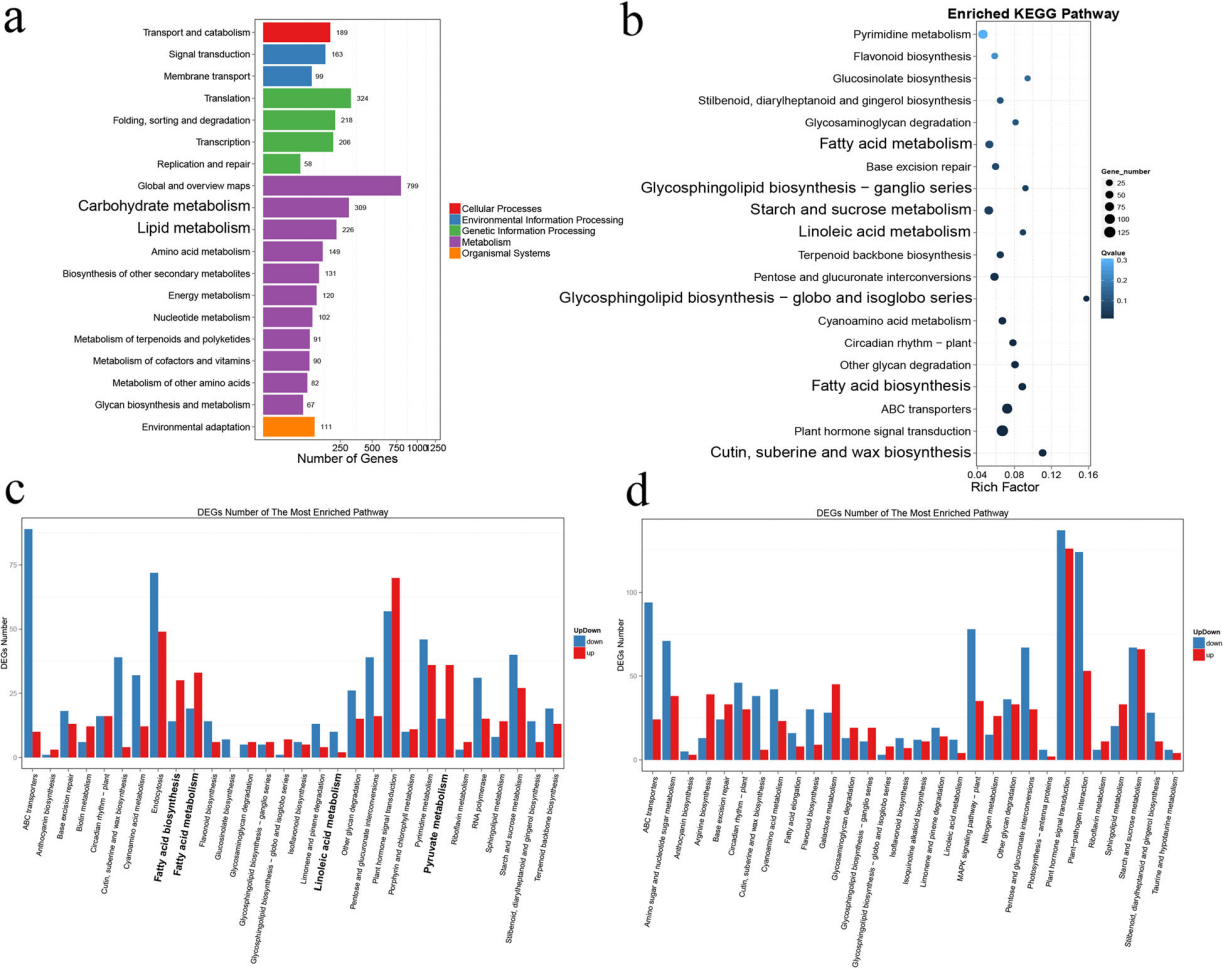
KEGG pathways enriched in the genes differentially expressed during the development of the *Paeonia ostii* seed kernel. **a** The number of genes differentially expressed between 35 and 49 DAF associated with various KEGG pathways. **b** The KEGG pathways overrepresented in the genes differentially expressed between 35 and 49 DAF. The color of each dot reflects the Qvalue, while the size of the dot represents the number of DEGs. **c** The up- and downregulated genes at 49 DAF, as compared to 35 DAF, that were associated with each KEGG pathway. **d** The up- and downregulated genes at 63 DAF, as compared to 35 DAF, that were associated with each KEGG pathway

The up- and downregulated genes at each of the six time points in the kernel, testa, and pericarp are shown in Additional file 4: Table S2. We found that the proportion of genes up- and downregulated varied among tissues and time points. The heatmap of the hierarchical DEG clusters indicated that more genes were differently expressed during the first three stages of kernel development (Additional file 5: Figure S3). We then determined which gene ontology (GO) functions were enriched in the DEGs. GO functions are grouped in three categories: molecular function, cell component and biological process. At 49 DAF, twice as many genes in the kernel and testa were enriched in metabolic process as compared to the pericarp (Additional file 6: Figure S4). In the kernel, 49 DAF was a period of rapid oil accumulation (Fig. 2a). Consistent with this, the genes differentially expressed in the kernel at 49 DAF as compared to 35 DAF were primarily associated with the lipid metabolism (226 DEGs) and the carbohydrate metabolism (309 genes; Fig. 3a). Other metabolic pathways, (e.g., fatty acid metabolism, fatty acid biosynthesis, linoleic acid metabolism, glycosphingolipid biosynthesis-ganglioseries, glycosphingolipid synthesis-globo and isoglobe series, and starch and sucrose metabolism) were also significantly enriched in these DEGs (Fig. 3b). Most of the metabolic pathways were overrepresented in the genes downregulated between 49 DAF and 35 DAF, including the linoleic acid metabolism pathway; linoleic acid is the substrate of ALA synthesis (Fig. 3c). However, the fatty acid metabolism, fatty acid biosynthesis, pyruvate metabolism, and plant hormone signal transduction pathways were overrepresented in the genes upregulated between 49 DAF and 35 DAF (Fig. 3c). With respect to the genes differently expressed between 63 DAF and 49 DAF, more pathways were significantly enriched in the downregulated genes than in the upregulated genes (Fig. 3d). Interestingly, the fatty acid metabolism and fatty acid biosynthesis pathways, which were significantly enriched at 49 DAF, were not significantly enriched at any other time point (Additional file 7: Figure S5).

**Fig. 4:**
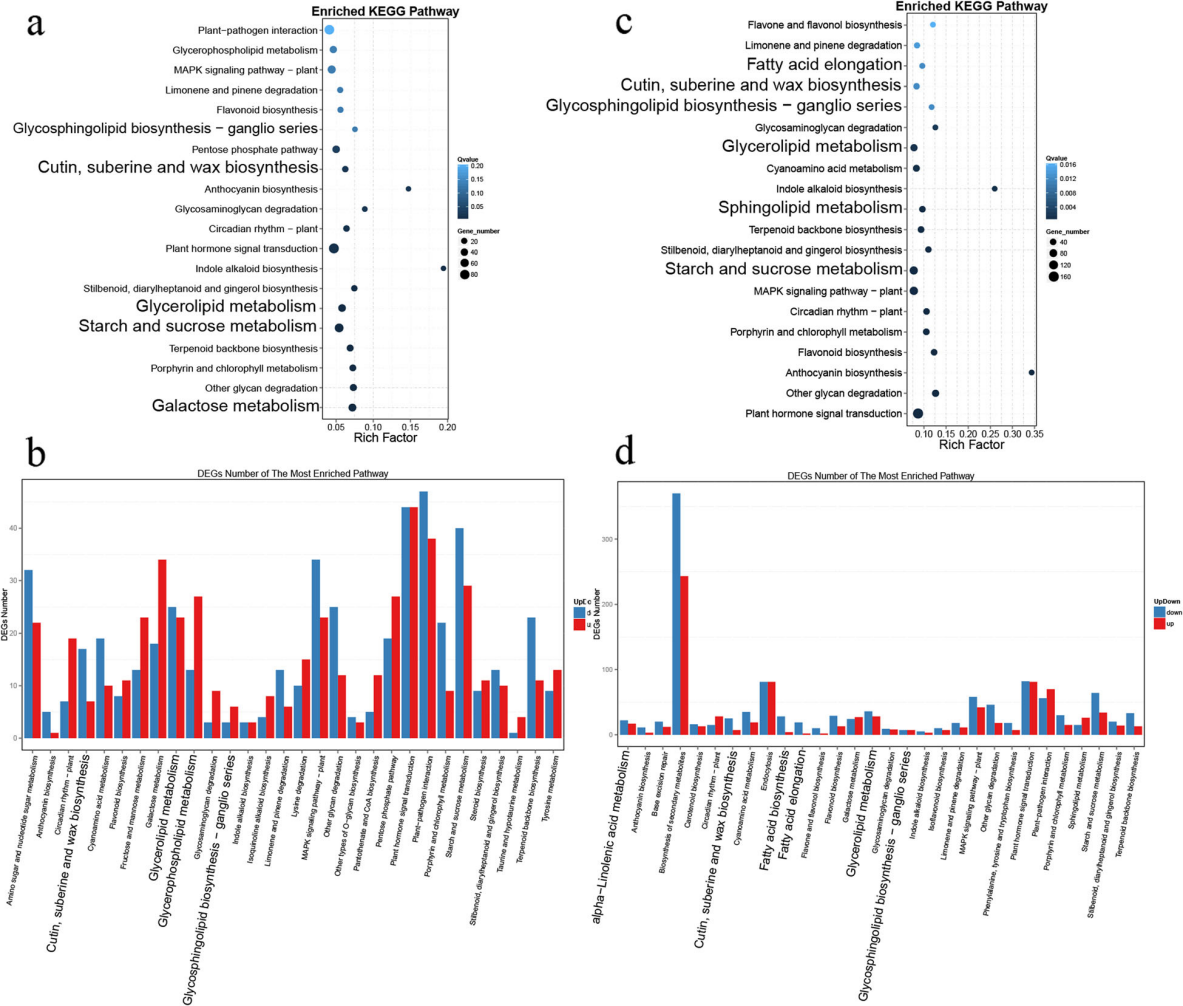
KEGG pathways enriched in the genes differentially expressed during the development of the *Paeonia ostii* seed testa. **a** The KEGG pathways overrepresented in the genes differentially expressed between 35 and 49 DAF. The color of each dot reflects the Qvalue, while the size of the dot represents the number of DEGs. **b** The up- and downregulated genes at 49 DAF, as compared to 35 DAF, associated with each KEGG pathway. **c** The KEGG pathways overrepresented in the genes differentially expressed between 35 DAF and 63 DAF. **d** The up- and downregulated genes at 63 DAF, as compared to 35 DAF, associated with each KEGG pathway

In the testa, 187 genes associated with the lipid metabolism were differentially expressed between 49 DAF and 35 DAF. Pathways related to the lipid metabolism (i.e., glycerolipid metabolism, glycerophospholipid metabolism, glycosphingolipid biosynthesis-ganglio series, and cutin/suberine and wax biosynthesis) and to the carbohydrate metabolism (i.e., starch/sucrose metabolism and galactose metabolism) were significantly enriched in these DEGs (Fig. 4a). The glycerolipid metabolism pathway was overrepresented in similar numbers of up- and downregulated DEGs at 49 DAF (Fig. 4b). Several other lipid-associated pathways (i.e., glycerophospholipid metabolism, glycosphingolipid biosynthesis-ganglio series, and steroid biosynthesis) were more enriched in the upregulated DEGs than in the downregulated DEGs at 49 DAF (Fig. 4b). Similarly, pathways related to starch accumulation and sugar synthesis (i.e., fructose/mannose metabolism and galactose metabolism) were also enriched in more upregulated than downregulated DEGs at 49 DAF (Fig. 4b). This might be due to the intensive membrane lipid synthesis, seed coat thickening, and dry matter accumulation that occurs during this stage in the testa. Compared with 35 DAF, 287 genes associated with the lipid metabolism were differently expressed at 63 DAF in the testa; the metabolic pathways significantly enriched in these DEGs included fatty acid elongation, glycerolipid metabolism, sphingolipid metabolism, glycosphingolipid biosynthesis-ganglio series, and cutin/suberine and wax biosynthesis (Fig. 4c). At 63 DAF, most of the DEGs were associated with the alpha linolenic acid pathway; in this pathway, the number of upregulated genes was similar to the number of downregulated genes (Fig. 4d). No lipid metabolism pathways were enriched at any other stage of testa development (Additional file 8: Figure S6).

In the pericarp, certain lipid metabolism pathways, including glycerolipid metabolism, fatty acid elongation, sphingolipid metabolism, and cutin/suberine and wax biosynthesis, were similarly enriched in all DEGs across all stages (Additional file 9: Figure S7 and Additional file 10: Figure S8).

### Expression patterns of unigenes associated with oil accumulation

Based on the functional annotations of the DEGs, we identified 1373 unigenes associated with lipid metabolism (Additional file 11: Table S3). These unigenes were associated with 10 metabolic pathways: de novo plastid FA synthesis, elongation, desaturation and export (138 unigenes); triacylglycerol biosynthesis and eukaryotic phospholipid synthesis & editing (176 unigenes); prokaryotic and eukaryotic galactolipid, sulfolipid, and phospholipid synthesis (25 unigenes); sphingolipid biosynthesis (58 unigenes); fatty acid elongation, wax biosynthesis, cutin synthesis, and suberin synthesis and transport (251 unigenes); triacylglycerol and fatty acid degradation (99 unigenes); phospholipid signaling (108 unigenes); lipid trafficking (27 unigenes); oxylipin metabolism (39 unigenes); and mitochondrial fatty acid synthesis (43 unigenes) (Additional file 12: Table S4).

**Fig. 5:**
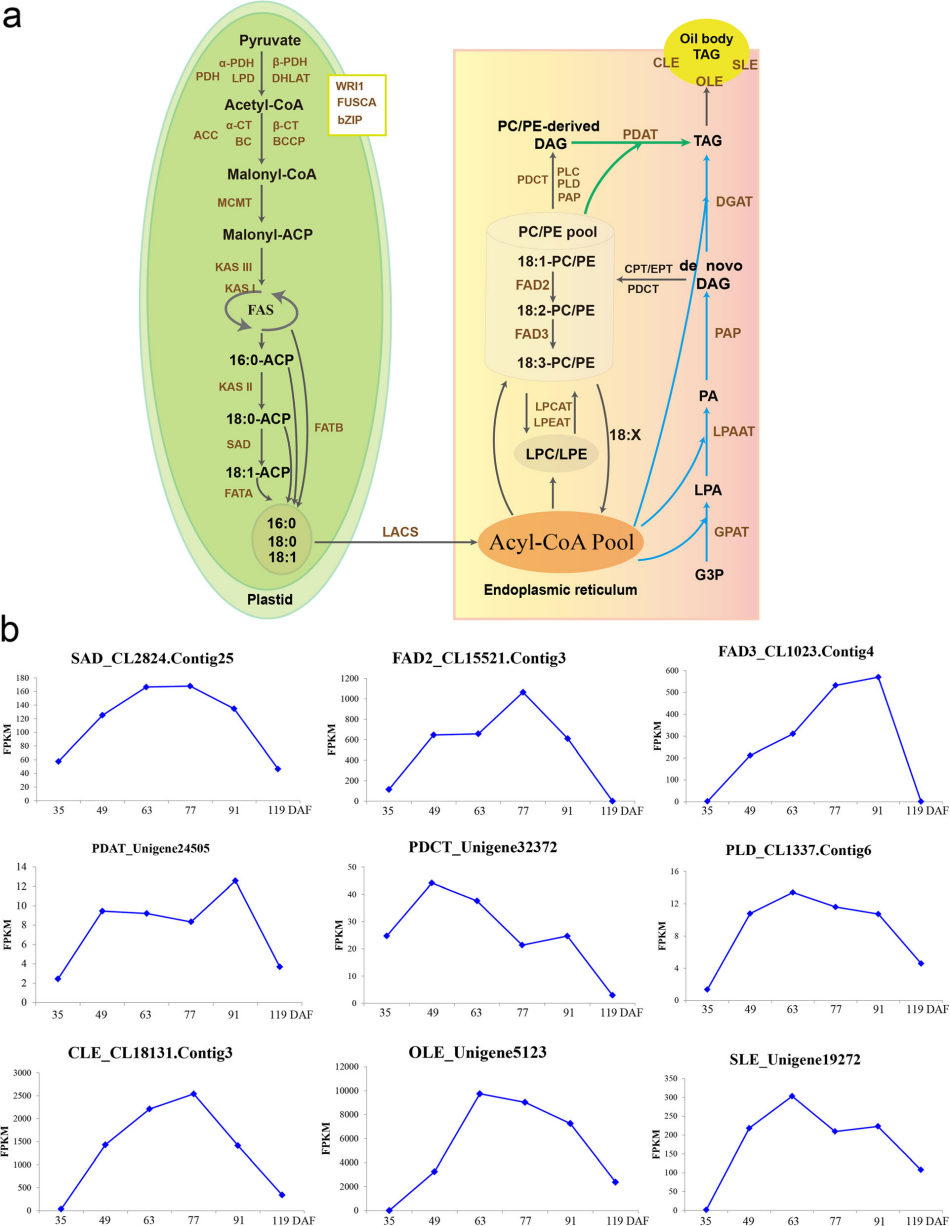
Genes putatively associated with the lipid metabolism in *Paeonia ostii*. **a** Lipid metabolism in *P. ostii*, showing putative unigenes associated with FA biosynthesis, TAG biosynthesis, and oil formation. **b** Expression patterns of key genes associated with ALA biosynthesis in the *P. ostii* seed kernel

Of these 1373 unigenes, 314 were associated with FA and TAG biosynthesis (Additional file 12: Table S4). Specifically, 13 unigenes were associated with the pyruvate dehydrogenase complex (PDHC), which catalyzes the oxidative decarboxylation of pyruvate to produce acetyl-CoA. The PDHC contains three subunits: pyruvate dehydrogenase (PDH, 10 unigenes), dihydrolipoyl acyltransferase (DHLAT, 2 unigenes), and dihydrolipoamide dehydrogenase (LPD, 1 unigene). These unigenes were highly expressed throughout the development of the pericarp, testa, and kernel. In addition, 25 unigenes encoded subunits of ACC, a multi-subunit enzyme that includes biotin carboxylase (BC), the biotin carboxyl carrier protein (BCCP), and carboxyltransferase (CT). Finally, seven unigenes were homologous to α-CT; one unigene was homologous to β-CT; 13 unigenes were homologous to BC; and four unigenes were homologous to BCCP (Additional file 12: Table S4). In particular, CL15202, homologous to α-CT, was highly expressed across all three tissues, but was most strongly upregulated in the kernel. Similarly, CL9207.Contig2, homologous to homomeric ACC (HmACC), was also highly expressed during the development of all three tissues but was most highly expressed in the testa. CL16462 (homologous to α-CT) and unigene 719 (homologous to β-CT) were strongly upregulated in the pericarp but expressed only at low levels in the kernel and the testa. In addition, homologs to BC (CL7345.contig8) and BCCP (CL18970.contig3 and unigene17464) were significantly upregulated in the kernel at 35 and 49 DAF, as compared to later developmental stages (63–119 DAF); these genes were expressed only at low levels throughout the development of the testa and pericarp (Additional file 13: Figure S9). Three unigenes were associated with the catalysis of the malonyl-ACP elongation cycle: CL8489.Contig2, associated with 3-ketoacyl-ACP synthase isoform I (KAS I); CL1611.Contig2, associated with KAS II; and unigene32463, associated with KAS III. These unigenes were strongly upregulated during pericarp and testa development but were highly expressed in the kernel at 49 DAF only (Additional file 13: Figure S9). Eighteen unigenes encoding SAD were also identified as DEGs. Of these, four (CL2824.Contig13, CL2824.Contig14, CL2824.Contig25, and CL2824.Contig26) were more highly expressed in the kernel and the testa than in the pericarp (Additional file 13: Figure S9). In addition, in the kernel, the expression levels of CL2824.Contig25 steadily increased from 35 to 63 DAF, remained constant from 63 to 77 DAF, and decreased steadily from 77 to 119 DAF (Fig. 5). One unigene was shown to encode fatty acyl-ACP thioesterase A (FATA) (unigene17393), and three were shown to encode FATB (unigene2022, unigene5608, and unigene6721). Unigene6721 was differently expressed among the different developmental stages of the pericarp, testa, and kernel, but unigene17393 was only highly expressed in the kernel (with an expression peak at 49 DAF; Additional file 13: Figure S9). We also identified 10 unigenes encoding long-chain acyl-CoA synthetase (LACS) (Additional file 12: Table S4).

Ten unigenes were associated with PUFA biosynthesis: three unigenes encoded FAD2 (CL15521.Contig1, CL15521.Contig2, and CL15521.Contig3), and two unigenes encoded FAD6 (CL5945.Contig3 and CL5945.Contig8). In the kernel, the expression level of one of the FAD2-encoding genes (CL15521.Contig3) increased from 35 to 77 DAF, and decreased from 77 to 119 DAF (Fig. 5). This unigene was also sharply upregulated at 77 DAF in the testa (Additional file 13: Figure S9). An additional four unigenes encoded FAD3 and FAD8 (unigene2598, unigene43483, CL1023.Contig1, and CL1023.Contig4), and one unigenes encoded FAD7 (unigene21286) (Additional file 11: Table S3). Based on our phylogenetic analysis of ω-3 FADs from *Arabidopsis thaliana* and *P. ostii* (Additional file 14: Figure S10), we identified four microsomal FAD3 genes and one plastid FAD7/8 gene in *P. ostii*. In the kernel, the expression level of one of these FAD3-encoding genes (CL1023.Contig4) increased steadily from 39 to 91 DAF, followed by an abrupt decrease (Fig. 5). Similarly, CL1023.Contig4 was sharply upregulated at 77 DAF in the testa (Additional file 13: Figure S9). In contrast, CL1023.Contig4 was expressed only at low levels throughout pericarp development.

Several unigenes encoding enzymes important for acyl editing and the Lands cycle were also identified. The three unigenes encoding CDP-choline: DAG choline phosphotransferase (CPT) (unigene7809, unigene16109, and unigene16131) were differentially expressed during the development of the kernel, pericarp, and testa. One unigene, encoding phosphatidylcholine:diacylglycerol cholinephosphotransferase (PDCT) (unigene32372), was significantly differentially expressed among these tissues. In the kernel, unigene32372 expression increased from 35 to 49 DAF, and then decreased fairly steadily until the end of the experiment (Fig. 5). Three unigenes encoding lysophosphatidylcholineacyltransferase (LPCAT) (unigene39796, CL18110.Contig2, and CL18110.Contig24) and two unigenes encoding lysophosphatidylethanolamineacyltransferase (LPEAT) (CL10703.Contig3 and CL20149.Contig2) were identified as DEGs. We also identified 19 unigenes encoding PLD. In the kernel, the expression profile of one of these unigenes (CL1337.Contig6) resembled a bell-shaped curve, peaking at 63 DAF (Fig. 5). Other phospholipase D (PLD) genes were also identified as DEGs but did not exhibit a smooth peak in expression.

We identified 45 unigenes associated with TAG biosynthesis. Of these, 16 unigenes encoded glycerol-3-phosphate acyltransferase (GPAT), 10 unigenes encoded lysophosphatidic acid acyltransferase (LPAAT), 10 unigenes encoded phosphatidate phosphatase (PAP), and 4 unigenes encoded diacylglycerol acyltransferase (DGAT). Specifically, four unigenes were strongly upregulated in the kernel at 49 DAF as compared to 35 DAF: unigene42999 encoding GPAT; CL1842.Contig1 and CL1842.Contig2 encoding LPAAT; CL4004.Contig1 and CL18619.Contig1 encoding PAP; and CL5087.Contig1 encoding DGAT (CL5087.Contig1) (Additional file 13: Figure S9). This pattern was observed in neither the testa nor the pericarp. Five unigenes encoding phospholipid:diacylglycerol acyltransferase (PDAT) were identified as DEGs. One of these (CL7876.Contig4) was highly expressed throughout pericarp and testa development, but was downregulated at 49 DAF as compared to 35 DAF in the kernel. In the kernel, the expression of another PDAT-encoding unigene (unigene24505) increased dramatically from 35 to 49 DAF and then again from 77 to 91 DAF (Fig. 5). The expression levels of this gene in the testa and pericarp were very low (Additional file 13: Figure S9). We identified 29 unigenes associated with the formation of oil bodies in the cytoplasm that were also DEGs. Of these unigenes, six encoded oleosin (OLE), 19 encoded caleosin (CLE), and four encoded steroleosin (SLE) (Additional file 12: Table S4). All of these unigenes were stably expressed at very low levels during pericarp development, but were highly expressed throughout the development of the testa and very highly expressed throughout the development of the kernel (Additional file 13: Figure S9). In the kernel, bell-shaped expression profiles were observed for one CLE-encoding unigene (CL18131.Contig3), two OLE-encoding unigenes (unigene5123 and unigene17798), and one SLE-encoding unigene (CL19272.Contig1) (Fig. 5).

### TFs associated with FA and TAG biosynthesis

**Fig. 6:**
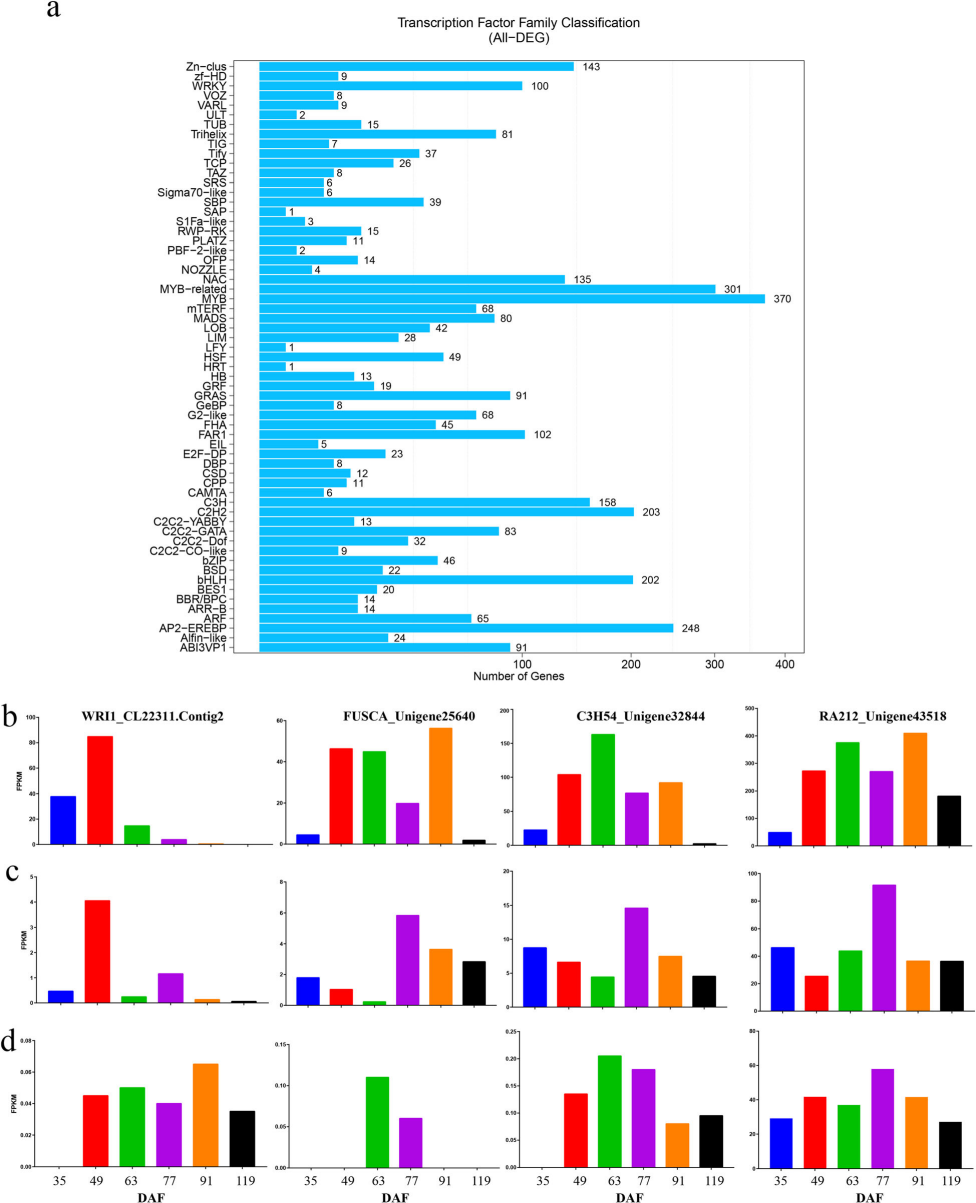
Expression profiles of *Paeonia ostii* unigenes that are also transcription factors (TFs). **a** The distribution of unigenes among TF families. **b, c, and d** The relative expression levels of genes associated with key TFs in the developing kernel (b), testa (c), and pericarp (d)

We predicted the DEGs that might encode TFs and classified these into TF families (Fig. 6a). Of these TF DEGs, 301 belonged to the MYB family, 164 to the AP2-EREBP family, 161 to the FAR1 family, 132 to the NAC family, 115 to the MADS family, 114 to the bHLH family, 70 to the WRKY family, and 14 to the bZIP family. Within the TF DEGs, we also identified the unigenes related to the FA metabolism: nine in the WRI family, 20 in the bZIP family, one in the LEAFY family, six in the FUSCA (FUS) family, five in the ABSCISIC ACID-INSENSITIVE family, and 82 in the MYB family (Additional file 15: Table S5).

WRI1 (CL22311) was more strongly upregulated in the kernel than in the testa and the pericarp, especially during the early stages of FA accumulation (Fig. 6b). FUSCA (unigene25640) was also more strongly upregulated in the kernel than in the pericarp or the testa (Fig. 6b). The two unigenes encoding bZIP (unigeneCL3725 and unigeneCL15124) were constitutively expressed in all three tissues (Additional file 16: Figure S11). The expression patterns of the many TF DEGs in the MYB family differed among tissues. One unigene (CL14921.Contig3) was highly expressed in the pericarp but expressed only at low levels in the kernel and testa. Similarly, unigene25046 was also differentially expressed among tissues. In the kernel, unigene25046 expression increased from 35 to 49 DAF, and then decreased sharply until the end of the experiment (Additional file 16: Figure S11). In the testa, unigene25046 was upregulated at 49 DAF, this high level of expression was maintained until the end of the experiment. In the pericarp, unigene25046 was expressed at low levels throughout the experiment. We also considered the unigenes encoding zinc finger CCCH domain-containing protein 54 (C3H54) and ethylene-responsive transcription factor (RA212). Unigene32844, encoding C3H54, and unigene43518, encodingRA212, were both upregulated during FA accumulation in the kernel, but were expressed only at low levels in the pericarp and testa (Fig. 6b).

### Quantitative real-time PCR (qRT-PCR) validation of key DEGs

**Fig. 7:**
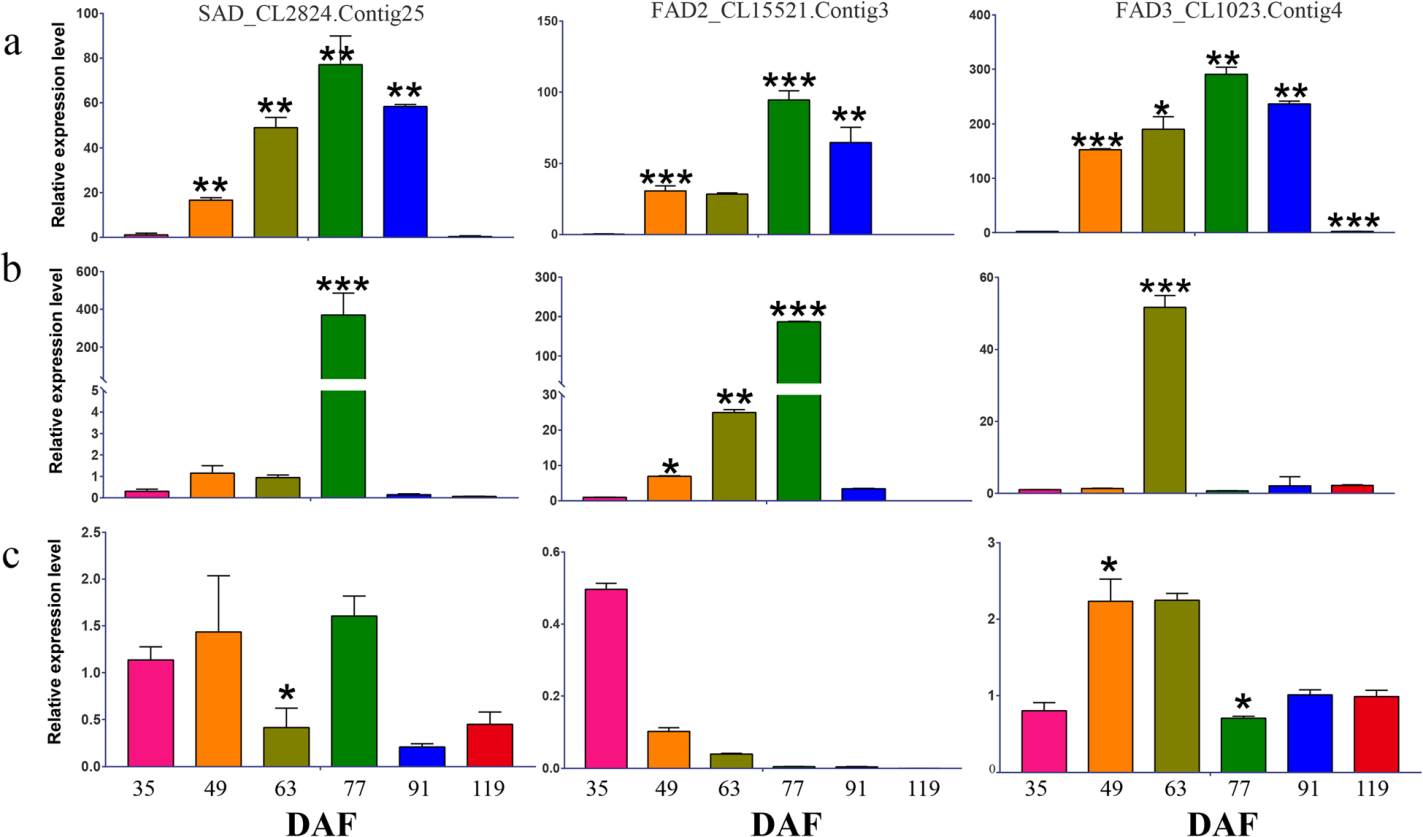
Quantitative real-time PCR validation of fatty acid desaturase genes. **a** Kernel. **b** Testa. **c** Pericarp. Actin was used as reference genes. Expression values were normalized such that the expression level at 21 DAF was set to 1. Values shown are means of three biological replicates; error bars indicate standard deviation. One-way ANOVAs were used to compare means among groups, and *p* value < 0.005 was calculated in the bar chart are shown in Fig. 7

We identified 1373 unigenes related to the lipid metabolism in *P. ostii*. Of these, 13 unigenes associated with FA biosynthesis and TAG assembly, especially ALA accumulation, were selected for qRT-PCR validation. In general, the expression patterns of the 13 key genes chosen for validation were similar between the qRT-PCR and RNA-seq analyses (Fig. 5b and 7; Additional file 17, 18, 19 and 20: Figures S12–15). For example, seven unigenes (encoding SAD, FAD2, FAD3, PDAT, OLE, CLE, and SLE), which exhibited bell-shaped patterns of expression during kernel development in the RNA-seq data, were observed to exhibit similar expression patterns in the qRT-PCR data. Importantly, both RNA-seq and qRT-PCR indicated that these seven unigenes were only expressed at low levels throughout the development of the testa and pericarp. However, the qRT-PCR data indicated that FAD3 expression in the testa increased sharply at 63 DAF (Fig. 7b). RNA-seq and qRT-PCR analyses showed that the remaining six unigenes were upregulated in the kernel but expressed at low levels throughout the development of the pericarp and testa.

## Discussion

The patterns of FA accumulation differed noticeably among the pericarp, testa, and kernel. Throughout development, FA content in the pericarp was generally low, as was FA content in the testa. In contrast, concentrations of UFAs, particularly ALA, increased rapidly in the testa beginning at 63 DAF, peaking at 70 DAF. At 70 DAF, ALA content in the testa was 10-fold that of ALA content at 56 DAF; this coincided with the maximum thickening of the testa (Fig. 1d). This is interesting because the concentrations of UFAs, especially ALA, exhibited an inverted V-shaped pattern of change during testa development. To our knowledge, this is the first demonstration of changes in ALA content during testa development. The kernel began to accumulate oil rapidly once the external shape and size of the seed had stabilized. The concentrations of UFAs, especially ALA, followed a bell-like curve as the kernel matured. ALA concentration peaked at 77 DAF; at this point, the cotyledon embryo had formed completely, the seed had reached maximum size, and the pericarp was yellow. In contrast, Li et al. [10] found that ALA content was greatest at the seed-browning stage. Our results showed ALA concentrations in the *P. ostii* were high in the endosperm, moderate in the testa, and low in the pericarp. As these three tissues have different ALA concentrations, they represent good research systems for comparative studies of the mechanisms of ALA synthesis and accumulation in *P. ostii*.

Transcriptomes have previously been used to study the mechanisms underlying ALA synthesis in tree peony seeds [10–14]. Here, we identified genes associated with the lipid metabolism that were differentially expressed during the development of the seed kernel, testa, and pericarp. Although the FA metabolism is the primary metabolic process in plant cells, the pathways associated with FA biosynthesis and metabolism were not significantly enriched during pericarp and testa development. The metabolic pathways that were most significantly enriched in the pericarp and testa DEGs (e.g., fatty acid elongation, glycerolipid metabolism, cutin/suberine and wax biosynthesis, and sphingolipid metabolism) were primarily associated with the wax and cutin biosynthesis that is required for the development of the pericarp and the testa. However, at stage 63 DAF in the testa (and not at any other stages), ALA content increased sharply, and the genes encoding SAD, FAD2, and FAD3 were strongly upregulated (Fig. 7). In addition, the ALA biosynthesis and fatty acid biosynthesis pathways were significantly enriched at this stage in the testa. The inverted V-shape of the change in ALA content in the testa at 56–84 DAF was significantly correlated with the increase in ALA and FA biosynthesis. More upregulated genes than downregulated genes were identified in the kernel at 49 DAF, possibly because oil rapidly accumulates in the kernel during this period. Consistent with this, some of the genes upregulated in the kernel at 49 DAF were significantly enriched in lipid metabolism pathways, especially FA biosynthesis and metabolism. The genes significantly upregulated at 49 DAF in the kernel included those encoding α-PDH, BCCP, BC, FATA, KAS I–III, PLD, PDCT, and LPAAT. Most of these genes are known to play major roles in de novo FA biosynthesis. Genes associated with the pyruvate metabolism and with plant hormone signal transduction also tended to be upregulated, rather than downregulated, at 49 DAF in the kernel. These two pathways are also related to the FA metabolism: pyruvate metabolism is the precursor of acetyl CoA production, while plant hormone signaling pathways may activate a variety of TFs to regulate the FA metabolism [16]. Thus, the rapid synthesis and accumulation of FAs at 49 DAF in the kernel might be primarily due to the activation of the FA synthesis pathway and the upregulation of key genes.

Two types of ACCs, heteromeric ACC (HeACC) and HmACC, were expressed during the development of the *P. ostii* tissues. HeACC forms a multisubunit enzyme with BC, BCCP, alpha-CT, and beta-CT [25]. The genes encoding these subunits were differentially expressed among the three tissues throughout development. The genes encoding HmACC were highly expressed during testa development but expressed at low levels in the other two tissues. Specifically, ACC in plant plastids is heterogeneous, while ACC in plant cytoplasm is homogenous; ACC in the cytoplasm catalyzes malonyl monoacyl CoA to produce long-chain FAs, flavonoids, anthocyanins, and other secondary metabolites [26, 27]. Thus, we speculated that the step of de novo FA biosynthesis catalyzed by ACCs is specifically catalyzed by HmACC in the testa and by HeACC in the kernel. However, in the Gramineae, the ACC in the plastids is homogenous [28]. The genes encoding KAS I, KAS II, and KAS III, which are important catalysts of FA synthesis, were significantly upregulated at 49 DAF in the kernel (Additional file 13: Figure S9), while the genes encoding SAD (CL2824.Contig14 and CL2824.Contig25) exhibited a bell-shaped expression pattern. These genes were not similarly upregulated in the testa and the pericarp. The upregulation of the SAD and KAS genes during kernel development might be related to the rapid production of 18:1-ACP in this tissue. Consistent with this, the upregulation of the gene encoding FATA in the kernel, particularly at 49 DAF, might ensure that 18:1-ACP is produced rapidly via SAD catalysis and transported to the ER for downstream reactions. Thus, the rapid upregulation of KAS I, KAS III, KAS III, SAD, and FATA, as well as BCCP and BC, at 49 DAF might be required to ensure that sufficient C18:1-dominated fatty acyl flux are transported to the ER for acyl editing or TAG assembly.

ω-3 FADs are located in the plastid or the ER and are encoded by a multigene family [20]. ω-3 FADs act on C18:2 to produce C18:3 (or on C16:2 to produce C16:3). There are two types of ω-3 FADs: microsomatic FAD3, which is located in the ER and acts on PC or other phospholipids with cytochrome b as the electronic donor, and plastid FAD7/8, which is located in the plastid and acts on phosphatidylglycerol and galactose lipids with ferritin as the electronic donor [16, 20]. In *A. thaliana,* FAD7/8 were shown to be plastid isozymes of FAD3; FAD3, FAD7, and FAD8 were all present as single copies in *A. thaliana* [29–31]. Here, based on gene annotation, we identified five unigenes (unigene21286, unigene2598, unigene43483, CL1023.Contig1, and CL1023.Contig4) as encoding the temperature-sensitive ω-3 FAD7/8 (Additional file 11: Table S3). However, our phylogenetic analysis suggested that four microsomal FAD3 genes and one plastid FAD7/8 gene were expressed in *P*. *ostii*. One of the genes encoding a microsomal FAD3 (CL1023.Contig4) was most highly expressed at 77 DAF in the kernel (Fig. 7). One gene encoding a plastid FAD7/8 (Unigene21286) was expressed during kernel development at a low level. ALA content in the kernel also peaked at 77 DAF, suggesting that FAD3 might play a vital role in the formation of ALA. In the testa, CL1023.Contig4 was also highly expressed at 63 DAF, followed by in a sharp increase in ALA content at 70 DAF. In contrast, FAD3 was not highly expressed in the pericarp, and ALA content in the pericarp was very low. Although a previous study concluded that the high concentrations of ALA in *P. ostii* seeds were due to FAD8 activity and abundance [10], our results suggested that high concentrations of ALA in the kernel might be associated with the activity and abundance of FAD3, not FAD8. Other plants with high concentrations of ALA, such as flax, perilla, and sea-buckthorn, invariably express both FAD3 and FAD7/8 [32–35]. For example, the upregulation of FAD3 in flax was associated with high ALA content during seed development [32, 33]. In *Perilla frutescens,* FAD3 was more highly expressed than FAD7/8 during seed development, but FAD7/8 was more highly expressed than FAD3 in the leaves [34]. In the fruits of the sea-buckthorn (*Hippophae rhamnoides*) levels of FAD3 and FAD7/8 expression were similarly upregulated, leading to high levels of ALA [35]. However, even among species with high ALA content, the enzymes that play key roles in ALA synthesis differ, suggesting that ALA synthesis pathways also differ among plants, or even among tissues.

De novo FA synthesis, and the subsequent acyl flux through the Kennedy pathway, produces oils containing saturated and monounsaturated FAs [16]. However, the production of more diverse TAG acyl compositions in plants requires the flux of acyl groups through the membrane lipids PC or PE [16, 21]. Therefore, the accumulation of TAG assemblies containing various PC/PE-modified FAs requires the coordinated flux of acyl groups into PC/PE for desaturation, and the subsequent flux of acyl groups out of the PC/PE and into TAG assemblies (Fig. 5a). In conjunction, acyl editing and the Lands cycle regulate acyl flux, to ensure that acyl groups are modified to increase the diversity of TAG acyl compositions [21]. In the three *P. ostii* tissues, we detected 28 unigenes associated with acyl editing and the Lands cycle, including three CPT-encoding unigenes, one PDCT-encoding unigene, three LPCAT-encoding unigenes, two LPEAT-encoding unigenes, and 19 PLD-encoding unigenes. In particular, one PDCT-encoding unigene (unigene32372) and one PLD-encoding unigene (CL1337.Contig6) showed bell-shaped expression patterns during kernel development, but were expressed minimally in the pericarp and testa (Additional file 13: Figure S9). This indicated that, compared with the testa and pericarp, the high expression of these genes in kernel was very important for overall carbon flux into TAG in *P. ostii* in the form of PUFAs. To our knowledge, unigene32372 and CL1337.Contig6 have not previously been identified as key genes in tree peony seed development.

In *A. thaliana,* the reduced oleate desaturase 1 gene (*rod1*) was shown to encode a novel enzyme (PDCT); when *rod1* expression was inhibited, the accumulation of PC-modified fatty acids in the TAG assembly decreased ~ 40%, although overall levels of TAG remained constant [36]. As PC/PE is the site for extra-plastidial FA modification, the flux of the de novo DAG moiety through PC can provide a PC-derived DAG pool with a different acyl composition than that of de novo DAG (Fig. 5a). However, the relative proportion of TAG synthesized from the Kennedy pathway using de novo DAG to TAG synthesized from PC-derived DAG is unclear in most plants. Indeed, the PC-derived DAG pool, rather than the de novo DAG pool, might be the primary source of DAG for TAG synthesis in various plants [36, 37]. The DGAT enzyme has also been implicated in the formation of TAG from de novo DAG and acyl-CoA (the final step of the Kennedy pathway; Fig. 5a, blue line). For example, DGAT1, DGAT2, and DGAT3 drive TAG production in *Arabidopsis* [38], peanuts [39], and tung trees [40]. Here, although four DGAT-encoding unigenes were identified in *P. ostii*, only one unigene (CL5087.Contig1) was strongly upregulated at 49 DAF in the testa, weakly upregulated at same stage in the kernel, and expressed minimally throughout development in the pericarp (Additional file 19: Figure S14). TAG synthesis may also be catalyzed by PDAT, using FAs from the PC pool and PC-derived DAG as the substrate (Fig. 5a, green line). Here, the PDAT-encoding unigene (unigene24505) showed a bell-shaped expression pattern in the developing kernel, but was minimally expressed in the testa and the pericarp (Additional file 19: Figure S14). This indicated that in the *P. ostii* kernel, PDAT reflected the amount of PFUA-containing TAG more accurately than DGAT. Thus, our results indicated that the PDAT pathway (Fig. 5a, green line), rather than the Kennedy pathway (Fig. 5a, blue line), might be the primary mechanism of TAG generation in the *P. ostii* kernel. Zhang et al. [13] reported that PDAT levels were significantly associated with TAG accumulation in *P. rokii*. However, a previous study concluded that the PDAT pathway was probably not an important pathway for TAG generation in tree peony seeds, due to the low abundance of PDAT-encoding gene transcripts [10].

In mature seeds, TAGs can be stored as oil bodies, surrounded by a phospholipid monolayer and abundant amphipathic proteins (e.g., OLE, CLE, and SLE) [41–43]. Throughout development, the expression levels of the gene homologs encoding OLE, CLE, and SLE either increased or were consistently high in the kernel and the testa, ensuring efficient ALA accumulation, but remained low in the pericarp (Additional file 20: Figure S15). In all three tissues, the CLE-encoding genes were more strongly upregulated than the OLE- or SLE-encoding genes. However, during the development of *Brassica napus* seeds and *Prunus sibirica* kernels, OLE was much more highly expressed than CLE and SLE [41, 44]. This suggested that mechanisms of oil formation differ among plants.

TFs, such as WRI1, FUS3, ABI3, bZIP, LEC1, LEC2, MYB, and GL2, have previously been shown to play key roles in seed oil synthesis and deposition [45]. Here, transcriptional profiling revealed that WRI1 and FUSCA participated in the positive regulation of genes associated with oil synthesis during kernel development (Fig. 7b). Notably, the AP2 TF WRI1 is upregulated by LEC1, LEC2, ABI3, and FUS3 in *Arabidopsis* [45]. During kernel development, both FUS3 and WRI1 were upregulated (Fig. 6b), suggesting that the gene encoding WRI1 might be a direct target of FUS3 in the developing kernel. Additionally, the co-upregulation of plastidial ACC (BCCP and BC), KAS I–III, SAD, and FATA with WRI1 during kernel development (Additional file 13: Figure S9) suggested that WRI1 might be involved in the transcriptional regulation of these target genes, as was shown in *Arabidopsis* seeds. Thus, WRI1 (CL22311) might play a critical role in the regulatory network controlling ALA accumulation during kernel development. An important regulator of gene transcription in the soybean is zinc finger CCCH domain containing protein (GmZF351). Transgenic experiments using *Arabidopsis* showed that GmZF351 directly regulated WRI1, BCCP, KAS III, OLE and other genes; in addition, GmZF351 overexpression in soybeans increased oil content [46]. Here, we found that the expression pattern of unigene32844, which encoded the TF zinc finger CCCH domain-containing protein 54 (C3H54), in the seed kernel were consistent with the changes in ALA content (Fig. 2 and 6b). Therefore, this TF might be a useful research target for future studies of the regulation of ALA biosynthesis.

## Conclusions

Our results indicated that the optimal time for tree peony oil collection was 77 DAF, when the fruit peel was yellow and oil quantity and quality were greatest. That is, FA concentration increased dramatically during cotyledon embryogenesis, and continued to increase steadily to peak at 77 DAF. This rapid increase in seed oil content was associated with the differential expression of many genes associated with FA and TAG synthesis during embryogenesis. Many FA biosynthesis and metabolism pathways were significantly enriched in these DEGs in the kernel at the early stages of rapid oil accumulation. ALA content also increased sharply in the testa during development; many of the genes differentially expressed in the testa at this stage were significantly enriched in ALA and FA biosynthesis. In total, we identified 1373 unigenes related to lipid metabolism in *P. ostii*. Of these, unigenes with bell-shaped expression patterns (e.g., those encoding SAD, FAD2, FAD3, PDCT, PDAT, OLE, CLE, and SLE) and unigenes that were upregulated at 49 DAF (e.g., those encoding BCCP, BC, KAS I–III, and FATA) may be useful targets for future investigations of lipid metabolism in the tree peony. Similarly, many regulatory enzymes (e.g., PDH, ACC, KAS I–III, SAD, FATA, FAD2, FAD3 LPCAT/LPEAT, CPT, PDCT, DGAT, PDAT, OLE, CLE, and SLE) and TFs (WRI1 and FUS3), which are crucial for the biosynthesis of acetyl-CoA, FA, TAG, and the oil body, and which were significantly enriched during kernel development, deserve further investigation. In this study, we used three tissues with high, moderate, and low ALA concentrations as an exemplar system in which to compare tissue-specific ALA accumulation mechanism in *P. ostii*. We found that key genes related to ALA synthesis were differentially expressed among the three tissues. Thus, our results help to provide a framework for future studies of the tree peony, particularly those aimed at improving tree peony seed oil production through breeding, genetic diversification, and gene excavation.

## Methods

### Plant materials

Fruits and seeds of *P. ostii* were collected in 2016 and 2017 at Shanghai Chenshan Botanical Garden (31°4′52″N, 121°10′14″E), Shanghai, China. These *P. ostii* were introduced to the garden more than 10 years ago, and have been grown under the same environmental and cultivation conditions since that time. Now, there are thousands of living plants of *P. ostii* growing in Shanghai Chenshan Botanical Garden. We observed the development of the fruits and seeds produced by these trees over two growing seasons (April–August, 2016 and 2017). Each year, the budded flowers of both selected plants (CS0016 and CS0009) were hand-pollinated with the pollen collected from a third *P. ostii* tree (CS0010). The pollination date was recorded as 0 DAF. Between 0 and 119 DAF, seeds were collected every 7 days from both trees, for a total of 17 samples. After seed collection, the pericarp, testa, and kernel were separated manually, frozen in liquid nitrogen, and stored at − 80 °C. Separated samples were used for FA detection, transcriptome sequencing, and qRT-PCR. In addition, the voucher specimens of *P. ostii* was identified by Yonghong Hu and deposited in Shanghai Chenshan Herbarium numbered CSH0184197.

### Measurement of FA accumulation

To explore how FA composition changed in the pericarp, testa, and kernel throughout development, we measured the FA concentrations in the pericarp, testa, and kernel samples taken from both trees between 21 and 119 DAF (a total of 42 samples per tree per year). The samples used for FA analysis were dried at 60 °C to a constant weight. Total lipids were extracted from the dried biomass as previously described [47], and FAs were methylated as previously described in spinach [48], with minor modifications. First, the dried, powdered sample was added to 3 mL of a 1:2 chloroform-methanol (v/v) mixture. Next, this solution was incubated in a water bath at 35 °C for 1 h at 120 rpm for lipid extraction. After the full extraction, 1.0 mL supplementary chloroform was added to the solution, and the mixture was vortexed. Then, 1.8 mL ddH_2_O was added, to generate a solution with a final chloroform:methanol:ddH_2_O ratio of 1:1:0.9 (v/v). The solution was then centrifuged at 4000 g for 15 min. The chloroform layer was withdrawn, and then dried with sample concentrators under a nitrogen evaporator. The concentrated lipids were then re-dissolved in 2 mL of an H_2_SO_4_-methanol solution (2% H_2_SO_4_). After charging with nitrogen gas, the solution was vortexed for 1 min, and then incubated in a 90 °C water bath for 1 h. After incubation, 1 mL ddH_2_O and 1 mL hexane were added to the solution. The solution was vortexed, and then centrifuged at 4000 g for 15 min. The supernatant was transferred to a new tube, concentrated using bubbling nitrogen, and stored at 4 °C for gas chromatograph-mass spectrometry (GC-MS) analysis. We used 50 mg/mL nonadecanoic acid in hexane as the internal standard. The FA methyl esters were measured using a GC-MS (GC7890/MS5975, Agilent) on a HP-88 capillary column (60 m long × 0.25 mm internal diameter; 0.2 μm; Agilent). FA methylation was tested and analyzed following Yu et al.^6^

### RNA extraction and cDNA library construction

As preliminary results indicated that patterns of FA accumulation differed substantially among the three tissues, we sequenced the transcriptomes of the kernel, testa, and pericarp throughout development to explore the molecular mechanisms underlying FA accumulation in the three tissues. RNA was extracted from kernel, testa, and pericarp samples taken at 35, 49, 63, 77, 91, and 119 DAF from CS0016 in 2016 and 2017, and CS0009 in 2017. Total RNA was extracted from these 54 samples using RNA Exaction Kits (E.Z.N.A. HP Plant RNA Kit, Omega Bio-Tek), and then purified using the RNeasy Plant Mini Kit (Qiagen), following the manufacturer’s protocols. The concentration and quality of each RNA sample was determined using an Agilent 2100 Bioanalyzer (Agilent Technologies). All samples had an OD260/OD280 ratio of 2.0–2.1, and an RNA integrity number > 7.0. Approximately 2 μg of total RNA was used for library construction with the Illumina TruSeq Stranded mRNA Library Prep Kit (San Diego, CA, USA). Short fragments were purified and resolved with EB buffer for end repair. Single A (adenine) nucleotides were then added. Next, the short fragments were connected with adapters, and suitable fragments were selected for PCR amplification. The Agilent 2100 Bioanalyzer and the ABI StepOnePlus Real-Time PCR System were used for the quality control of the sample library. The generated cDNA libraries were sequenced at BGI-Shenzhen, using an Illumina HiSeq 4000 system, yielding 151-bp paired-end reads.

### Unigene assembly and annotation

Raw sequencing reads were filtered to get clean reads by using SOAPnuke (v1.5.2) [49]. The raw reads were preprocessed to remove clipped adapter sequences, low-quality reads (Q value ≤20 or containing ambiguous nucleotides), and contaminated sequences. The clean reads were de novo assembled using Trinity (v2.0.6) [50], with the following parameters: “--group_pairs_distance 330 --no_version_check --full_cleanup --verbose --min_contig_length 150 --CPU 8 --min_kmer_cov 3 --min_glue 3 --bfly_opts ‘-V 5 --edge-thr=0.1 --stderr’”. Redundant sequences were removed, and remaining sequences were clustered based on homology. Based on overlap, the fragments were merged or extended into longer transcripts to form a set of non-redundant unigenes. To annotate the obtained unigenes, we used BLASTX (e-value < 0.00001) to search several public databases in the following order: NCBI non-redundant (Nr), Swiss-Prot, KEGG, and COG/KOG. The Gene Ontology (GO) [51] annotation for unigenes was obtained using Blast2GO [52]. GO classification and enrichment analyses were performed using WEGO [53].

### Identification of DEGs

We mapped the clean reads to unigenes using Bowtie2 (v2.2.5) [54], and then calculated gene expression level with RSEM (v1.2.12) [55]. Unigene expression levels were calculated and normalized using the Fragments Per Kilobase per Million mapped fragments (FPKM) method. We then used several methods to cluster the gene expression data; clustering genes based on expression patterns helps to identify genes with similar functions, which may be associated with the same biological functions. We performed principal components analysis (PCA) of all samples using the princomp function in R (v3.2.0). We used Cluster (v3.0) [56] and the Euclidean matrix formula to cluster the expressed genes and sample schemes simultaneously. The resulting clusters were visualized using Java Treeview. We also used the heatmap function in R to draw a complete clustering graph. We used Mfuzz (v2.34.0), which employs a loose clustering algorithm, to analyze the time series data. EBseq, which is based on an empirical Bayesian model, was used to identify DEGs following Leng et al. [57]. We considered genes with a Posterior Probability of being Equivalent Expression (PPEE) ≥ 0.05 and a fold-change in relative expression ≥2 significantly differentially expressed. We next classified the identified DEGs based on GO and KEGG annotations. We calculated GO functional enrichment and KEGG pathway enrichment using phyper in R (v3.2.0).

### Analysis of unigene expression patterns and associated TFs

All protein sequences were downloaded from Swiss-Prot. Based on the functional annotations of the DEGs, we selected all DEGs associated with the lipid metabolism. To better understand the molecular factors underlying ALA accumulation during kernel development, we focused on the DEGs associated with FA and TAG biosynthesis.

The sequence of three ω-3 FADs from *Arabidopsis thaliana* (AtFAD3_AT2G29980, AtFAD7_AT3G11170, and AtFAD8_AT5G05580) were downloaded from the NCBI. We aligned the *A. thaliana* ω-3 FADs with those from *P. ostii* (unigene21286, unigene2598, unigene43483, CL1023.Contig1, and CL1023.Contig4) using clustalX [58]. We then constructed a phylogenetic tree based on this multiple sequence alignment using FastTree2 [59].

To investigate how TFs regulated tree peony oil biosynthesis, we analyzed all of the DEGs that were also TFs. First, we used Getorf to detect the ORF of each DEG. These ORFs were then mapped to the transcription factor protein domain database (from PlntfDB) using Hmmsearch [60]. We predicted the TF coding ability of each DEG based on the characteristics of each TF family. Based on the Swiss-Prot annotations of the DEGs, we identified the TF DEGs related to FA metabolism.

### qRT-PCR validation of the expression patterns of key DEGs involved in oil accumulation

To experimentally validate our RNA-seq data, we selected 13 unigenes associated with FA biosynthesis and TAG assembly, in particular those related to ALA, for qRT-PCR validation. The unigenes selected were CL2824.Contig25, encoding SAD; CL15521.Contig3, encoding FAD2; CL1023.Contig4, encoding FAD3; CL5945.Contig8, encoding FAD6; Unigene21286, encoding FAD7/8; CL5087.Contig1, encoding DGAT; Unigene24505, encoding PDAT; Unigene5123, encoding OLE; CL18131.Contig3, encoding CLE; and Unigene19272, encoding SLE. The expression levels of these unigenes were quantified using qRT-PCR in the kernel, testa and pericarp at 35, 49, 63, 77, 91, and 119 DAF (18 samples in total). Total RNA was isolated from each tissue at each time point using RNA Exaction Kits (E.Z.N.A. HP Plant RNA Kit, Omega Bio-Tek). First-strand cDNA was prepared from 1 μg of total RNA per sample using a FastKing RT Kit with gDNase (Tiangen). Specific primers were designed for each of the 13 unigenes (Additional file 21: Table S6). PCRs were performed on an ABI StepOnePlus Real-Time PCR System (Applied Biosystems), following the manufacturer’s instructions. Each 20 μL reaction mixture contained 10 μl of TB Green Premix Ex Taq II (Tli RNaseH Plus) (Takara), 0.8 μl of each primer (10 μM), 0.3 μl of cDNA template (1 μg), and 8.1 μl of RNase-free water. PCRs for each gene were performed in triplicate, with the following thermal cycling conditions: 95 °C for 30 s; 40 cycles of 95 °C for 5 s and 64 °C for 30s; and 95 °C for 15 s. Primer specificity was confirmed by melting curve analysis. The relative expression levels of the tested genes were calculated using the 2^-ΔΔCt^ method [61], with the *actin* genes as internal controls.

## Supplementary Information

**Additional file 1: Table S1.** Statistics for the unigene sets assembled from *Paeonia ostii*.

**Additional file 2: Figure S1.** Heatmap showing correlations among samples. Samples are plotted along the x- and y-axes. Higher correlations are indicated by darker blues.

**Additional file 3: Figure S2.** Venn diagrams showing the numbers of expressed genes unique and shared among the seed kernel, seed testa, and fruit pericarp of *Paeonia ostii* at six different developmental stages.

**Additional file 4: Table S2.** Total number of the differentially expressed genes (DEGs) among the three *Paeonia ostii* tissues at various time points.

**Additional file 5: Figure S3.** Heatmap showing the hierarchical clustering of the differentially expressed genes. The samples compared are plotted along the x-axis; the DEGs are plotted along the y-axis. The color of each intercept represents the log2-transformed fold change value (high: red, low: blue).

**Additional file 6: Figure S4.** GO classification of differentially expressed genes. The number of DEGs is plotted along the x-axis; the GO terms is plotted along the y-axis. **a** T1 vs. T2 in the kernel. **b** T1 vs. T2 in the testa. **c** T1 vs. T2 in the pericarp.

**Additional file 7: Figure S5.** KEGG pathways enriched in the genes differentially expressed in the developing kernel at various time points. The enrichment factor is plotted on the x-axis; pathway names are shown on the y-axis. The color of each dot reflects the Qvalue, while the size of the dot represents the number of DEGs. **a** T1 vs. T3. **b** T1 vs. T4. **c** T1 vs. T5. **d** T1 vs. T6.

**Additional file 8: Figure S6.** KEGG pathways enriched in the genes differentially expressed in the developing testa at various time points. The enrichment factor is plotted on the x-axis; pathway names are shown on the y-axis. The color of each dot reflects the Qvalue, while the size of the dot represents the number of DEGs. **a** T1 vs. T4. **b** T1 vs. T5. **c** T1 vs. T6.

**Additional file 9: Figure S7.** KEGG pathways enriched in the genes differentially expressed in the developing pericarp at various time points. **a** The number of genes associated with various KEGG pathways differentially expressed between T1 and T2. **b** The KEGG pathways overrepresented in the genes differentially expressed between T1 and T2. The color of each dot reflects the Qvalue, while the size of the dot represents the number of DEGs. **c** The up- and downregulated genes in T2 as compared to T1 that were associated with each KEGG pathway.

**Additional file 10: Figure S8.** KEGG pathways enriched in the genes differentially expressed in the developing pericarp at various time points. The enrichment factor is plotted on the x-axis; pathway names are shown on the y-axis. The color of each dot reflects the Qvalue, while the size of the dot represents the number of DEGs. **a** T1 vs. T3. **b** T1 vs. T4. **c** T1 vs. T5. **d** T1 vs. T6.

**Additional file 11: Table S3.** The 1373 unigenes related to lipid metabolism identified in this study, annotated using Nr, Nt, Swiss-Prot, KEGG, and GO.

**Additional file 12: Table S4.** The relative expression (in FPKM) of *Paeonia ostii* genes associated with 10 lipid metabolism pathways.

**Additional file 13: Figure S9.** Heat map showing the expression patterns of the unigenes associated with lipid metabolism during the development of the seed kernel (k), seed testa (t), and fruit pericarp (p) of *Paeonia ostii*. Labels on the y-axis indicate which plant was used (CS0009 or CS0016), the tissue (k, t, or p), and the developmental period (35–119 days after fertilization, DAF).

**Additional file 14: Figure S10.** Phylogenetic relationships among ω-3 FADs from *Paeonia ostii* and *Arabidopsis thaliana*.

**Additional file 15: Table S5.** Annotations and FPKM values for the 125 transcription factors associated with FA biosynthesis during the development of the three *Paeonia ostii* tissues.

**Additional file 16: Figure S11.** The relative expression levels of genes associated with key TFs. **a** Kernel. **b** Testa. **c** Pericarp.

**Additional file 17: Figure S12.** Quantitative real-time PCR validation of ACCase genes. **a** Kernel. **b** Testa. **c** Pericarp.

**Additional file 18: Figure S13.** Quantitative real-time PCR validation of FAD genes. **a** Kernel. **b** Testa. **c** Pericarp.

**Additional file 19: Figure S14.** Quantitative real-time PCR validation of PDAT and DGAT genes. **a** Kernel. **b** Testa. **c** Pericarp.

**Additional file 20: Figure S15.** Quantitative real-time PCR validation of oil-associated genes. **a** Kernel. **b** Testa. **c** Pericarp.

**Additional file 21: Table S6.** Primer sequences used for quantitative real-time PCR.

## Data Availability

The Illumina read data used for expression profiling of the *Paeonia ostii* reference genes have been submitted to the NCBI Sequence Read Archive (SRA) under the accession number PRJNA595001. All other data supporting our findings can be found in Additional files 1–21.

## Abbreviations

ACC: Acetyl-CoA carboxylase
ACP: Acyl carrier protein
ALA: Alpha linolenic acid
BC: Biotin carboxylase
BCCP: Biotin carboxyl carrier protein
CT: Carboxyltransferase
CLE: Caleosins
CPT: CDP-choline: DAG choline phosphotransferase
DAG: Diacylglycerol
DAF: Days after fertilization
DEG: Differentially expressed gene
DGAT: Diacylglycerol acyltransferase
EPT: CDP-ethanolamine: DAG ethanolamine phosphotransferase
DAG: Ethanolamine phosphotransferase
ER: Endoplasmic reticulum
FA: Fatty acid
FAS: Fatty acid synthase
FATA/B: Acyl-ACP thioesterase A/B
FAD2: Oleate desaturase
FAD3: Linoleate desaturase
FPKM: Fragments per kilobase per million mapped fragments
G3P: Glycerol-3-phosphate
GPAT: Glycerol-3-phosphate acyltransferase
KAS I, II, III: ketoacyl-ACP synthase I, II, III
KEGG: Kyoto encyclopedia of genes and genomes
LA: Linoleic acid
LACS: Long-chain acyl-CoA synthetase
LEC1: Leafy cotyledon1
LPA: Lysophosphatidic acid
LPAAT: Lysophosphatidyl acyltransferase
LPC: Lysophosphatidylcholine
LPE: Lysophosphatidylethanolamine
LPCAT: Lysophosphatidylcholineacyltransferase
LPEAT: Lysophosphatidylethanolamineacyltransferase
MCMT: Malonyl-CoA ACP transacylase
OA: Oleic acid
OLE: Oleosin
PA: Phosphatidic acid
PAP: Phosphatidic acid phosphohydrolase
PC: Phosphatidylcholine
PE: Phosphatidylethanolamine
PDAT: Phospholipid: diacylglycerol acyltransferase
PDCT: Phosphatidylcholine: diacylglycerol cholinephosphotransferase
PDH: Pyruvate dehydrogenase
PLC: Phospholipase C
PLD: Phospholipase D
PUFA: Polyunsaturated fatty acid
SAD: Stearoyl-ACP desaturase
SLE: Steroleosin
TAG: Triacylglycerol
TF: Transcription factor
UFA: Unsaturated fatty acid
