# Supplementary material for: Transcriptomic analysis of α-linolenic acid content and biosynthesis in Paeonia ostii fruits and seeds
Source: BMC Genomics. 2021 Apr 23;22:297. doi: 10.1186/s12864-021-07594-2 (PMC8063412; doi:10.1186/s12864-021-07594-2)
Supplement: Supplementary file 1 — Additional file 1: Table S1. Statistics for the unigene sets assembled from Paeonia ostii. [file 12864_2021_7594_MOESM1_ESM.pdf]

Table S1 Statistics of the unigene sets assembled from *Paeonia ostii*.

**Sample**	**Total Number**	**Total Length**	**Mean Length**	**N50**	**N70**	**N90**	**GC(%)**
kernel_35 DAF	23890	10872176	455	556	367	227	43.04
kernel_49 DAF	24258	10685457	440	535	364	225	42.38
kernel_63 DAF	30987	16020155	516	674	430	244	42.85
kernel_77 DAF	31478	14146988	449	528	336	225	43.75
kernel_91 DAF	29715	15599949	524	684	414	244	43.55
kernel_119 DAF	36831	23940168	650	943	562	279	43.19
pericarp_35 DAF	52162	31764646	608	891	520	261	43.34
pericarp_49 DAF	63327	43190334	682	1081	614	275	43.6
pericarp_63 DAF	48399	36087725	745	1156	703	308	42.65
pericarp_77 DAF	50959	38991461	765	1193	723	313	42.66
pericarp_91 DAF	42408	37107139	875	1392	866	362	42.37
pericarp_119 DAF	85229	51356372	602	930	476	249	48.04
testa_35 DAF	37415	30564961	816	1304	789	331	42.8
testa_49 DAF	45800	33328961	727	1091	664	309	42.33
testa_63 DAF	44344	37726440	850	1358	827	344	42.36
testa_77 DAF	53568	44338088	827	1326	788	334	41.84
testa_91 DAF	48876	41444384	847	1348	820	344	42
testa_119 DAF	14231	4617652	324	314	227	209	48.16
All-unigene	227837	1.79E+08	786	1386	780	286	43.54

DAF: day after fertilization
