# Supplementary material for: Transcriptomic analysis of α-linolenic acid content and biosynthesis in Paeonia ostii fruits and seeds
Source: BMC Genomics. 2021 Apr 23;22:297. doi: 10.1186/s12864-021-07594-2 (PMC8063412; doi:10.1186/s12864-021-07594-2)
Supplement: Supplementary file 2 — Additional file 2: Figure S1. Heatmap showing correlations among samples. Samples are plotted along the x- and y-axes. Higher correlations are indicated by darker blues. [file 12864_2021_7594_MOESM2_ESM.pdf]

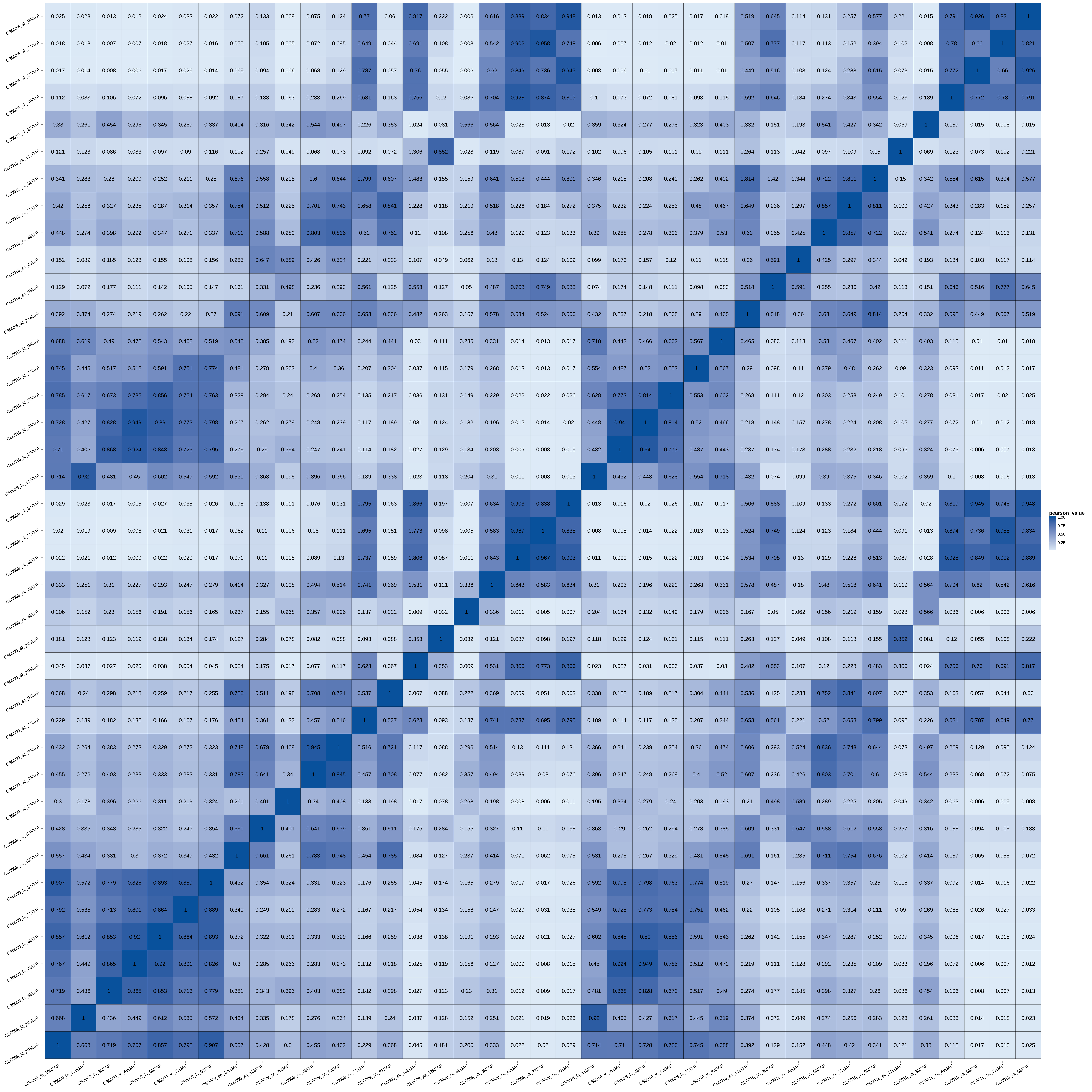
CS0016_sk_98DAF
CS0016_sk_77DAF
CS0016_sk_63DAF
CS0016_sk_49DAF
CS0016_sk_35DAF
CS0016_sk_116DAF
CS0016_sc_98DAF
CS0016_sc_77DAF
CS0016_sc_63DAF
CS0016_sc_49DAF
CS0016_sc_35DAF
CS0016_sc_116DAF
CS0016_fc_98DAF
CS0016_fc_77DAF
CS0016_fc_63DAF
CS0016_fc_49DAF
CS0016_fc_35DAF
CS0016_fc_116DAF
CS0009_sk_91DAF
CS0009_sk_77DAF
CS0009_sk_63DAF
CS0009_sk_49DAF
CS0009_sk_35DAF
CS0009_sk_129DAF
CS0009_sk_105DAF
CS0009_sc_91DAF
CS0009_sc_77DAF
CS0009_sc_63DAF
CS0009_sc_49DAF
CS0009_sc_35DAF
CS0009_sc_129DAF
CS0009_sc_105DAF
CS0009_fc_91DAF
CS0009_fc_77DAF
CS0009_fc_63DAF
CS0009_fc_49DAF
CS0009_fc_35DAF
CS0009_fc_129DAF
CS0009_fc_105DAF
pearson_value
1.00
0.75
0.50
0.25
