# Supplementary figures and images for: Transcriptomic analysis of α-linolenic acid content and biosynthesis in Paeonia ostii fruits and seeds
Source: BMC Genomics. 2021 Apr 23;22:297. doi: 10.1186/s12864-021-07594-2 (PMC8063412; doi:10.1186/s12864-021-07594-2)

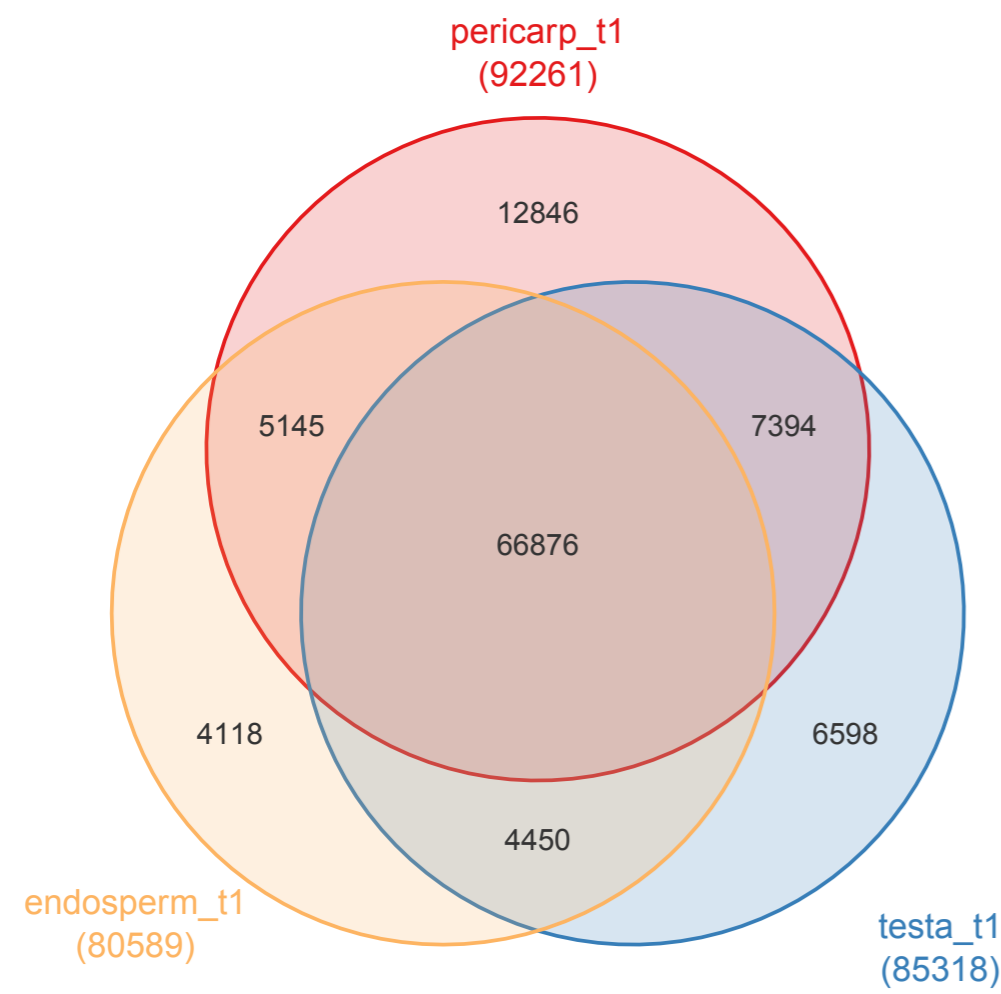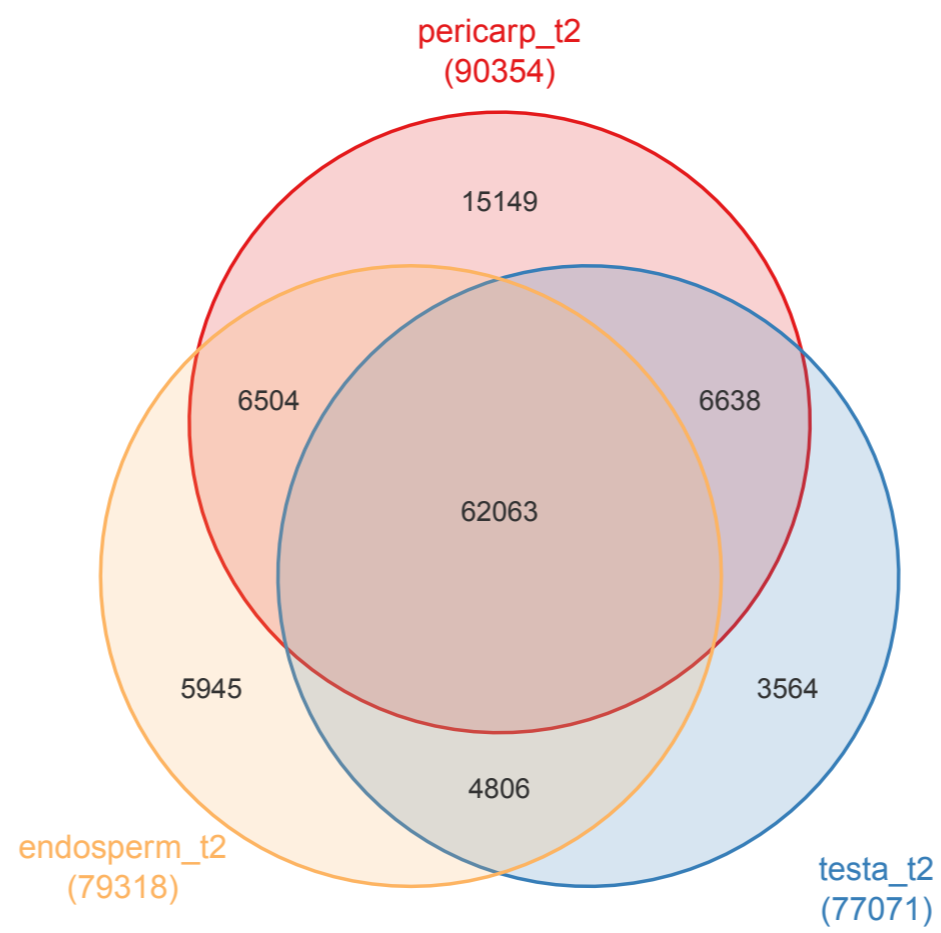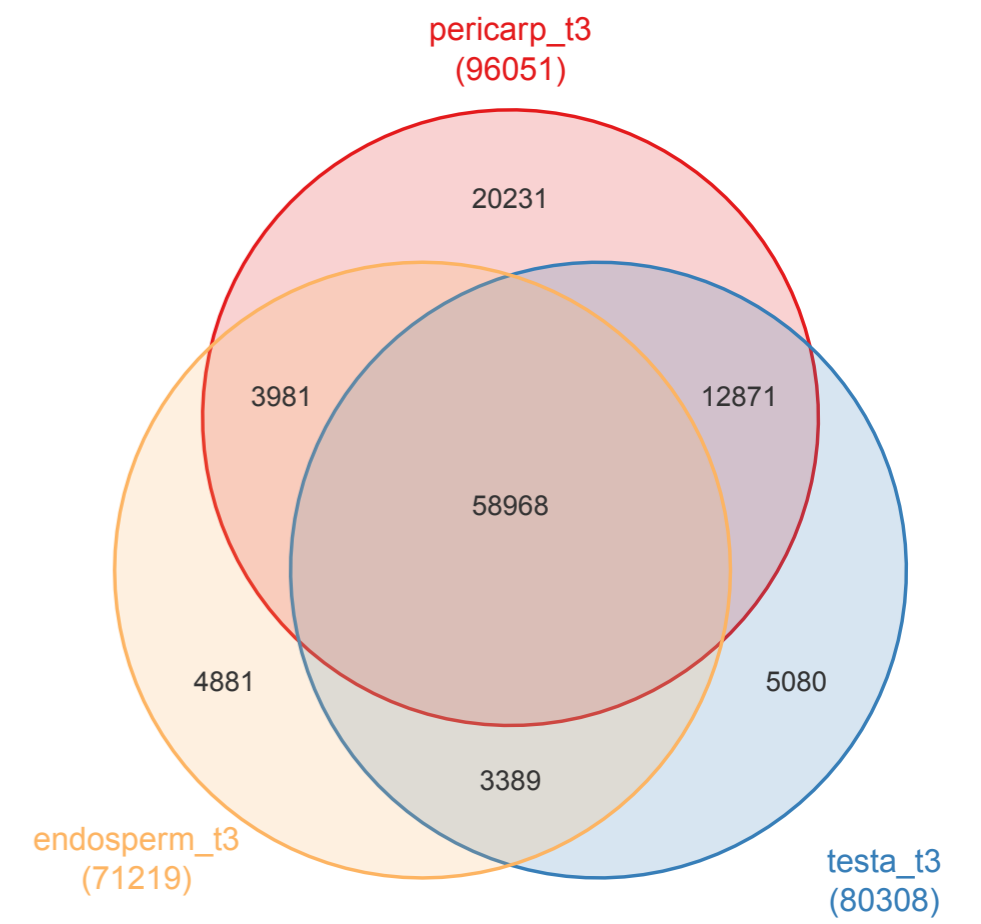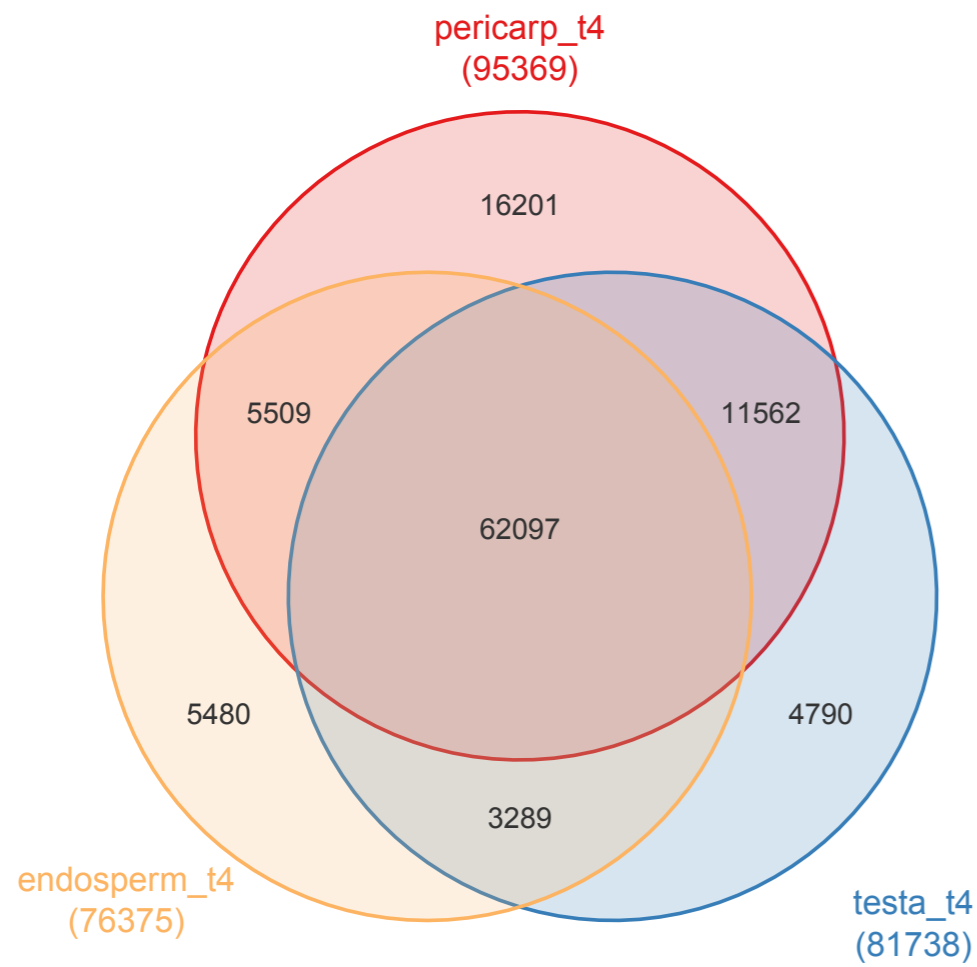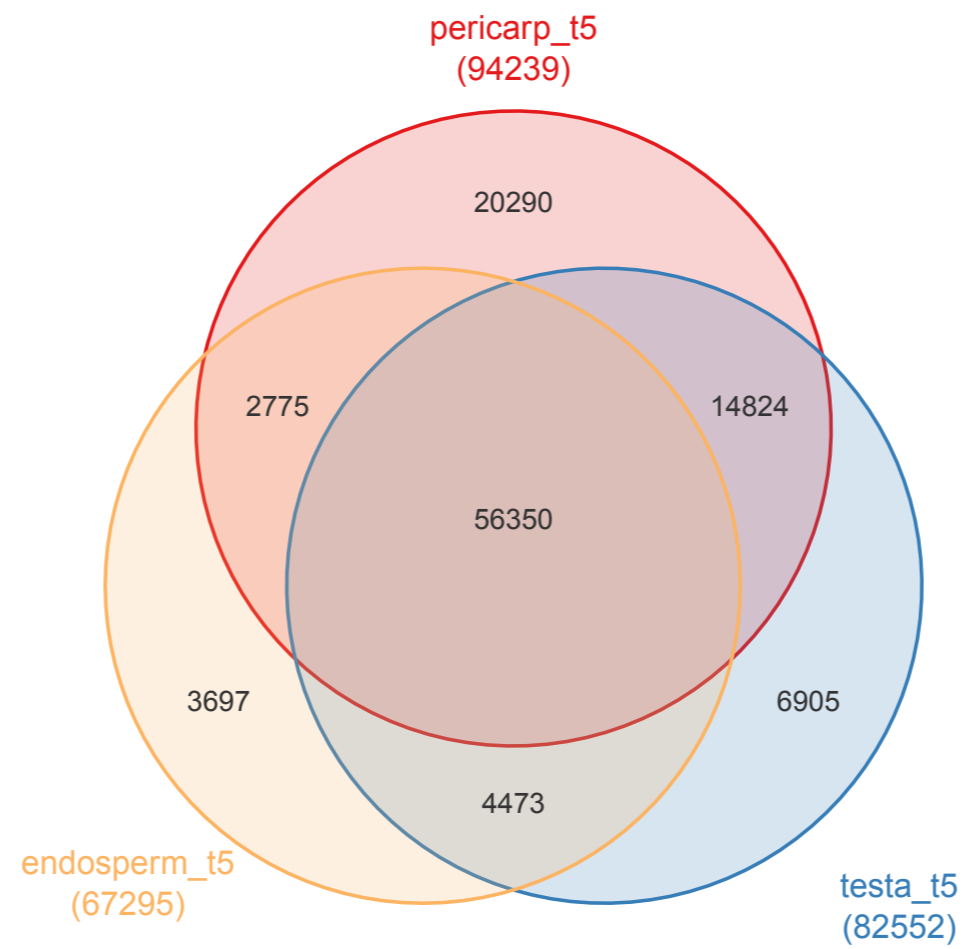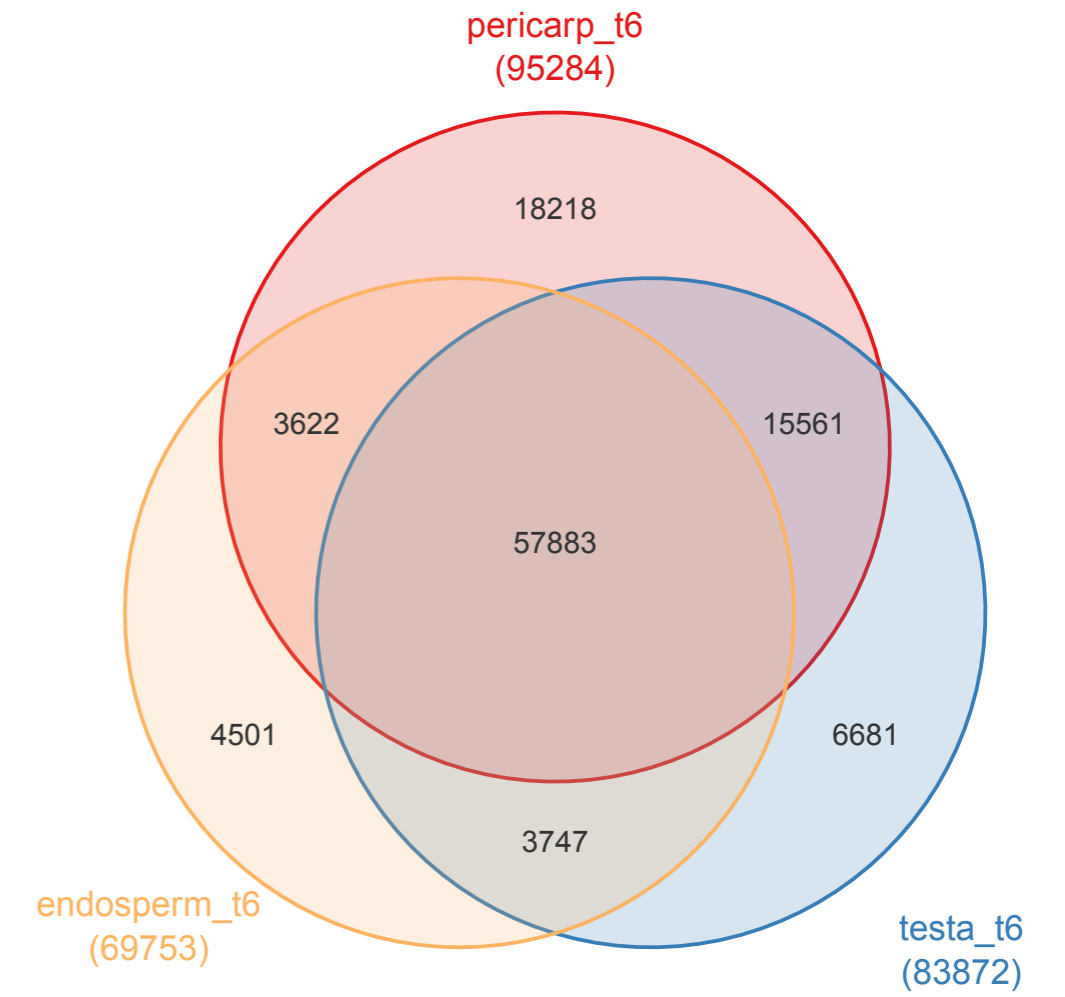

Supplement: Supplementary file 3 — Additional file 3: Figure S2. Venn diagrams showing the numbers of expressed genes unique and shared among the seed kernel, seed testa, and fruit pericarp of Paeonia ostii at six different developmental stages. [file 12864_2021_7594_MOESM3_ESM.pdf]

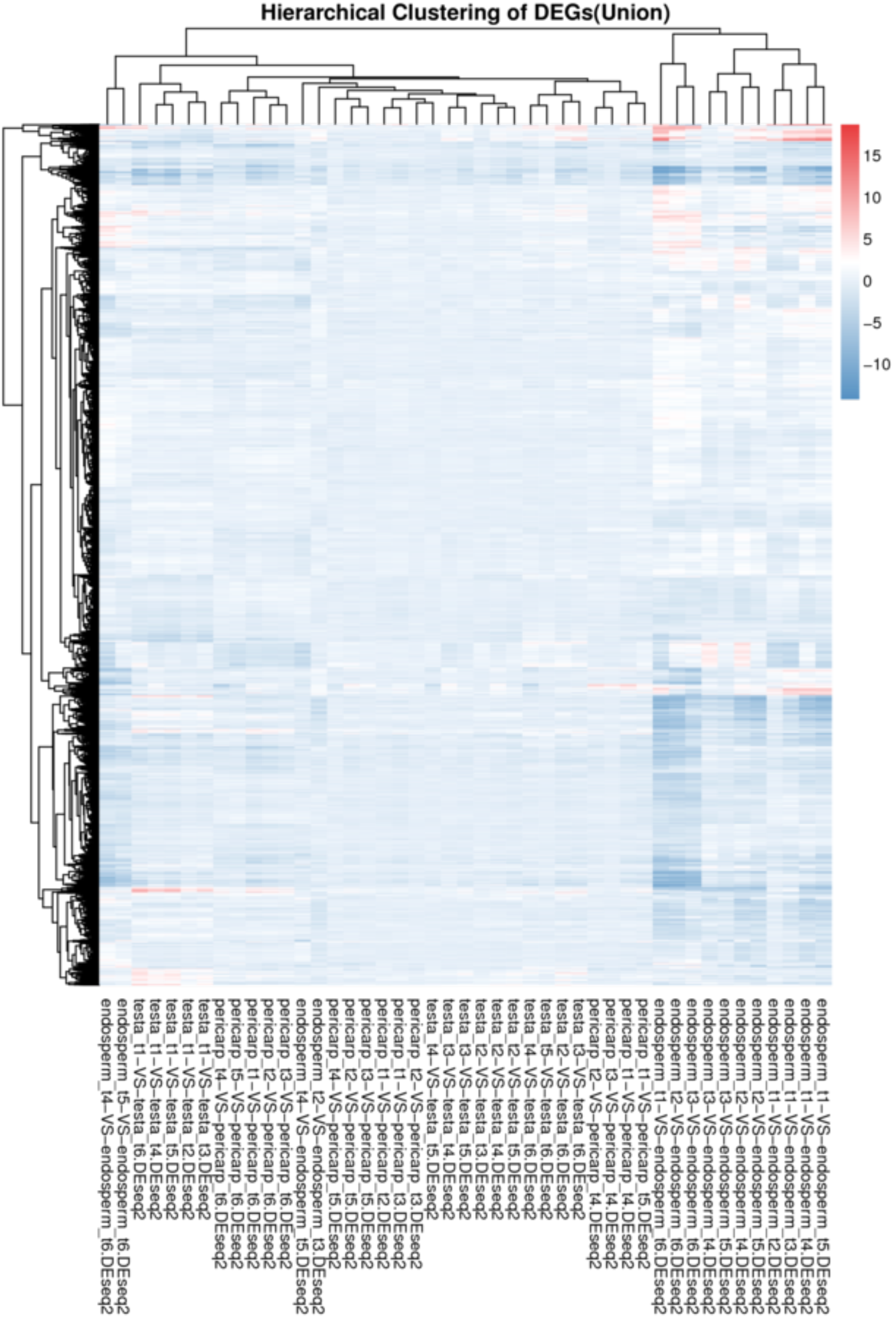

Supplement: Supplementary file 5 — Additional file 5: Figure S3. Heatmap showing the hierarchical clustering of the differentially expressed genes. The samples compared are plotted along the x-axis; the DEGs are plotted along the y-axis. The color of each intercept represents the log2-transformed fold change value (high: red, low: blue). [file 12864_2021_7594_MOESM5_ESM.pdf]

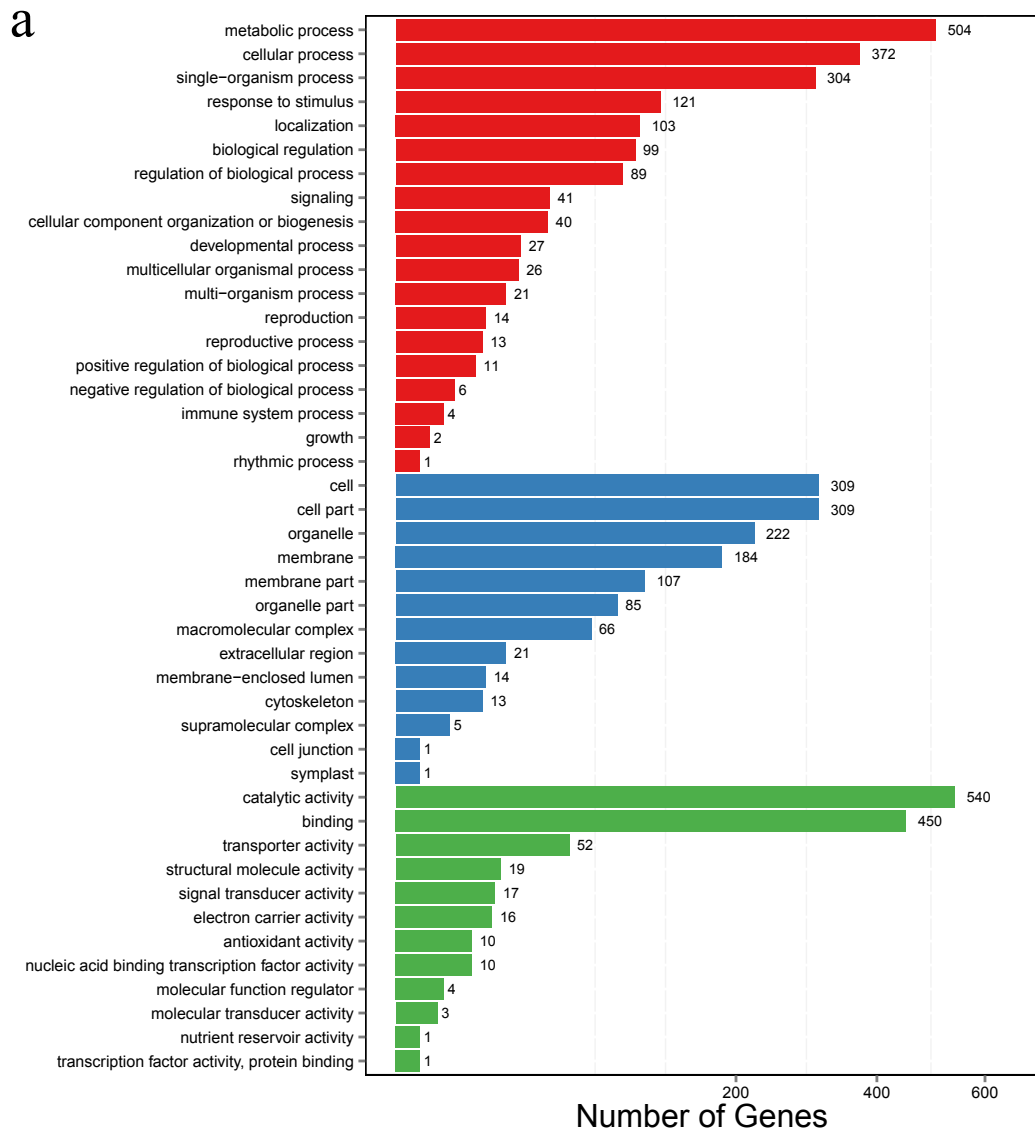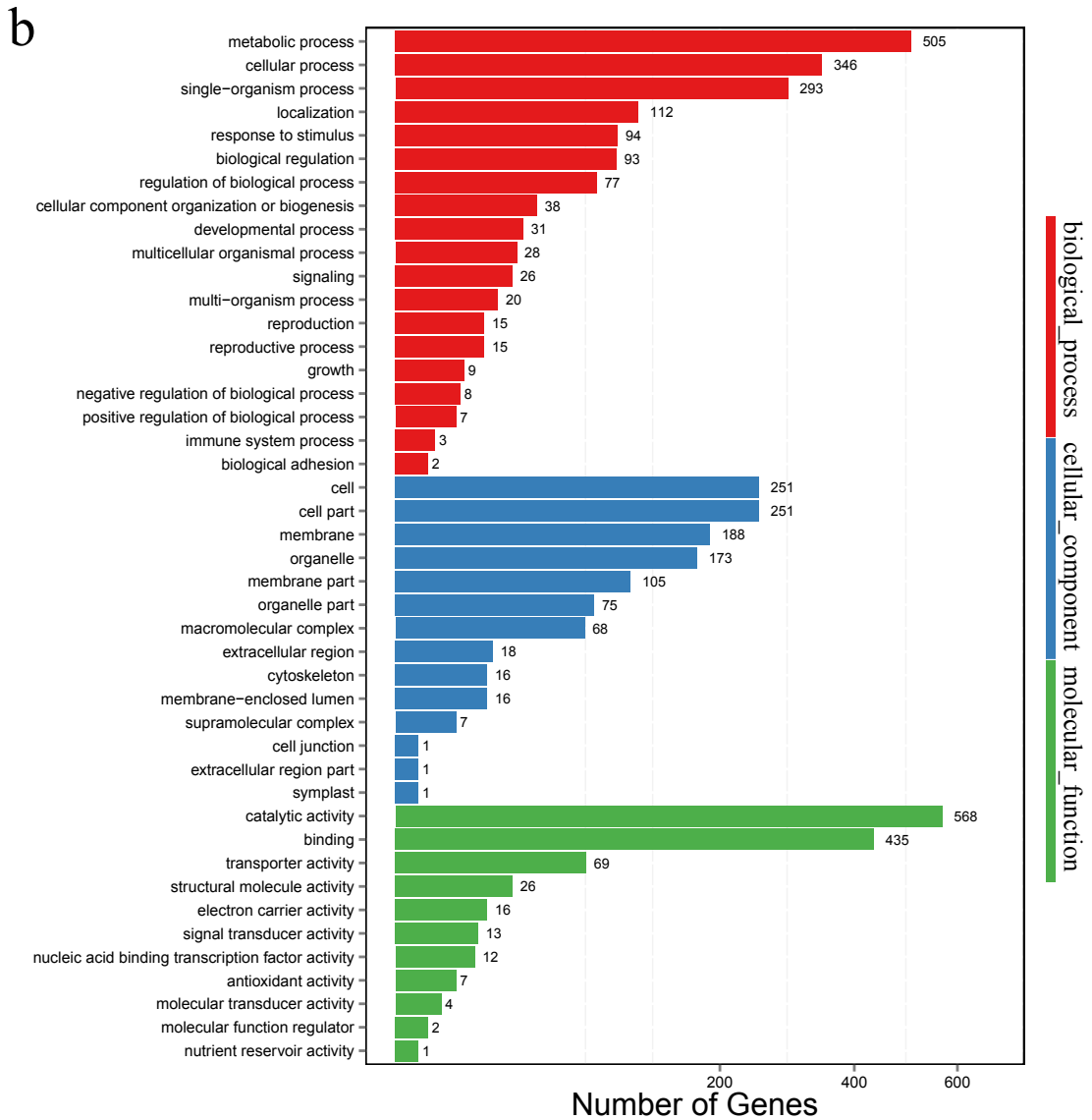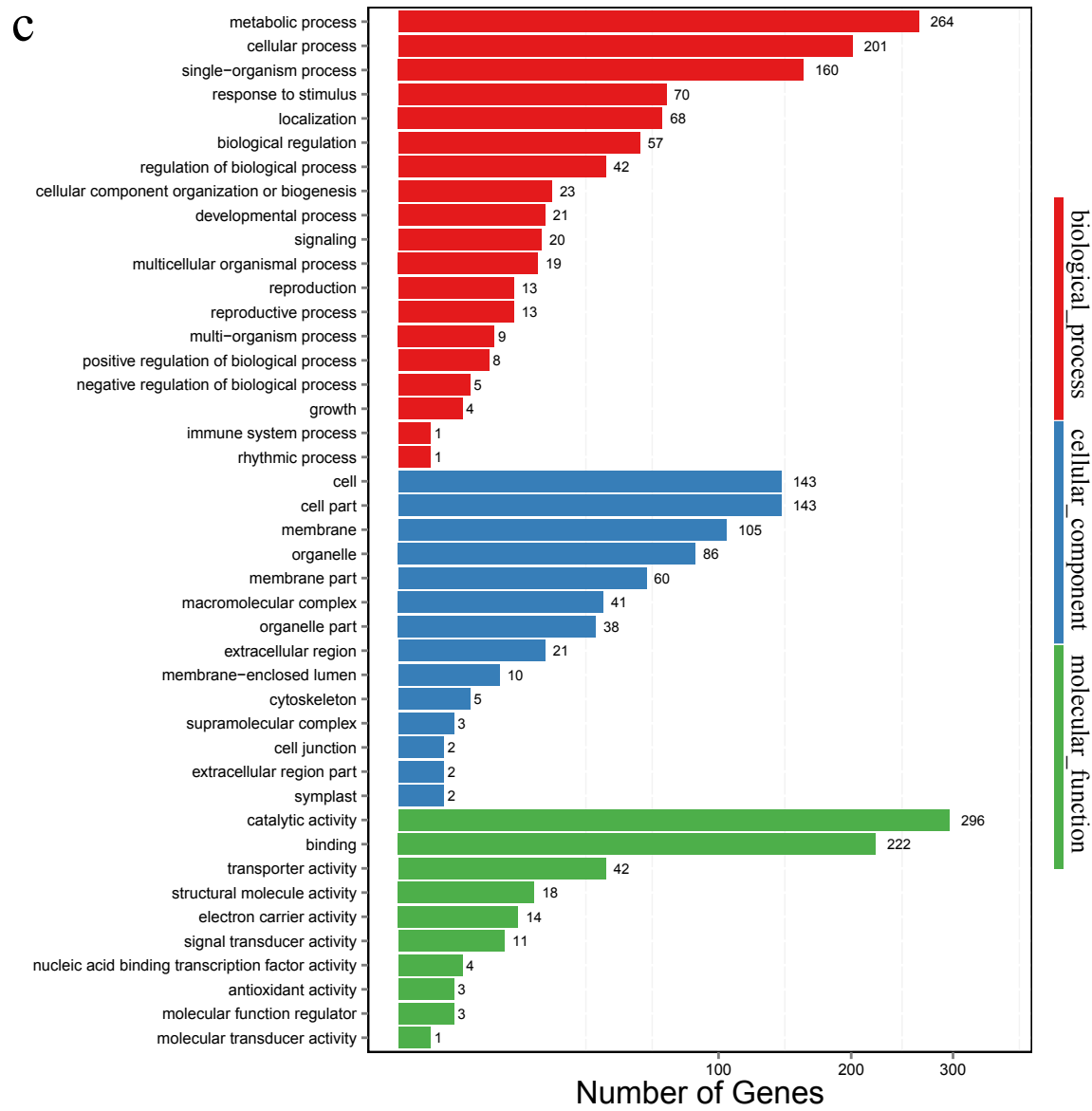

Supplement: Supplementary file 6 — Additional file 6: Figure S4. GO classification of differentially expressed genes. The number of DEGs is plotted along the x-axis; the GO terms is plotted along the y-axis. a T1 vs. T2 in the kernel. b T1 vs. T2 in the testa. c T1 vs. T2 in the pericarp. [file 12864_2021_7594_MOESM6_ESM.pdf]

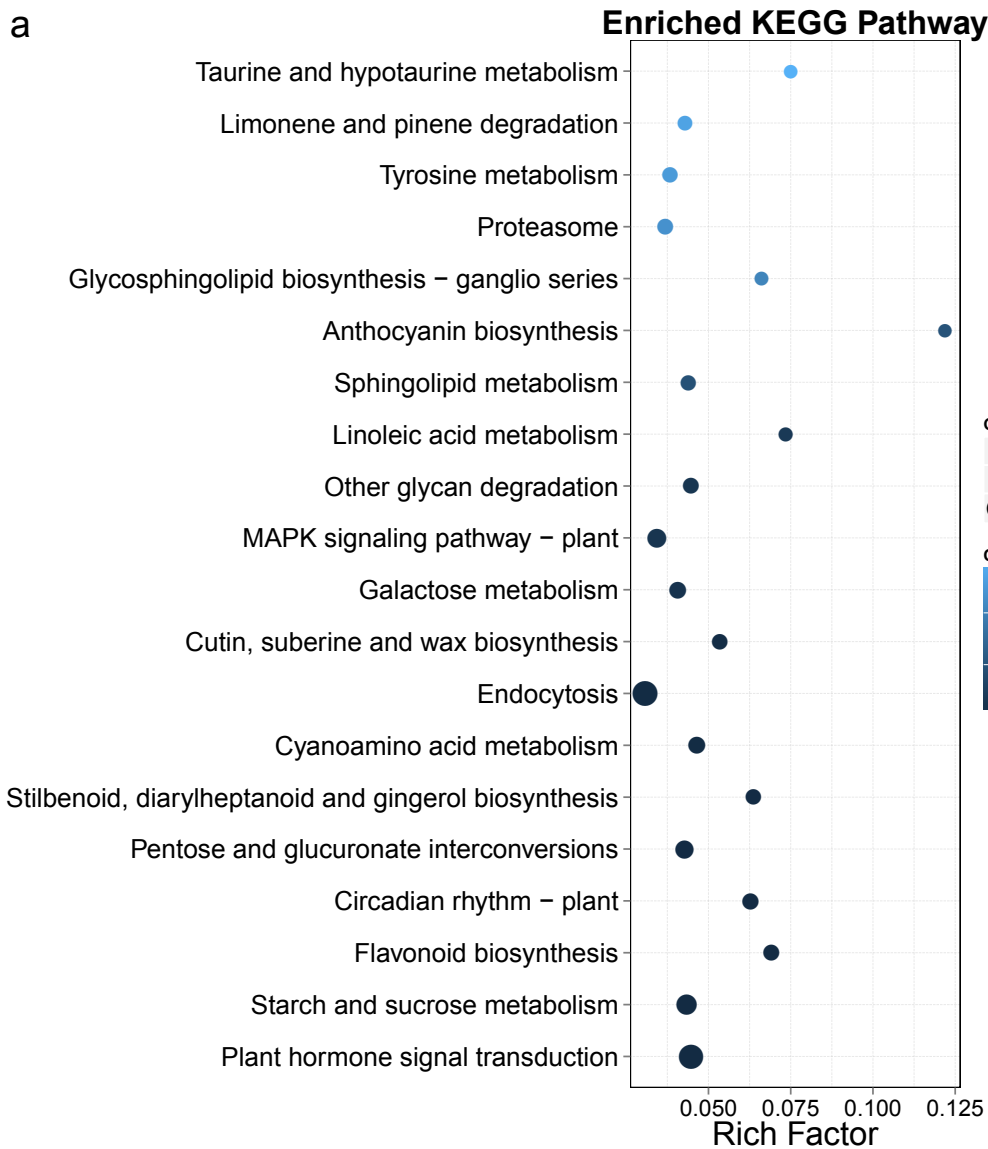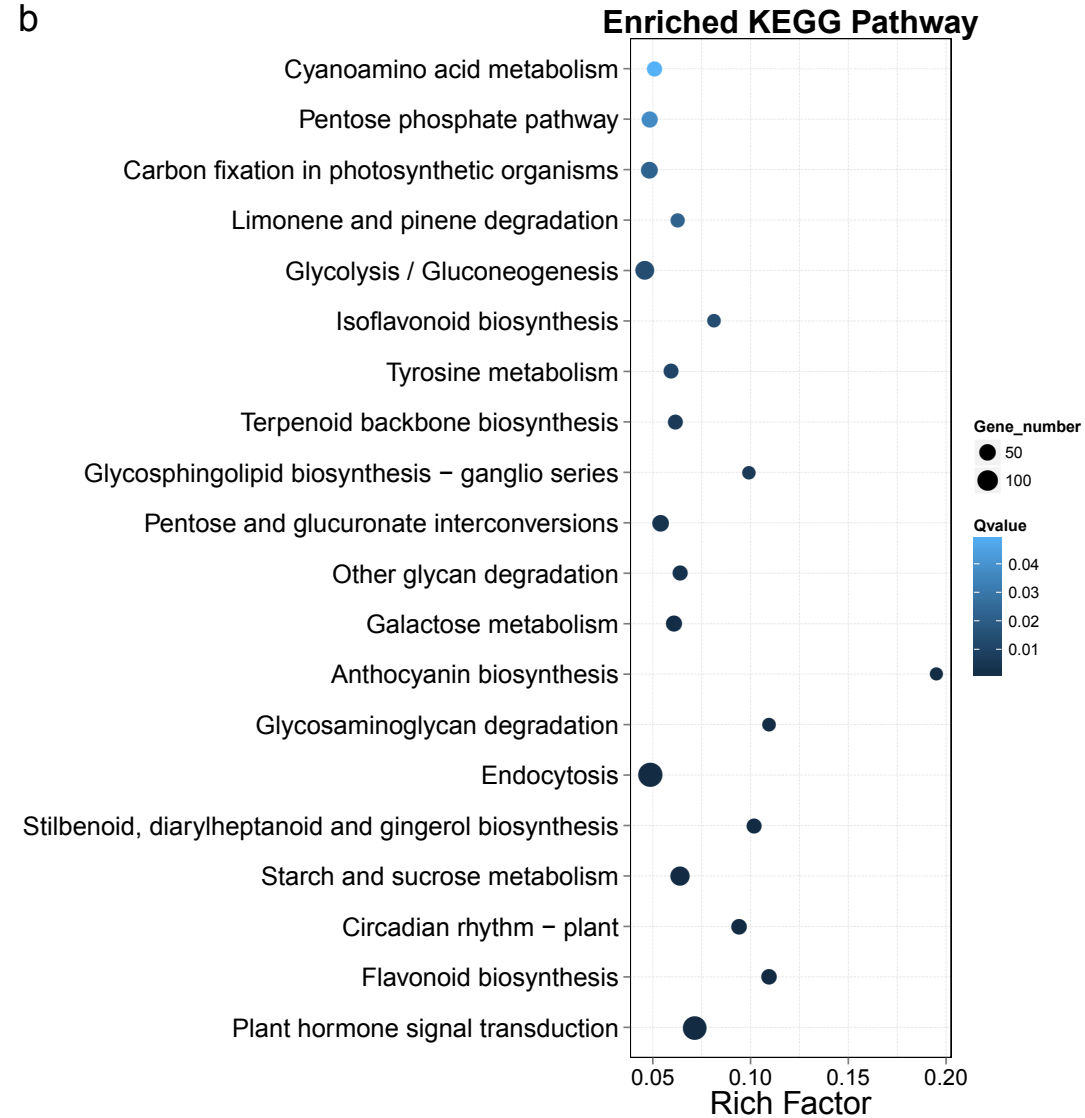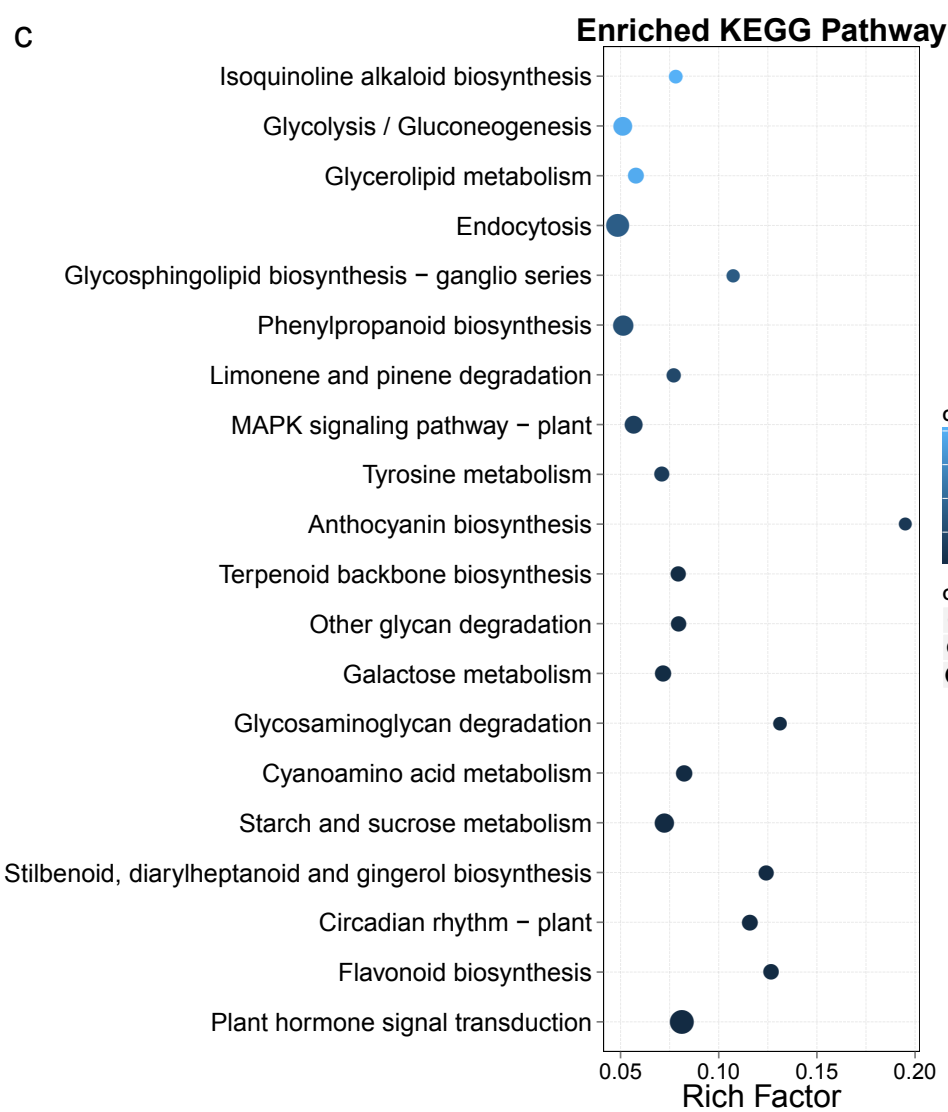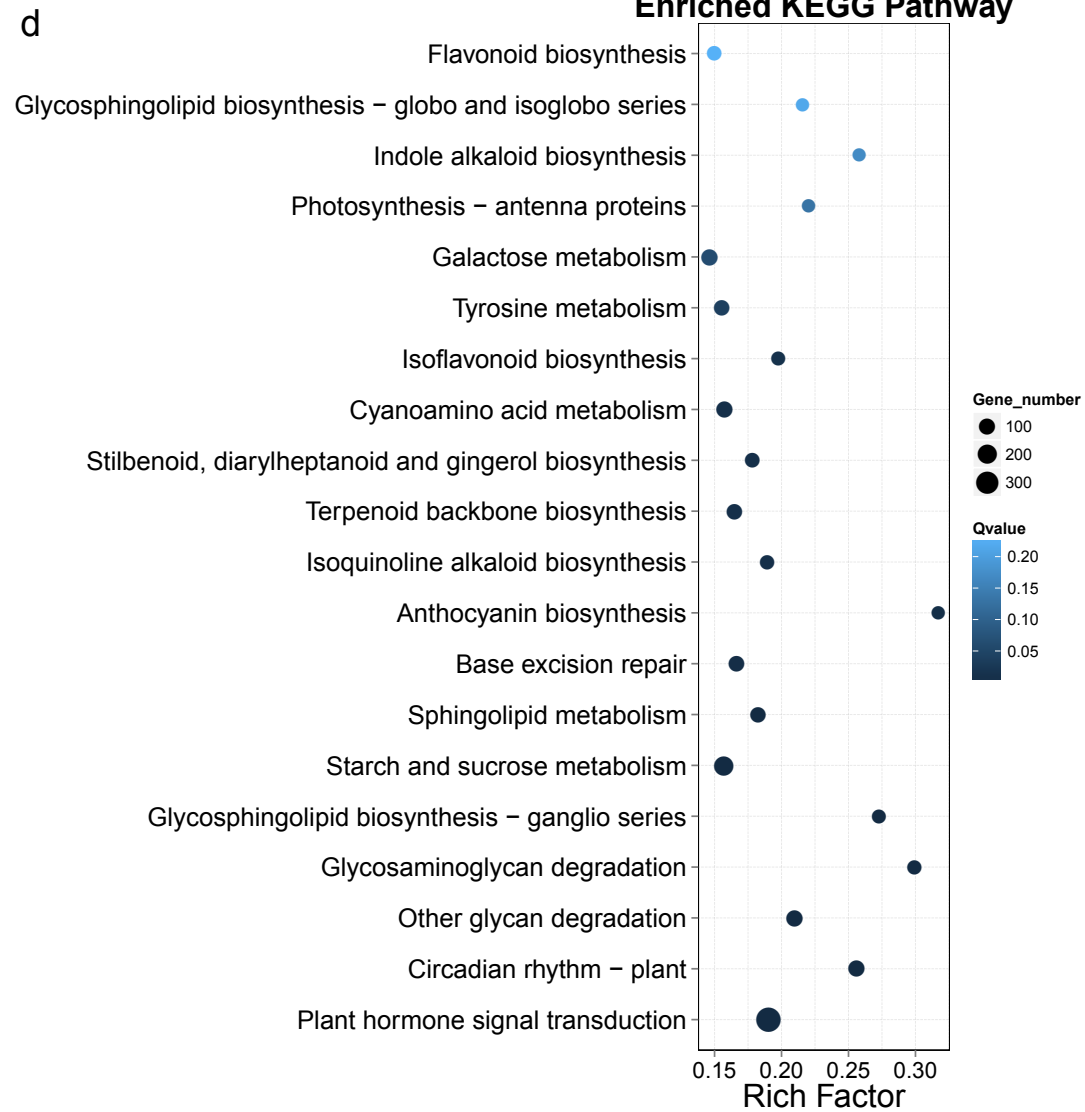

Supplement: Supplementary file 7 — Additional file 7: Figure S5. KEGG pathways enriched in the genes differentially expressed in the developing kernel at various time points. The enrichment factor is plotted on the x-axis; pathway names are shown on the y-axis. The color of each dot reflects the Qvalue, while the size of the dot represents the number of DEGs. a T1 vs. T3. b T1 vs. T4. c T1 vs. T5. d T1 vs. T6. [file 12864_2021_7594_MOESM7_ESM.pdf]

a

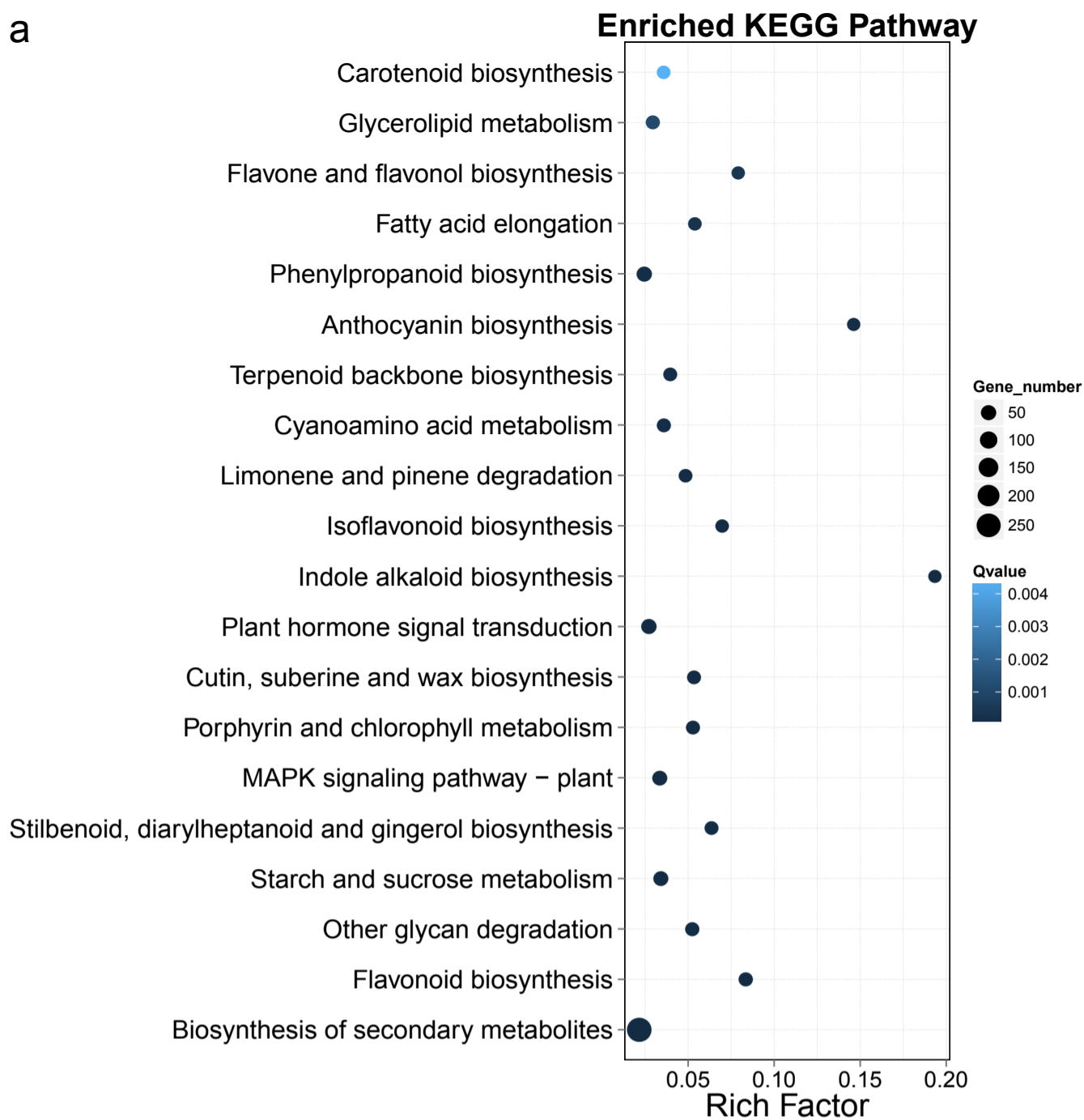

b

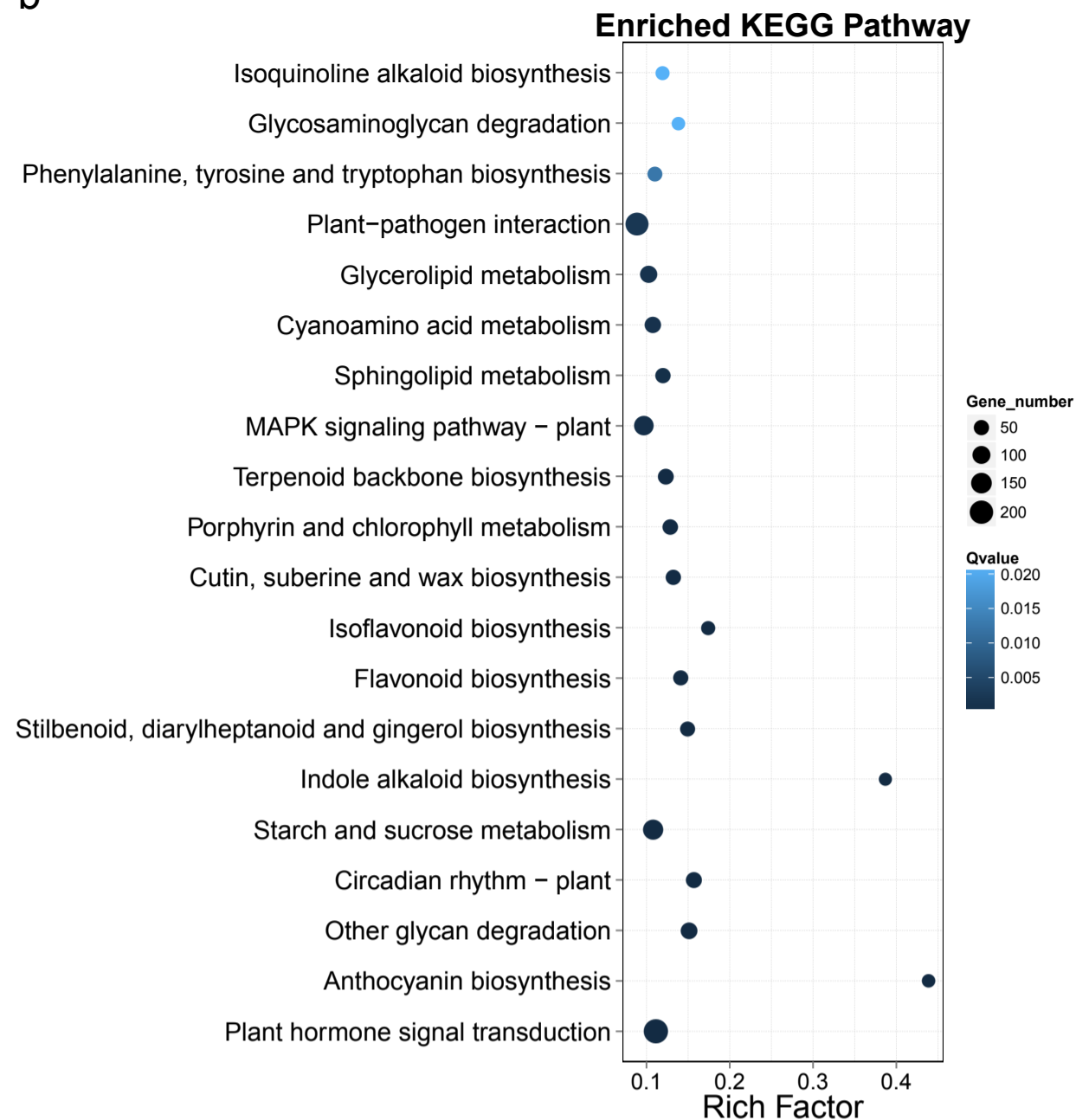

c

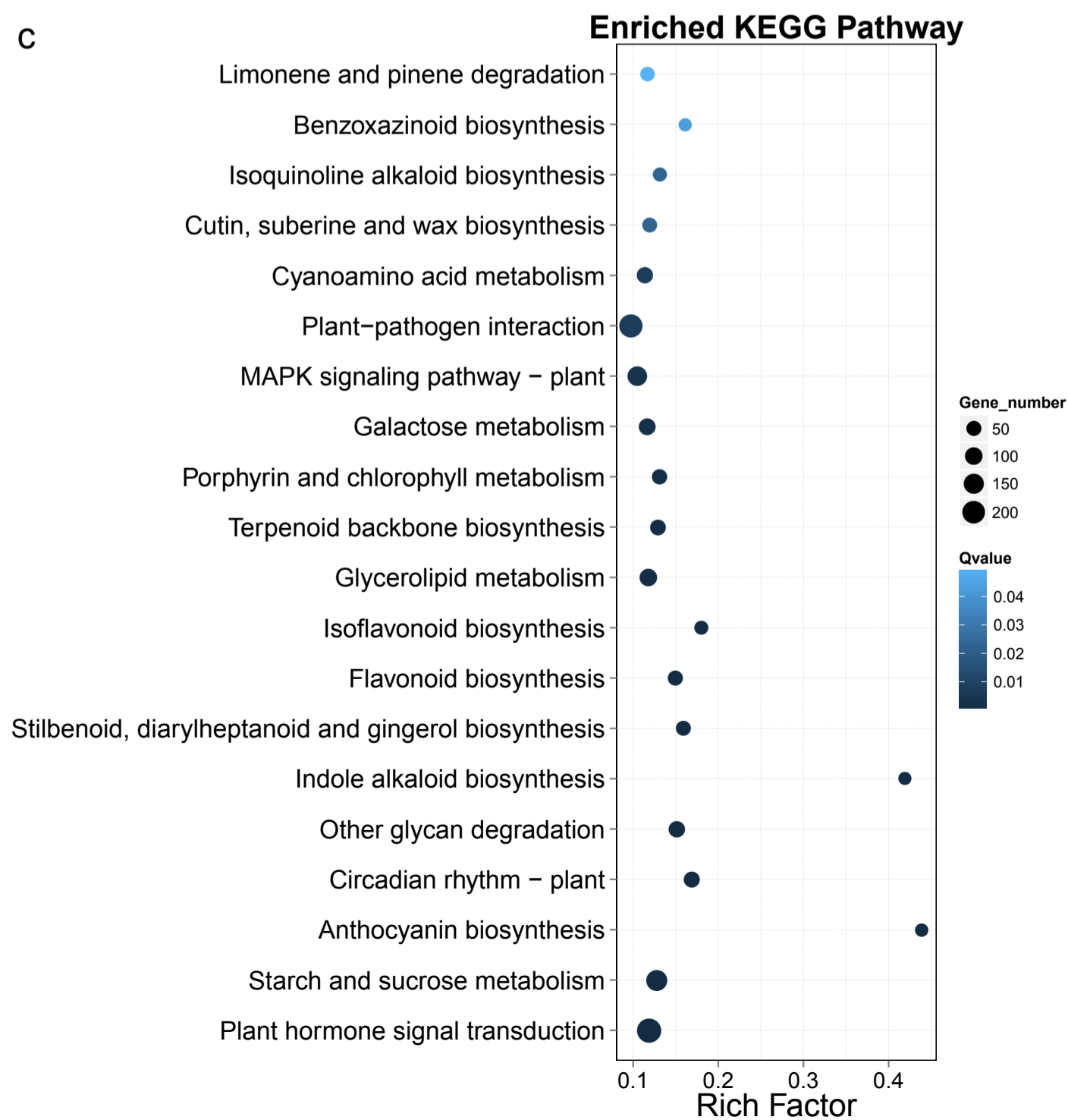

Supplement: Supplementary file 8 — Additional file 8: Figure S6. KEGG pathways enriched in the genes differentially expressed in the developing testa at various time points. The enrichment factor is plotted on the x-axis; pathway names are shown on the y-axis. The color of each dot reflects the Qvalue, while the size of the dot represents the number of DEGs. a T1 vs. T4. b T1 vs. T5. c T1 vs. T6. [file 12864_2021_7594_MOESM8_ESM.pdf]

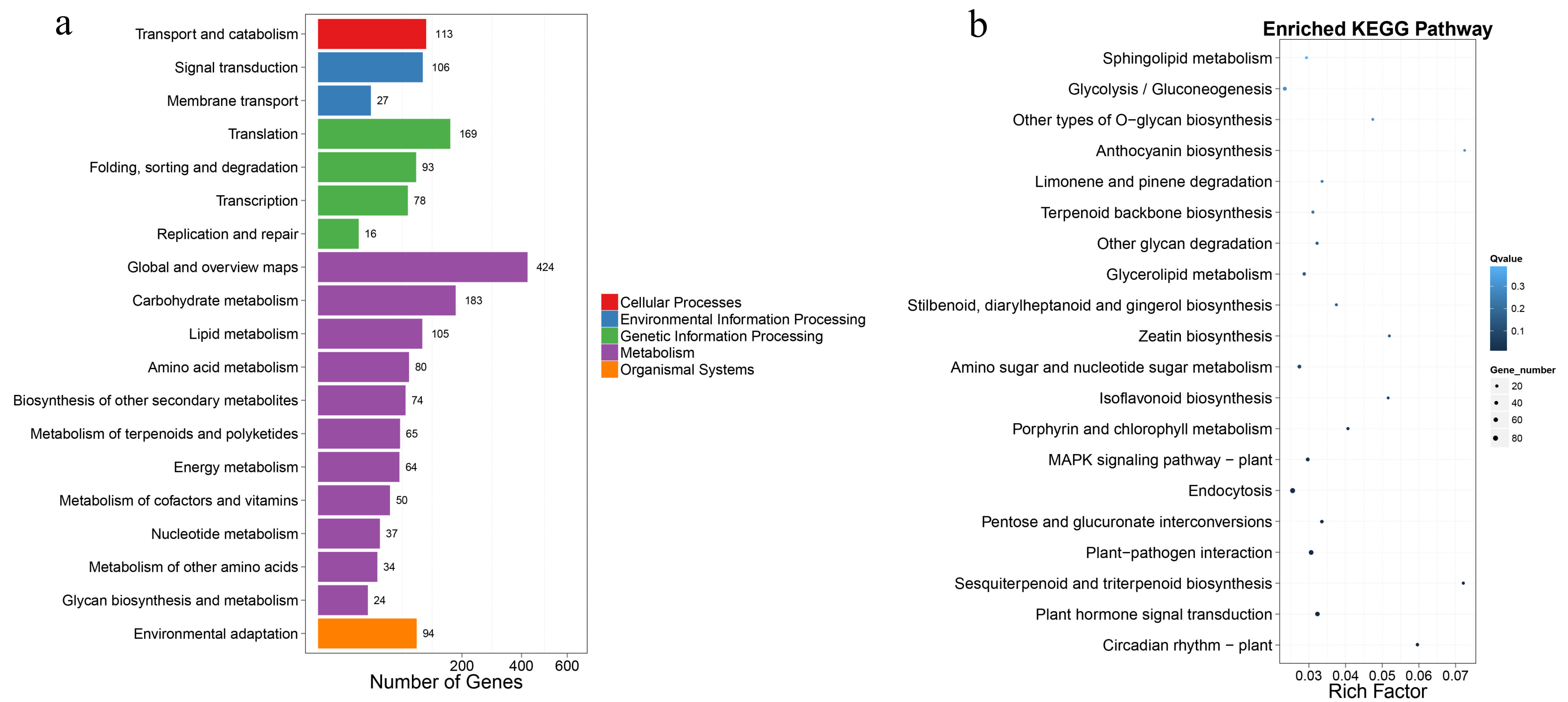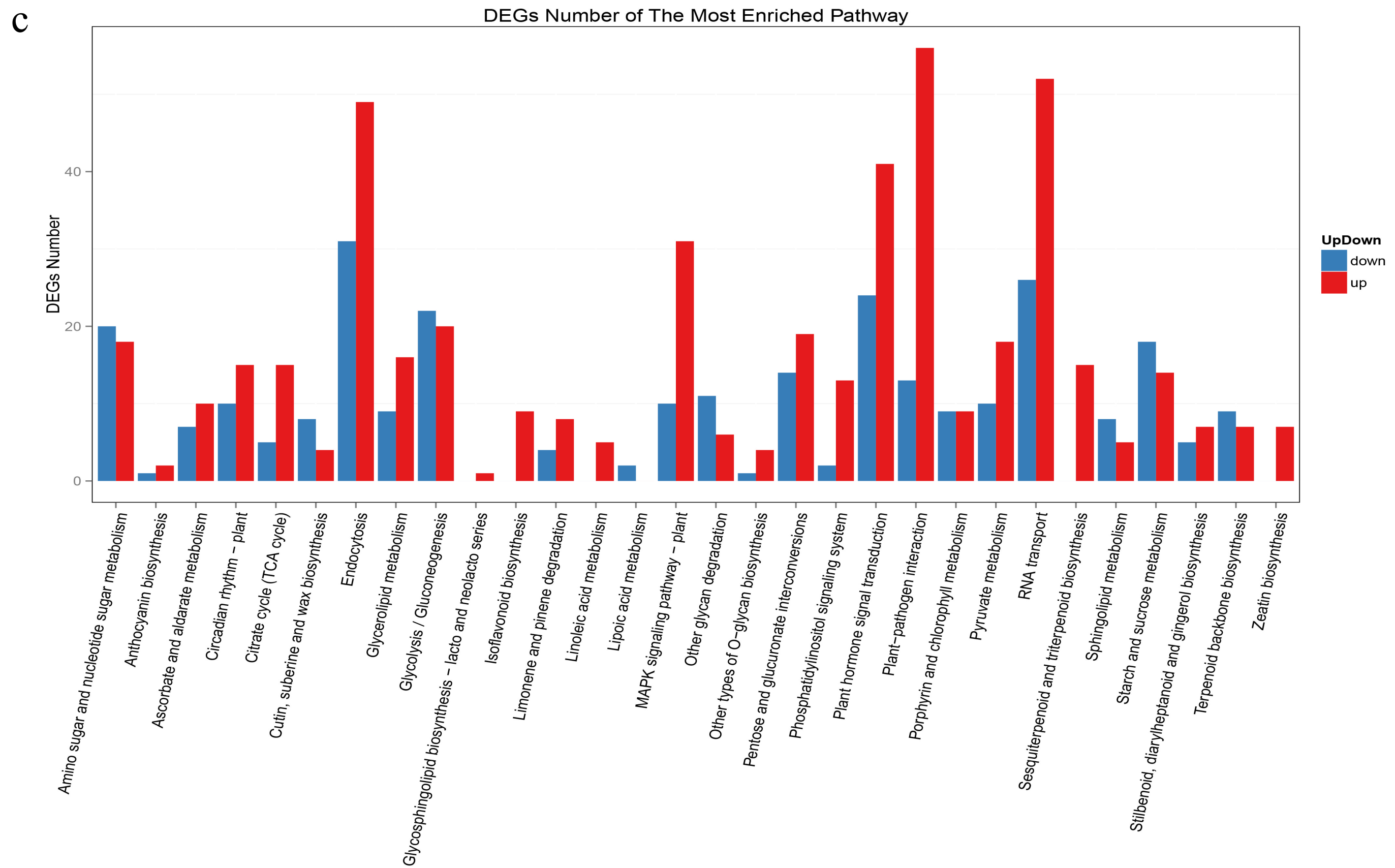

Supplement: Supplementary file 9 — Additional file 9: Figure S7. KEGG pathways enriched in the genes differentially expressed in the developing pericarp at various time points. a The number of genes associated with various KEGG pathways differentially expressed between T1 and T2. b The KEGG pathways overrepresented in the genes differentially expressed between T1 and T2. The color of each dot reflects the Qvalue, while the size of the dot represents the number of DEGs. c The up- and downregulated genes in T2 as compared to T1 that were associated with each KEGG pathway. [file 12864_2021_7594_MOESM9_ESM.pdf]

a

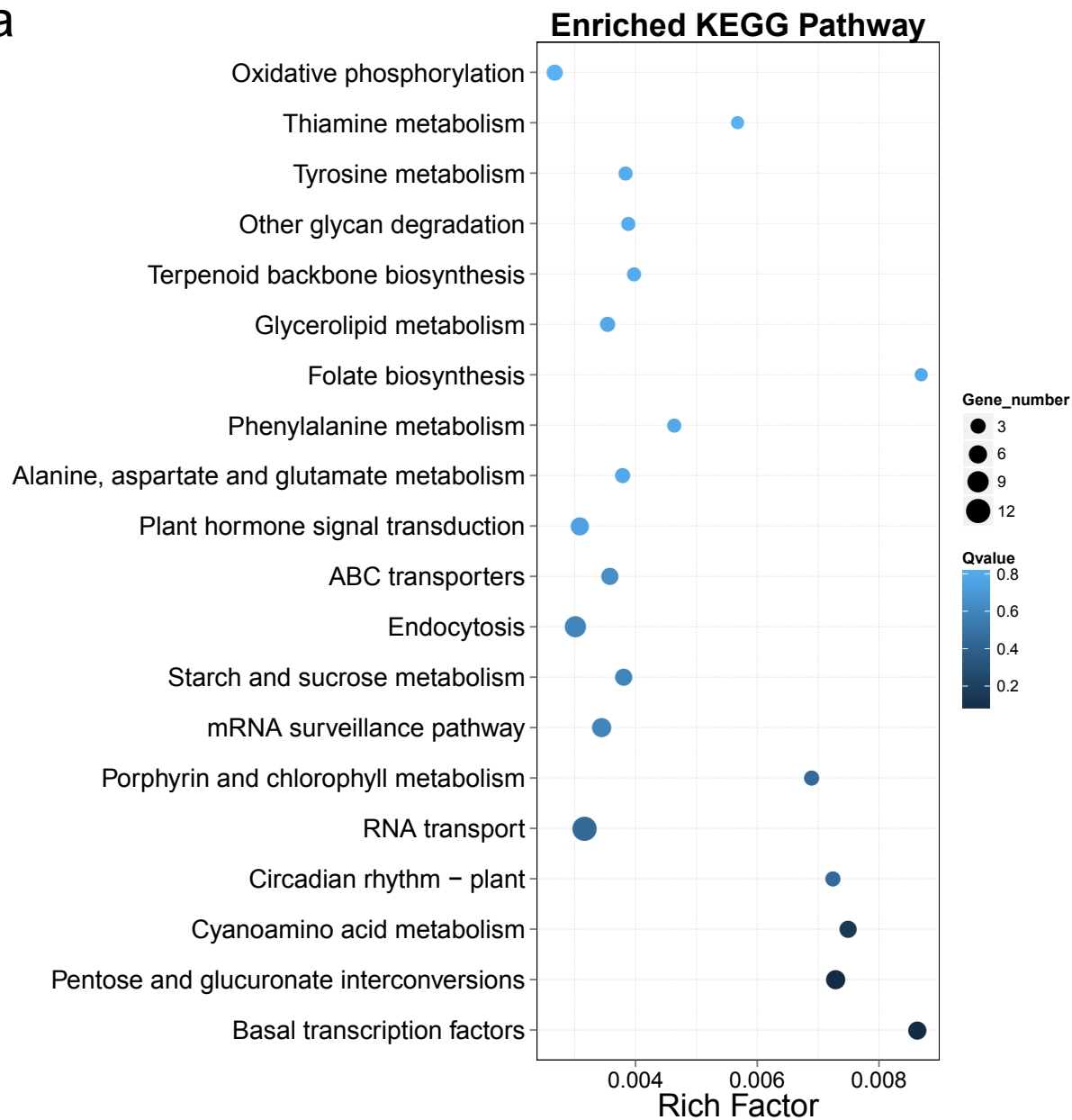

b

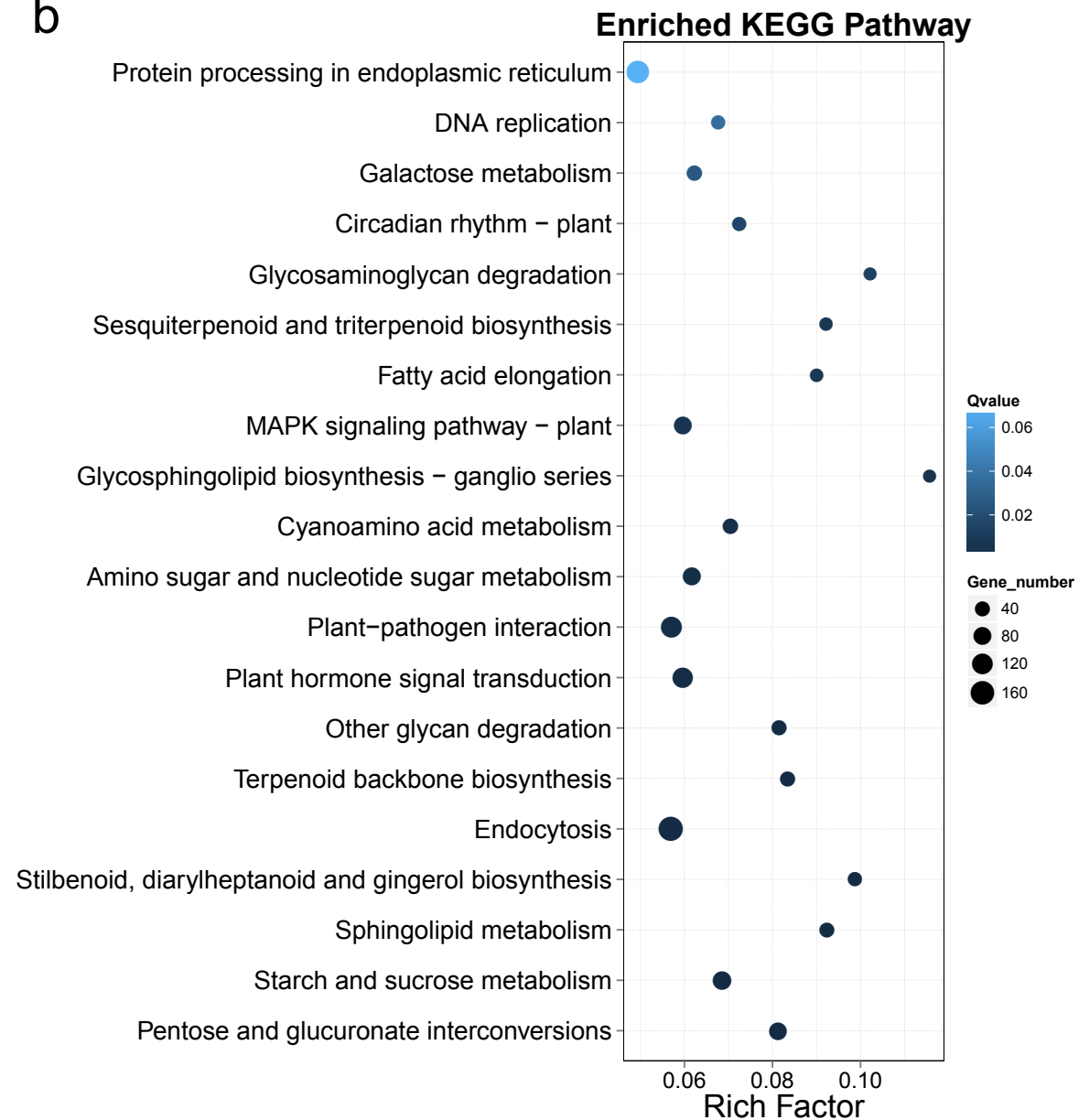

c

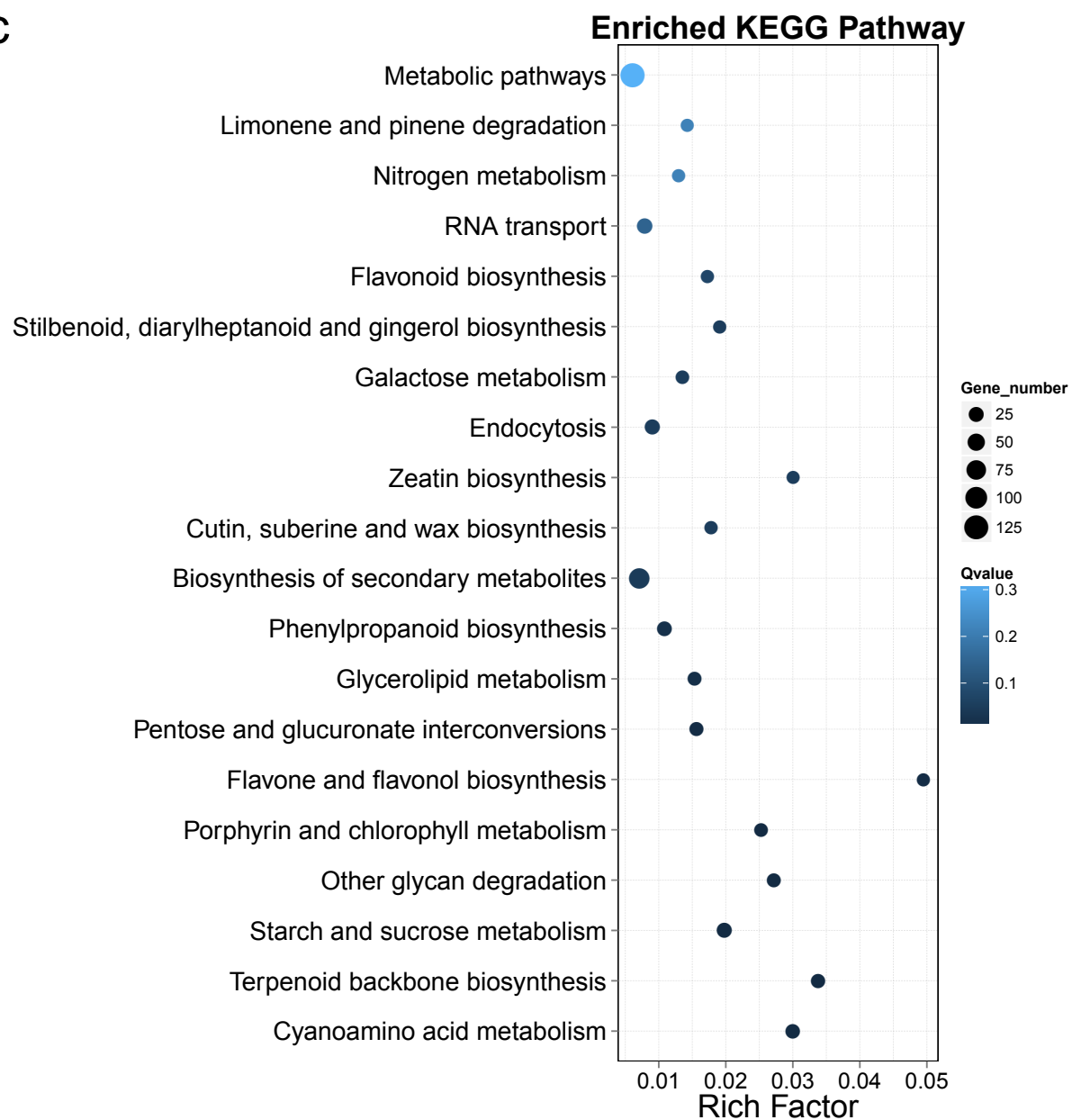

d

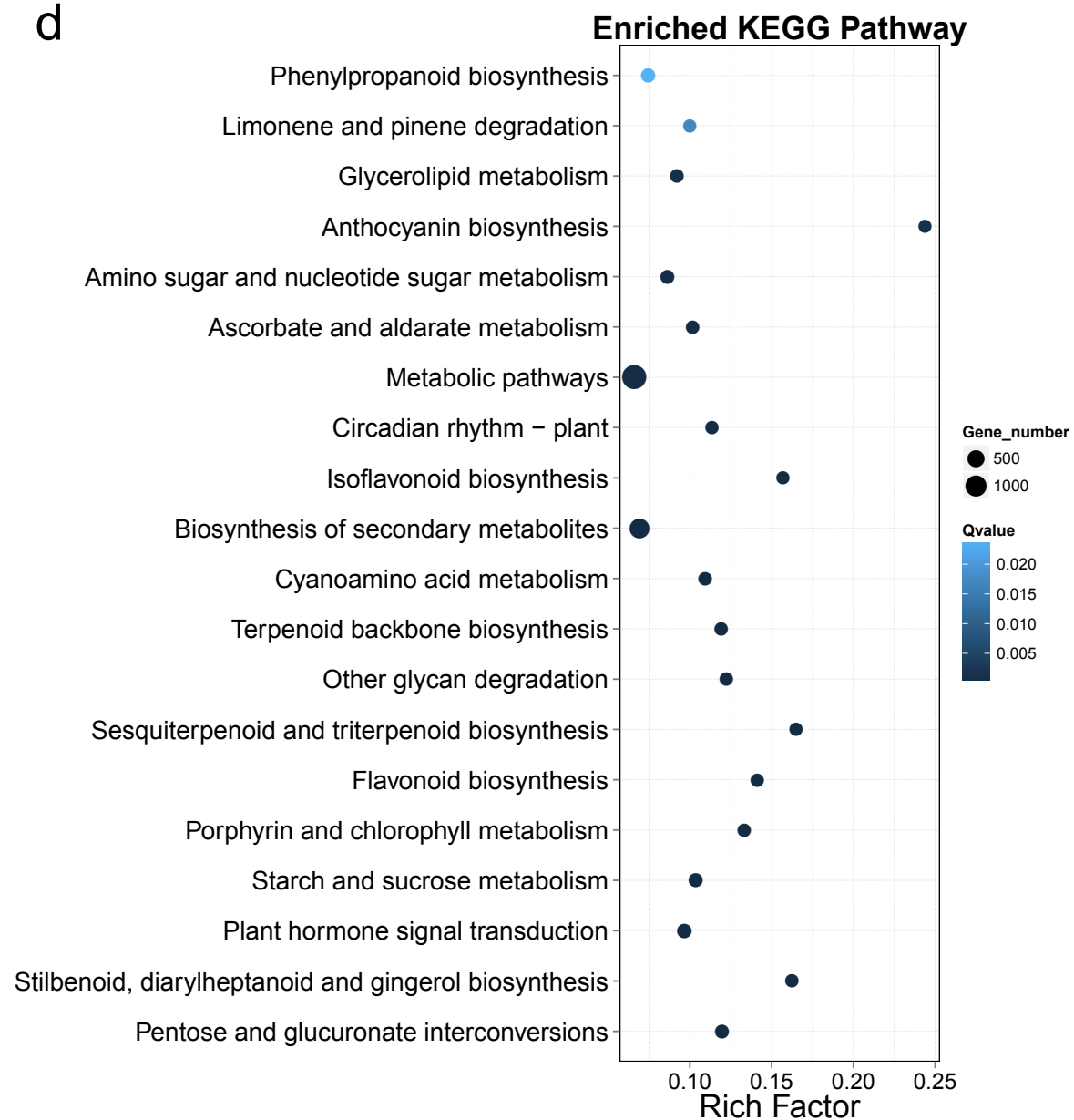

Supplement: Supplementary file 10 — Additional file 10: Figure S8. KEGG pathways enriched in the genes differentially expressed in the developing pericarp at various time points. The enrichment factor is plotted on the x-axis; pathway names are shown on the y-axis. The color of each dot reflects the Qvalue, while the size of the dot represents the number of DEGs. a T1 vs. T3. b T1 vs. T4. c T1 vs. T5. d T1 vs. T6. [file 12864_2021_7594_MOESM10_ESM.pdf]

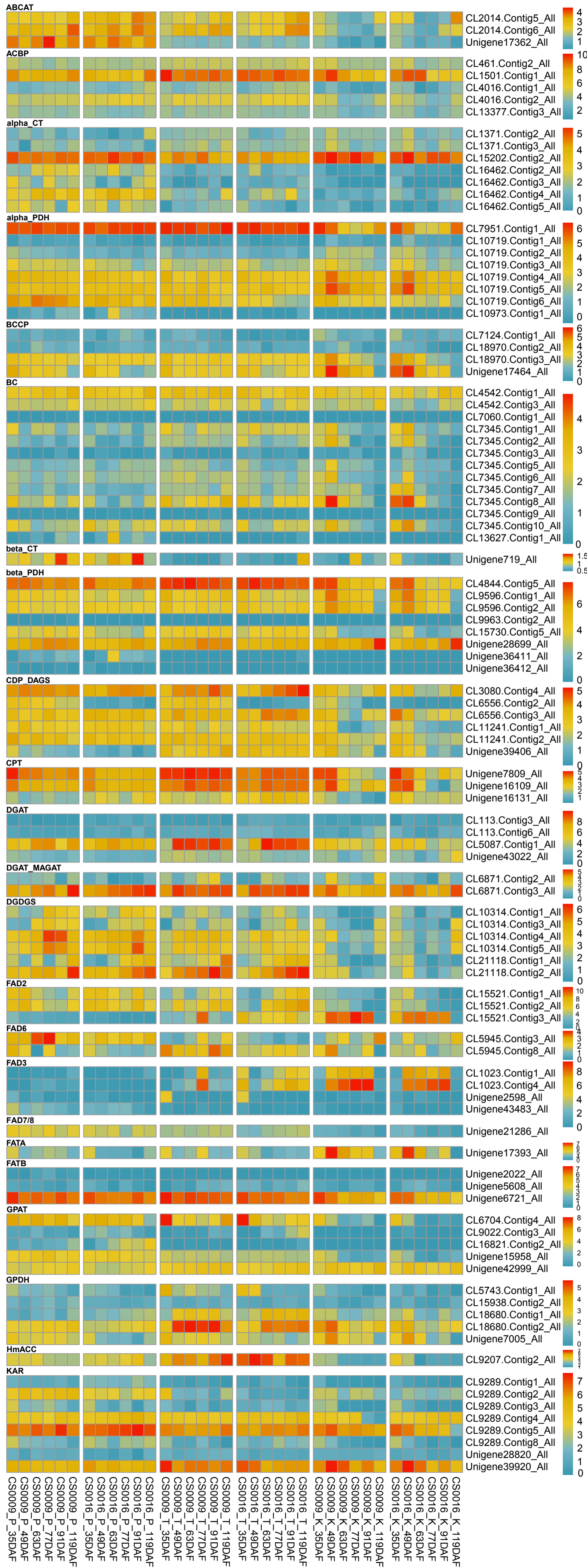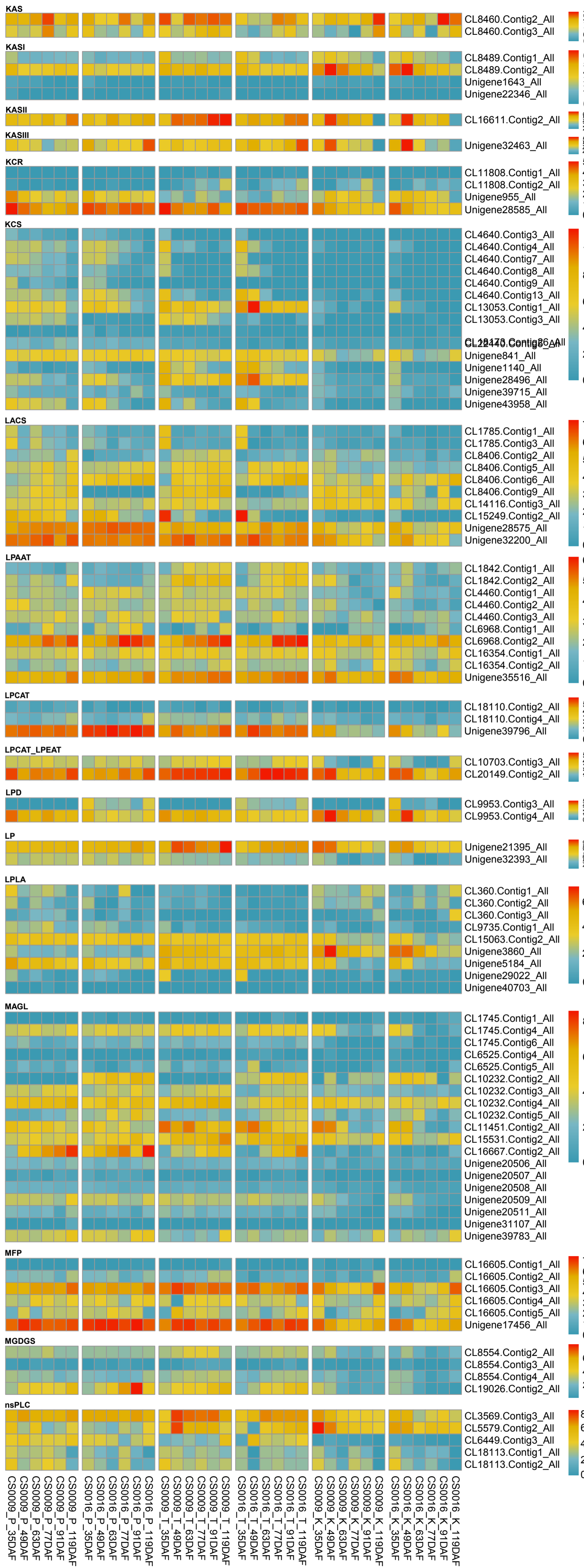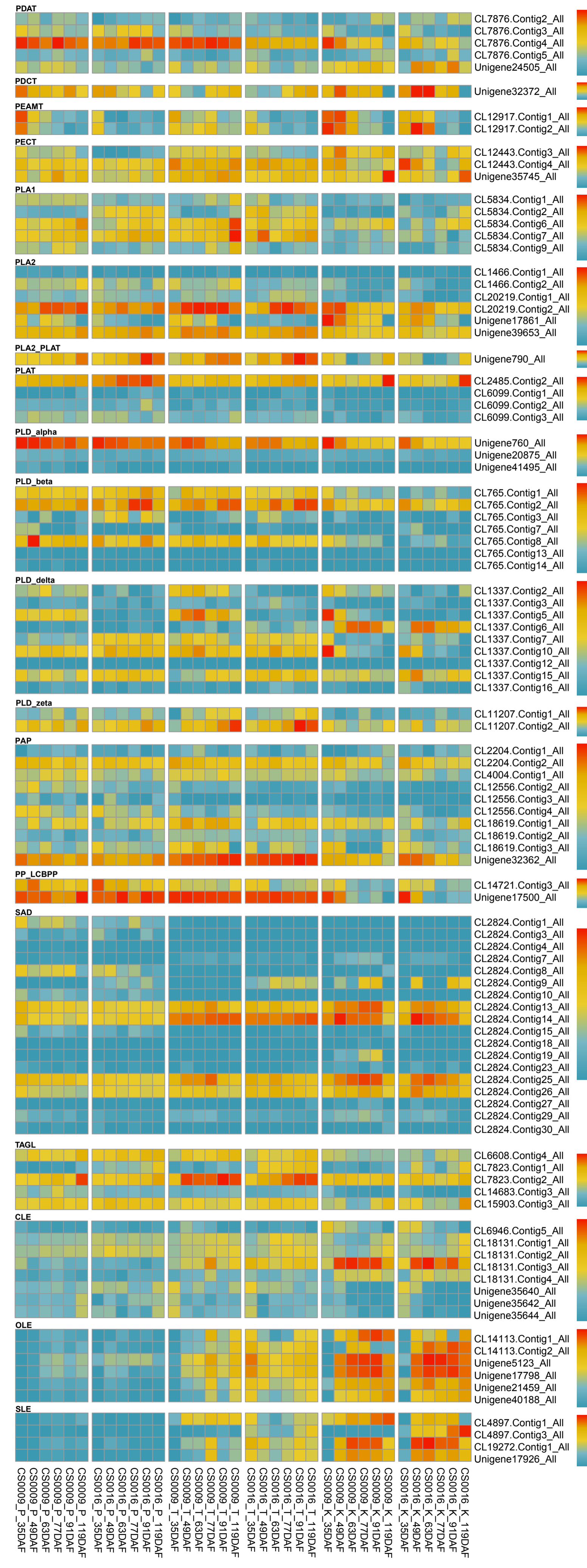

Supplement: Supplementary file 13 — Additional file 13: Figure S9. Heat map showing the expression patterns of the unigenes associated with lipid metabolism during the development of the seed kernel (k), seed testa (t), and fruit pericarp (p) of Paeonia ostii. Labels on the y-axis indicate which plant was used (CS0009 or CS0016), the tissue (k, t, or p), and the developmental period (35–119 days after fertilization, DAF). [file 12864_2021_7594_MOESM13_ESM.pdf]

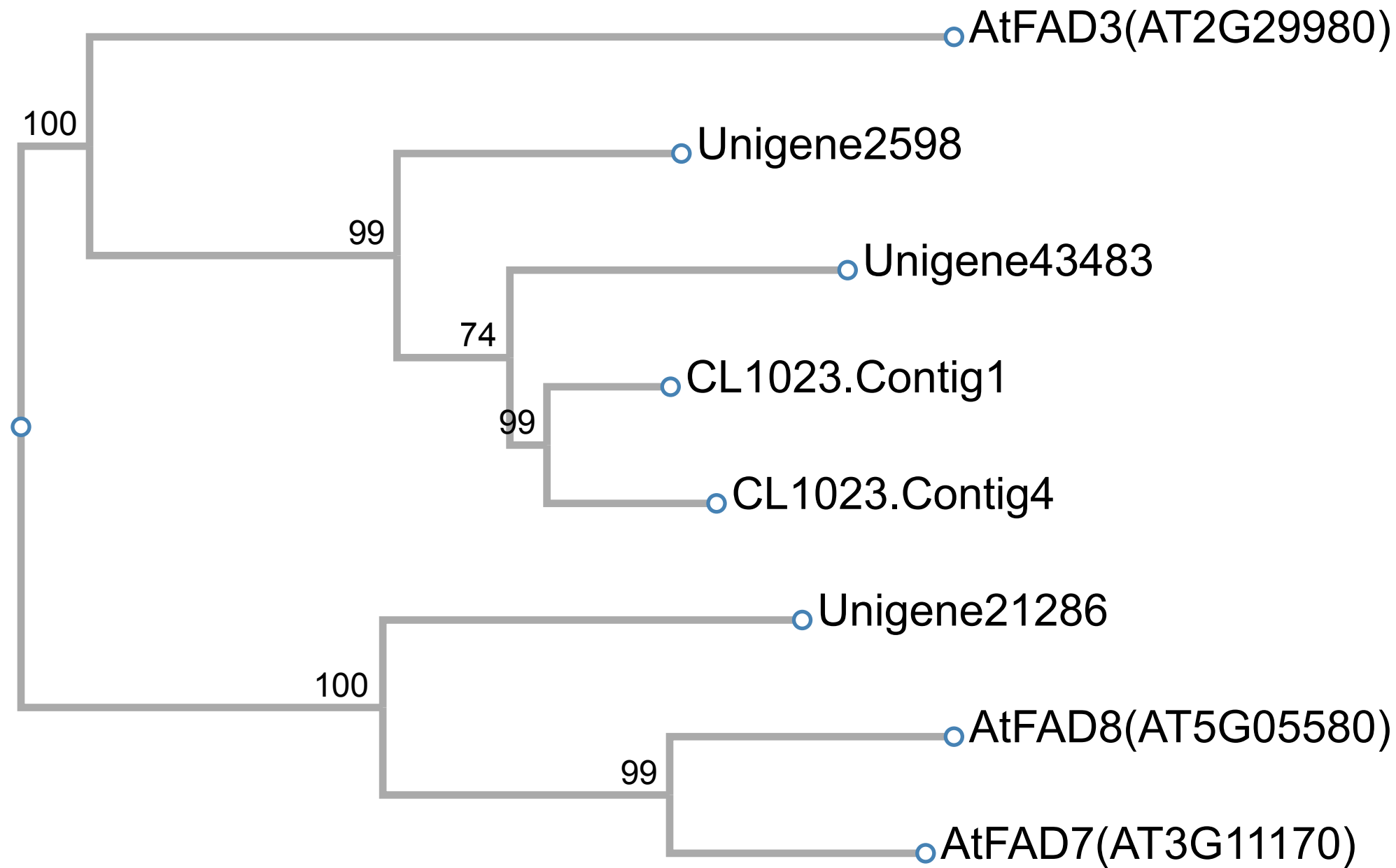

Supplement: Supplementary file 14 — Additional file 14: Figure S10. Phylogenetic relationships among ω-3 FADs from Paeonia ostii and Arabidopsis thaliana. [file 12864_2021_7594_MOESM14_ESM.pdf]

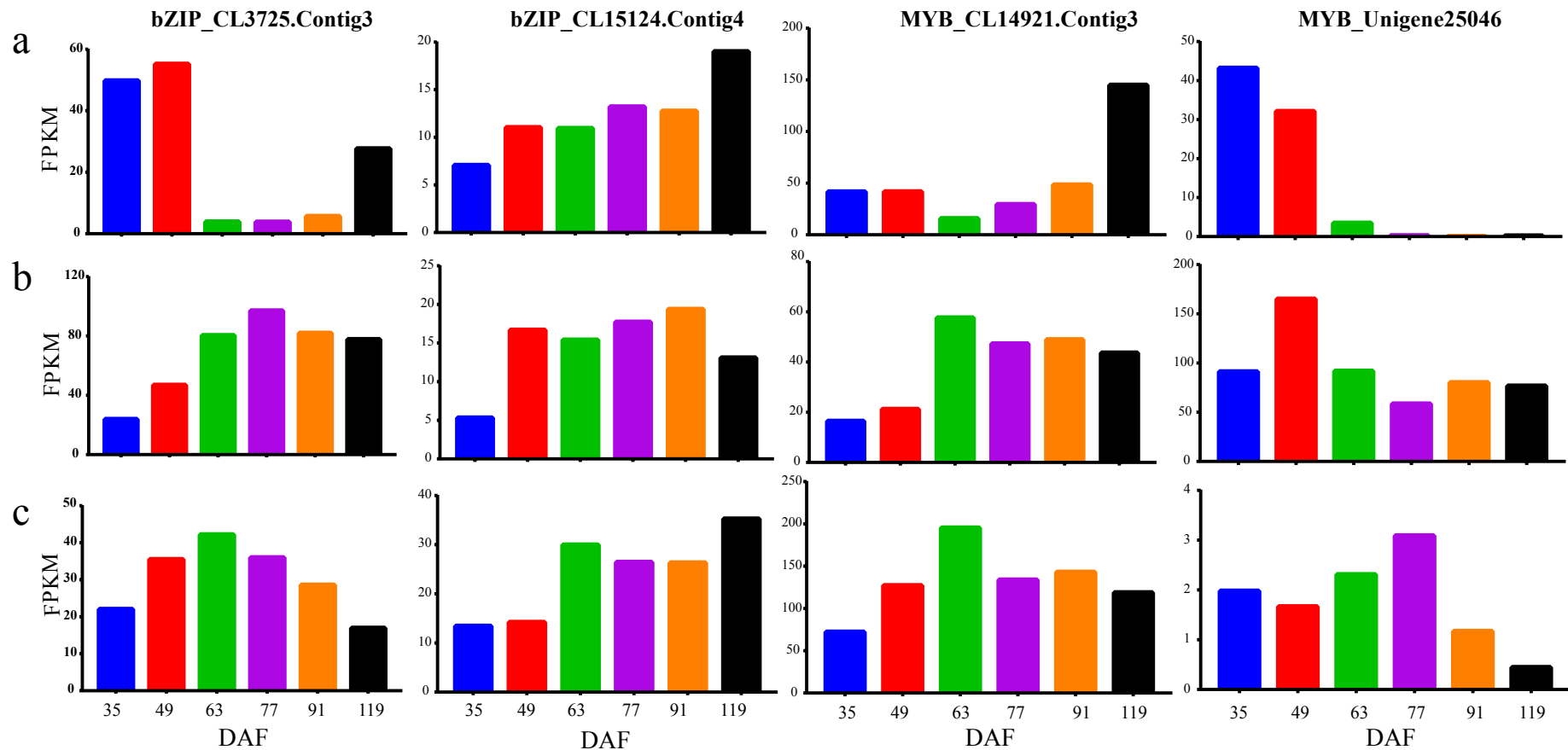

Supplement: Supplementary file 16 — Additional file 16: Figure S11. The relative expression levels of genes associated with key TFs. a Kernel. b Testa. c Pericarp. [file 12864_2021_7594_MOESM16_ESM.pdf]

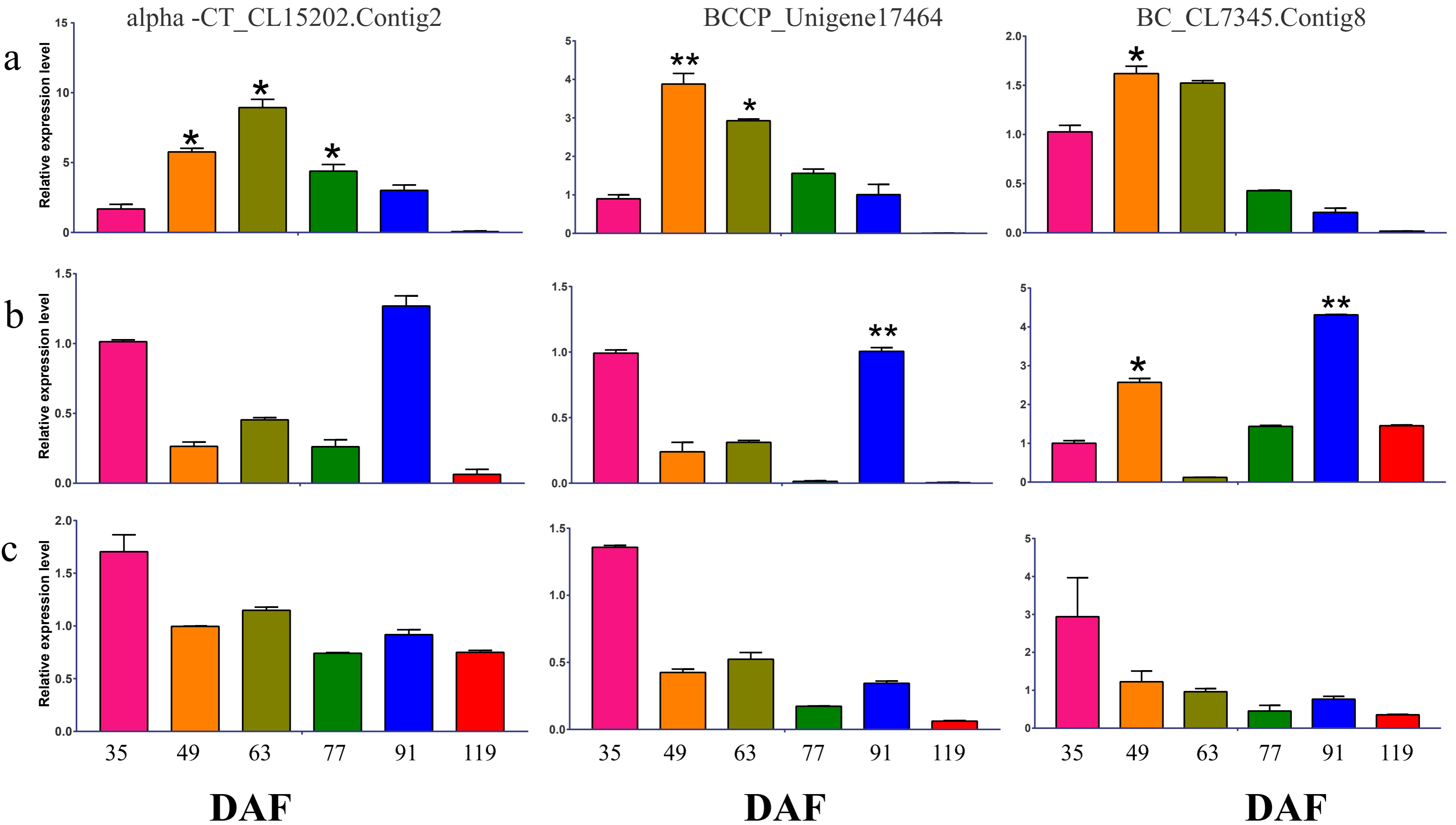

Supplement: Supplementary file 17 — Additional file 17: Figure S12. Quantitative real-time PCR validation of ACCase genes. a Kernel. b Testa. c Pericarp. [file 12864_2021_7594_MOESM17_ESM.pdf]

FAD6\_CL5945.Contig8

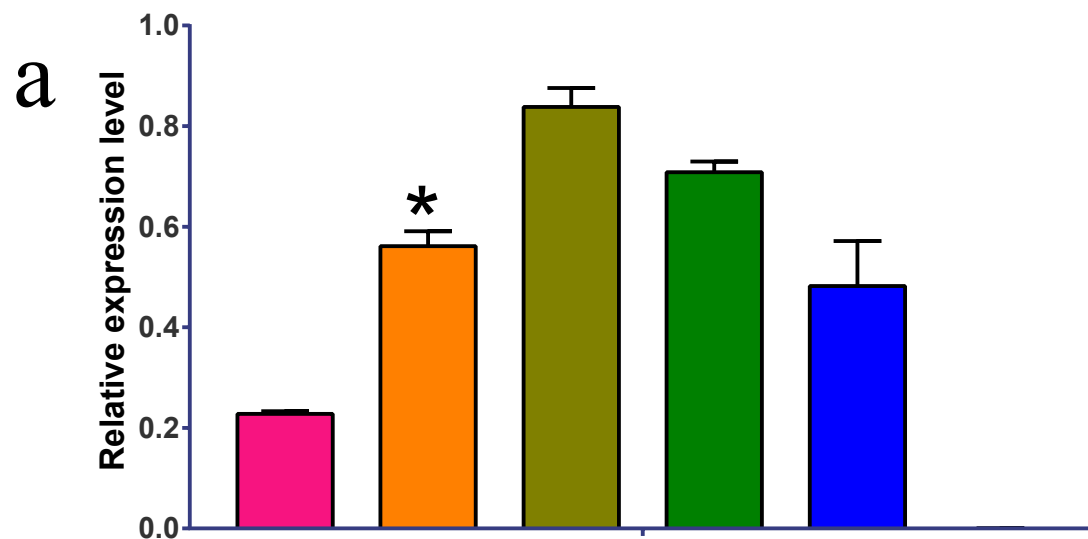

FAD7/8\_Unigene21286

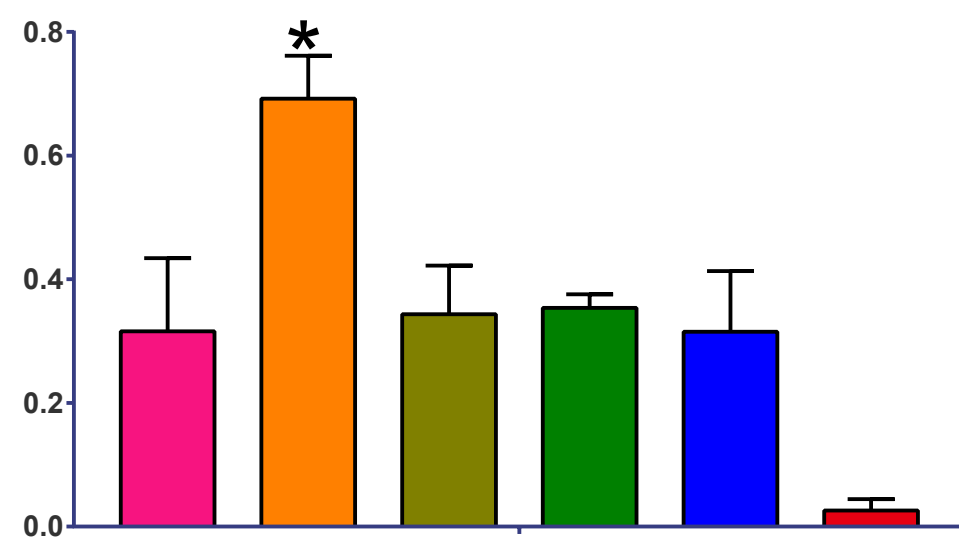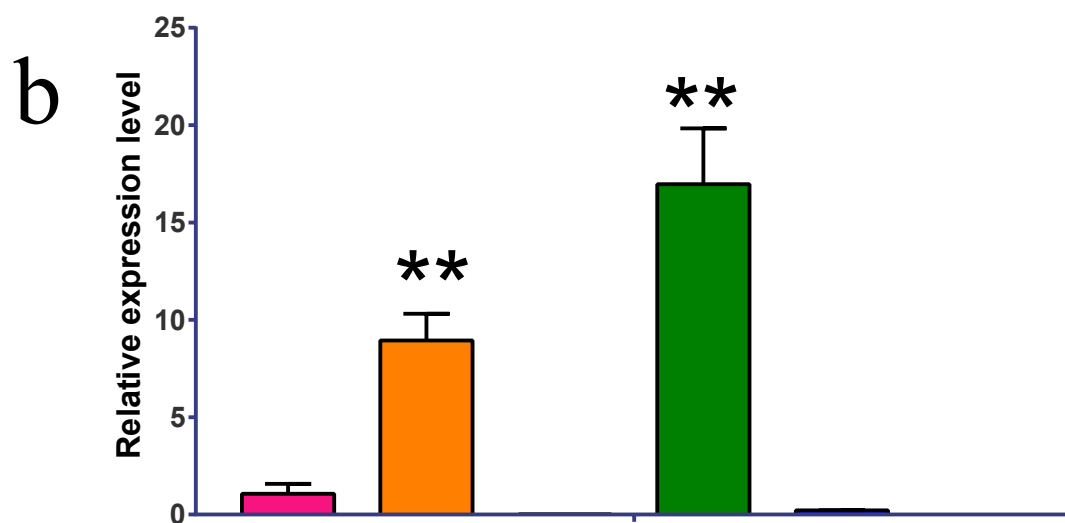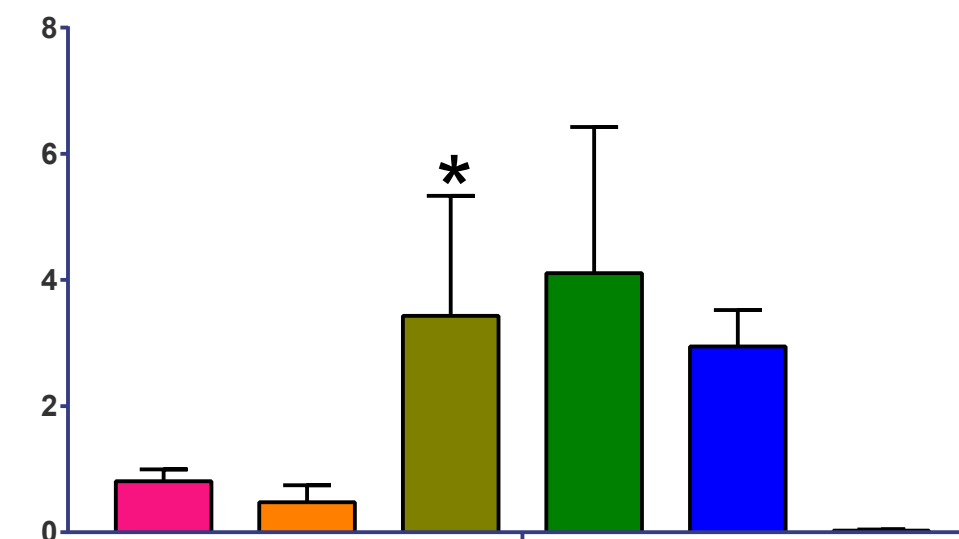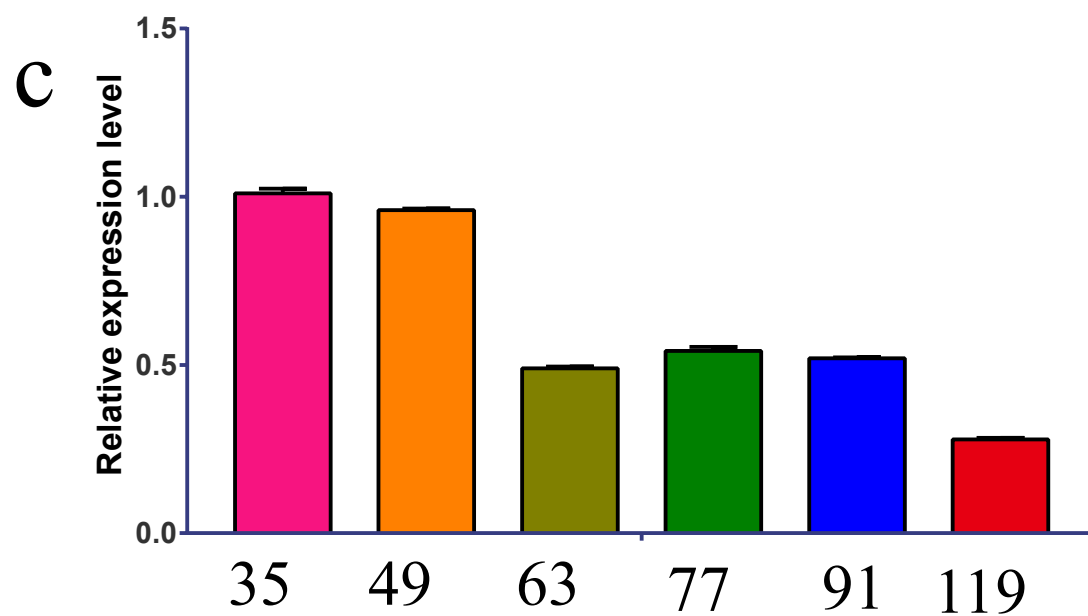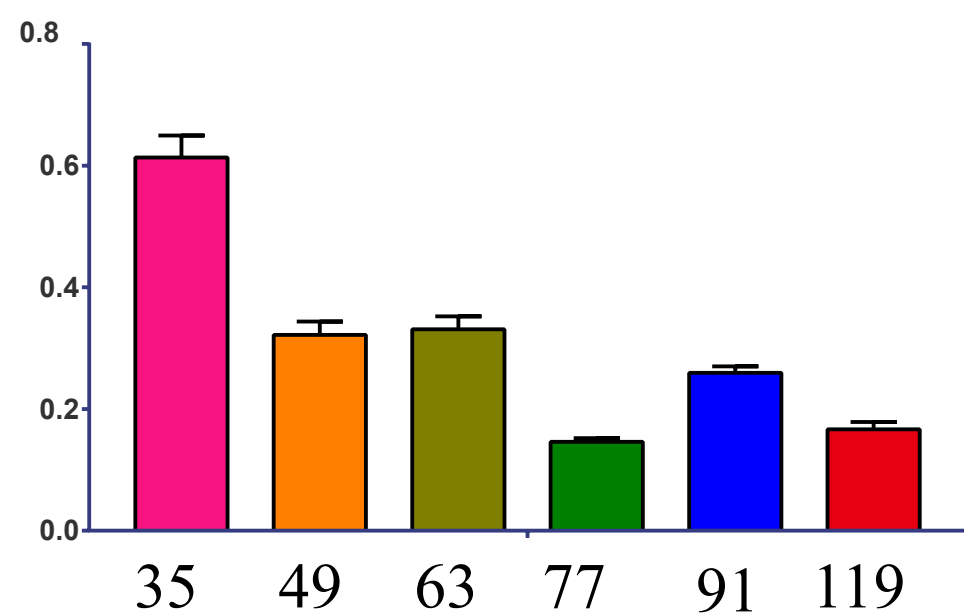**DAF****DAF**

Supplement: Supplementary file 18 — Additional file 18: Figure S13. Quantitative real-time PCR validation of FAD genes. a Kernel. b Testa. c Pericarp. [file 12864_2021_7594_MOESM18_ESM.pdf]

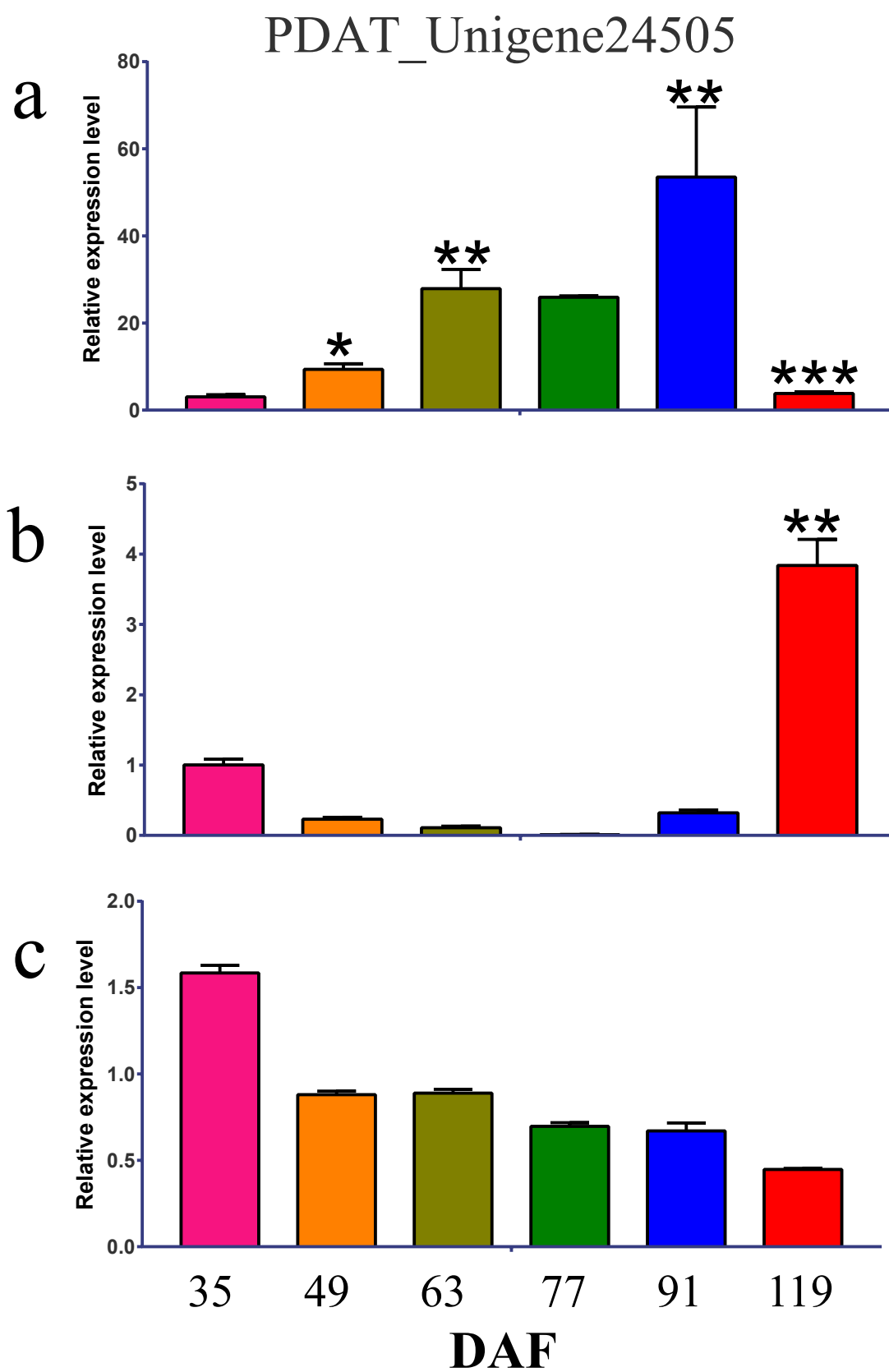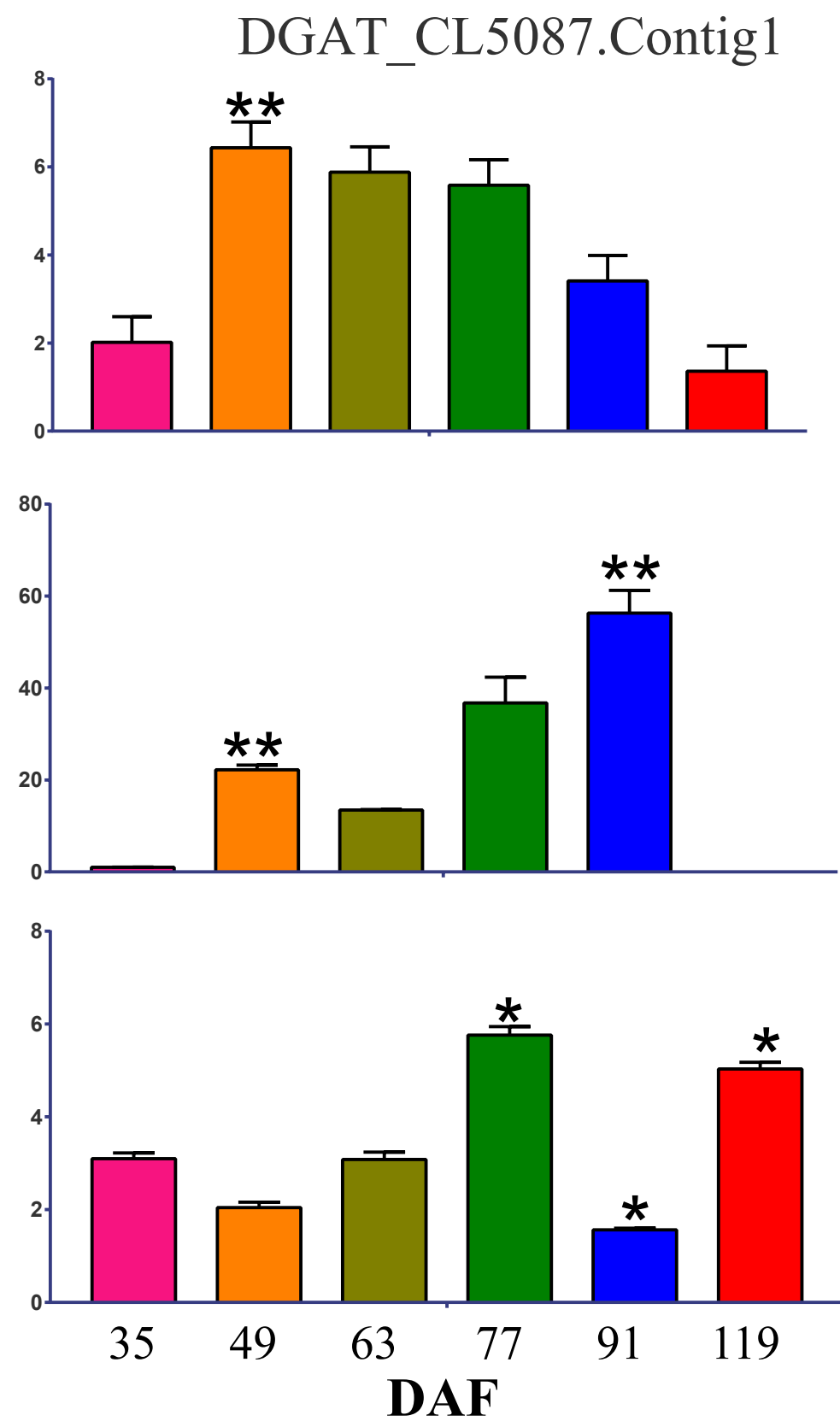

Supplement: Supplementary file 19 — Additional file 19: Figure S14. Quantitative real-time PCR validation of PDAT and DGAT genes. a Kernel. b Testa. c Pericarp. [file 12864_2021_7594_MOESM19_ESM.pdf]

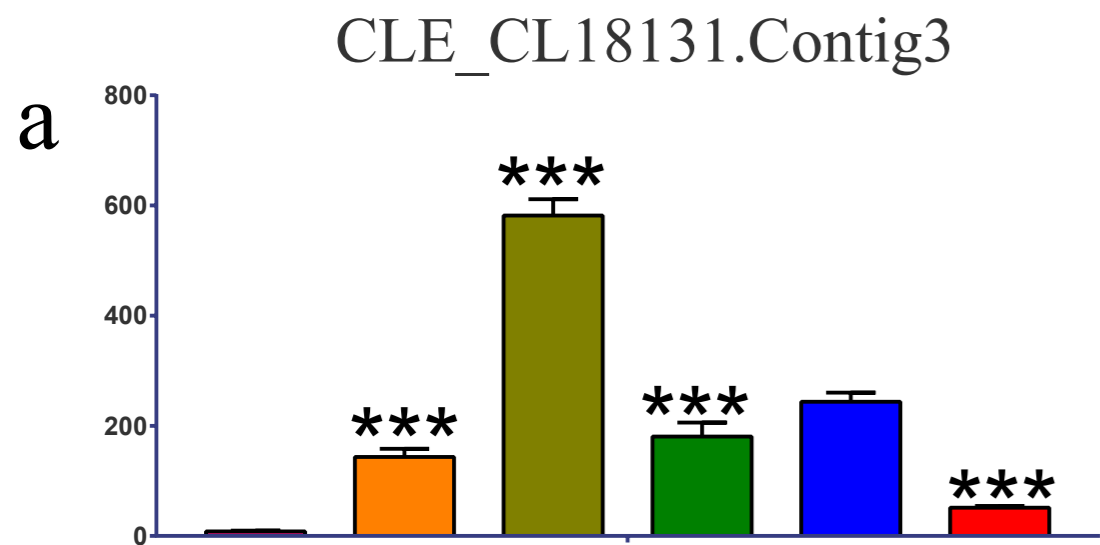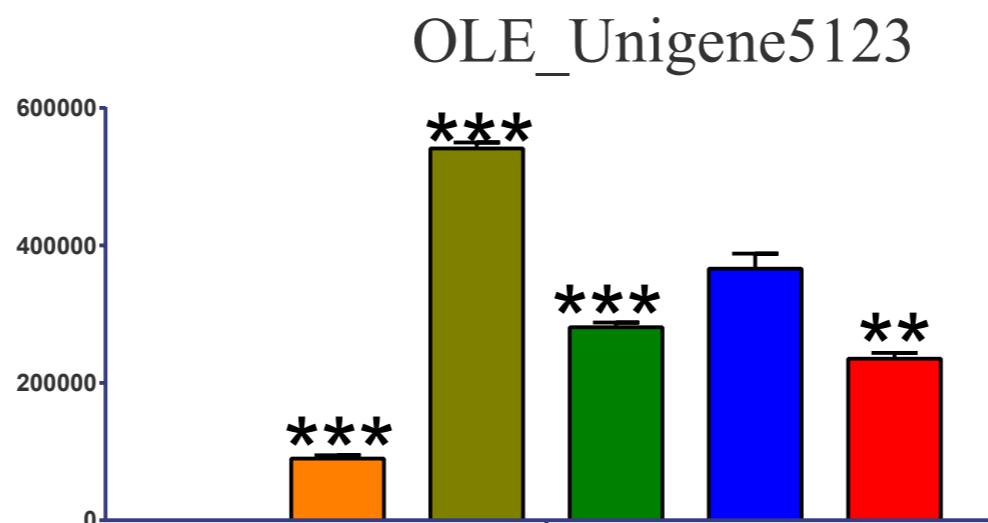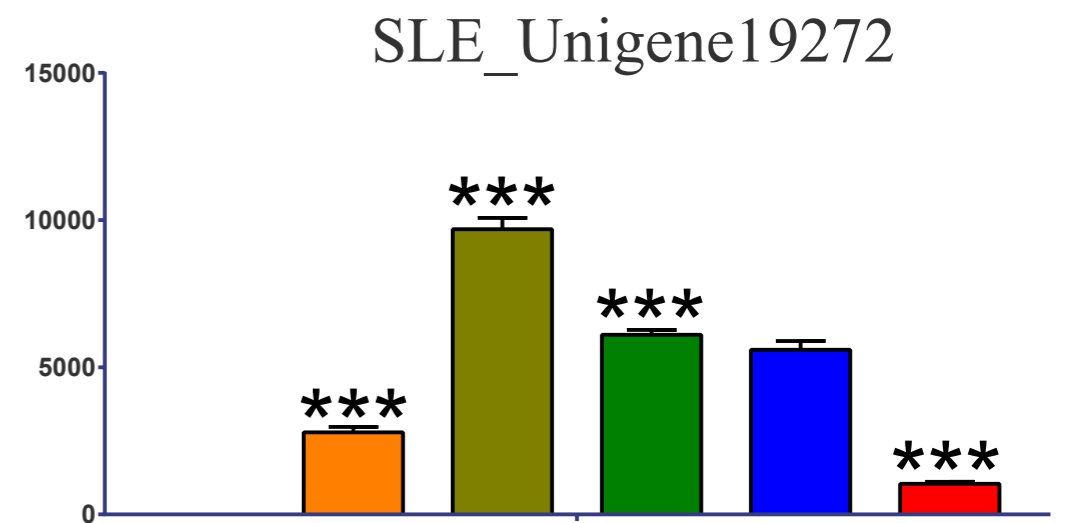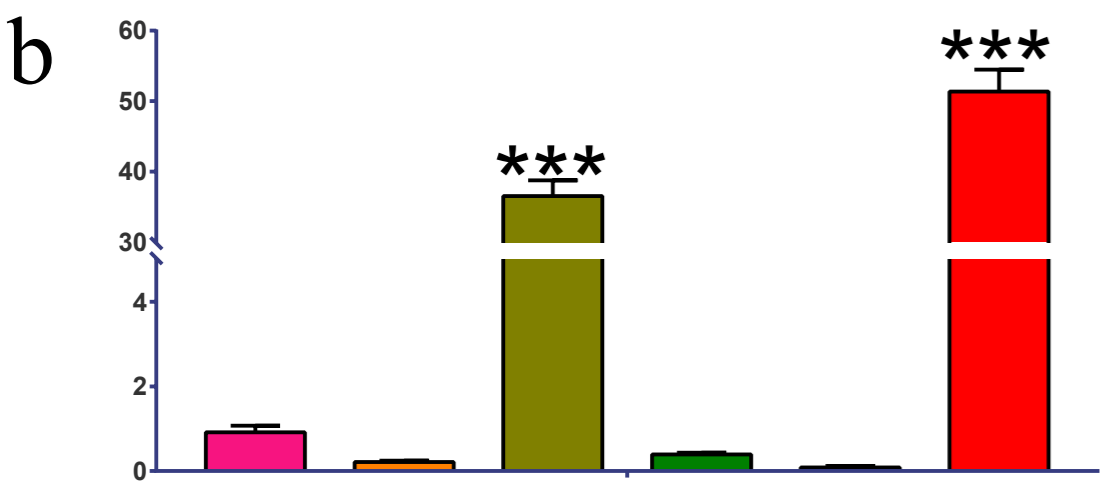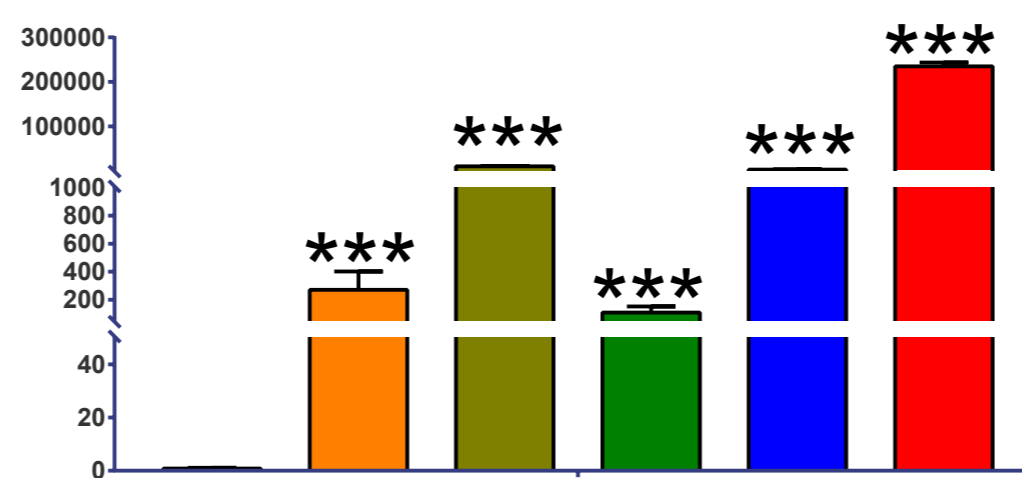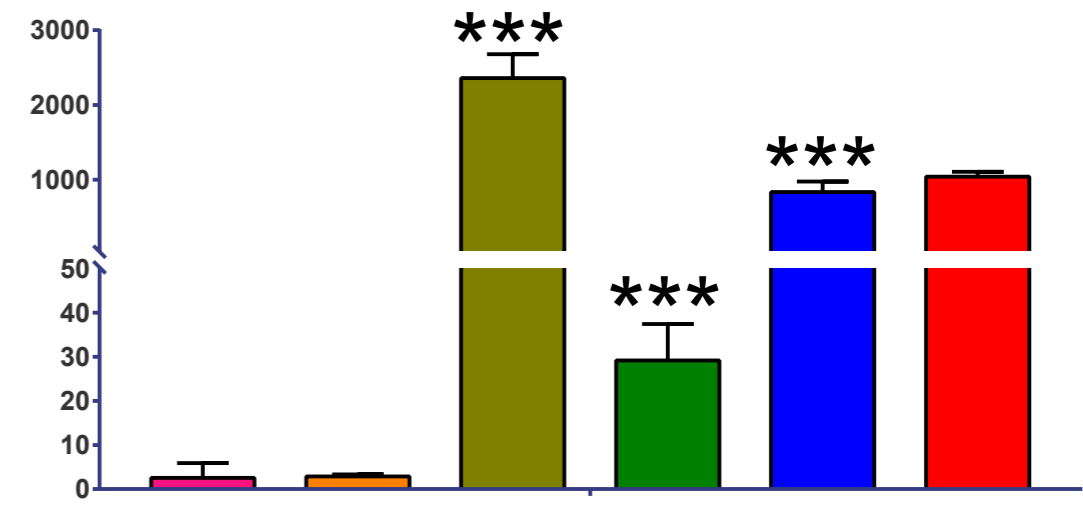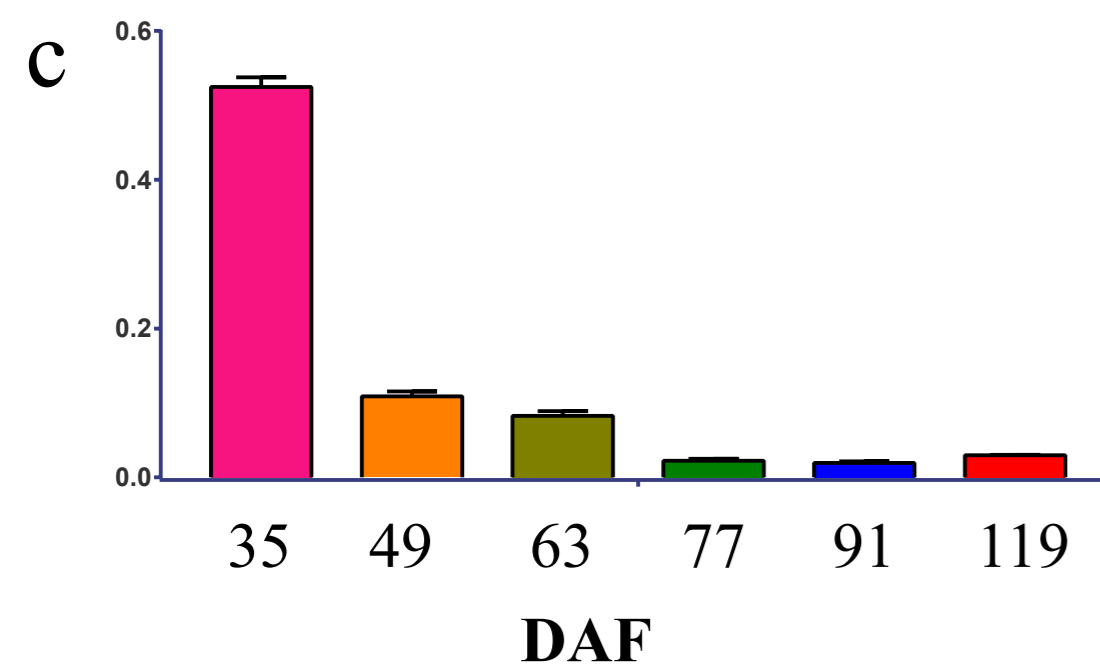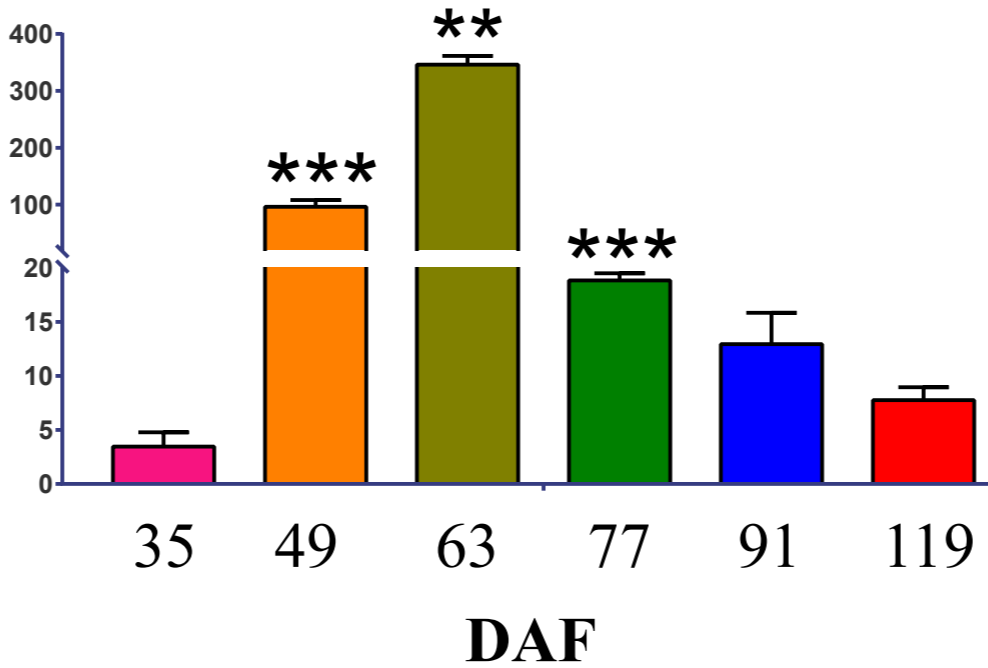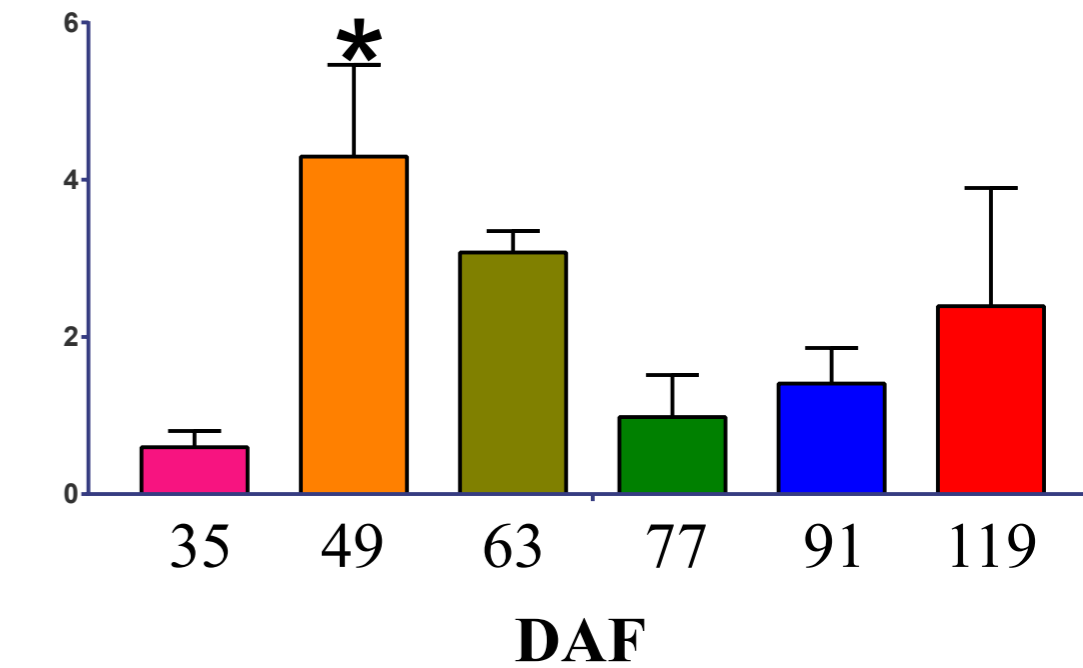

Supplement: Supplementary file 20 — Additional file 20: Figure S15. Quantitative real-time PCR validation of oil-associated genes. a Kernel. b Testa. c Pericarp. [file 12864_2021_7594_MOESM20_ESM.pdf]
