# Supplementary material for: Transcriptomic analysis of α-linolenic acid content and biosynthesis in Paeonia ostii fruits and seeds
Source: BMC Genomics. 2021 Apr 23;22:297. doi: 10.1186/s12864-021-07594-2 (PMC8063412; doi:10.1186/s12864-021-07594-2)
Supplement: Supplementary file 21 — Additional file 21: Table S6. Primer sequences used for quantitative real-time PCR. [file 12864_2021_7594_MOESM21_ESM.pdf]

Table S5 Sequence of primers employed in qRT-PCR

GeneName	GeneID	Forward primer sequence	Reverse primer sequence
α-CT	15202	ACGCTCCCAATTATGCAAGC	TCGCCTTCGCAATTTTCTCC
BCCP	17464	AAGCTTTCAGTTCCCAGCAC	AGCTGAAGGTGCGGAATTTG
BC	7345	AGCCATCAAGCTTGCTCATG	AATGCTGCAATCACGCTCTC
SAD	2824	TGGACATGACGCAAATCGAG	AAGCAGTTTCGTGACGCTTC
FAD2	15521	AAAGCCTCCATTCACGCTTG	AGATTGCAGCCTTGTAGTGC
FAD6	5945	ACAGCTTGGCTTCCTGTTTG	ACGGCCATCCAATTGCAATG
FAD3	1023	CTGACCTGCCCTGGTTGAAT	TCCTGGACTTCTCCACCACA
FAD7/8	21286	GCTGTTCGTTCTTGGCCATG	ACAAGTCACTGCCTGGATGG
DGAT	5087	TGCTCTGTGCTTGCATTGTG	AACCCTTTCGGATGCATGTC
PDAT	24505	TTTTCGGGTGCTTGAACTGC	ACAGCACGAATTCGAATGCC
OLE	5123	TGATAACGGCTGGGCTTTTG	AGCCGTCCATCAAATCAAGC
CLE	18131	CGCGCAATTGGCTTTAATGC	AGTCATGGCCCAAAGTTCAC
SLE	19272	TCGTGCAGATGTTTCCAAGG	TGCCACAATCTTGCCTTTGC
